# Sensitivity of Mass Geometry Parameters on E-Scooter Comfort: Design Guide

**DOI:** 10.3390/s24020399

**Published:** 2024-01-09

**Authors:** Juan David Cano-Moreno, José Manuel Arenas Reina, Victorina del Carmen Parra Lanillos, Manuel Enrique Islán Marcos

**Affiliations:** Escuela Técnica Superior de Ingeniería y Diseño Industrial, Universidad Politécnica de Madrid, 28012 Madrid, Spain; juandavid.cano@upm.es (J.D.C.-M.); vdc.parra@alumnos.upm.es (V.d.C.P.L.); manuel.islan.marcos@upm.es (M.E.I.M.)

**Keywords:** e-scooter, multibody dynamics, ride comfort, mass geometry, sensors

## Abstract

E-scooter vibrations are a problem recently studied. Theoretical models based on dynamic simulations and also real measurements have confirmed the high impact of e-scooter vibrations on driver comfort and health. Some authors recommend improving e-scooter damping systems, including tyres. However, it has not been suggested nor has any research been published studying how to improve e-scooter frame design for reducing driver vibrations and improving comfort. In this paper, we have modelled a real e-scooter to have a reference. Then, we have developed a multibody dynamic model for running dynamic simulations studying the influence of mass geometry parameters of the e-scooter frame (mass, centre of gravity and inertia moment). Acceleration results have been analysed based on the UNE-2631 standard for obtaining comfort values. Based on results, a qualitative e-scooter frame design guide for mitigating vibrations and increasing the comfort of e-scooter driver has been developed. Some application cases have been running on the multibody dynamic simulation model, finding improvements of comfort levels higher than 9% in comparison with the e-scooter reference model. The dynamic model has been qualitatively validated from real measurements. In addition, a basic sensor proposal and comfort colour scale is proposed for giving feedback to e-scooter drivers.

## 1. Introduction

**Figure 1:**
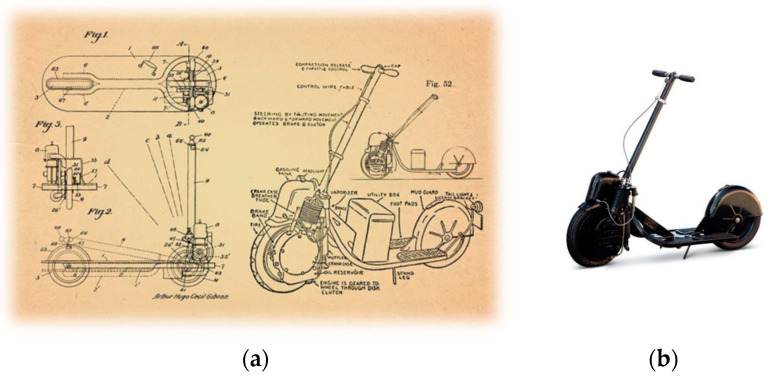
Autoped, 1915: (**a**) patent [3] and (**b**) physical model.

The invention of the self-propelled scooter for adults is more than 100 years old. The invention was born after the patent of the first electric bicycle at the end of the 19th century. This invention is considered the precursor to the electric scooter. In 1915, in the United States, the first self-propelled scooter [1] began to be marketed, although not electric, called Autoped, which had a combustion engine and reached 32 km/h [2], very similar to current e-scooters, with the handlebar also foldable, as Figure 1 shows.

However, it was not until the invention of the first lithium batteries in 1991 when the first e-scooters began to be manufactured, with less autonomy than their predecessors, but with less environmental impact [4]. The access of this vehicle to everyone began with the operation of electric scooter rental companies around the world. Thus, for example, one of the pioneer companies, Lime, began operating in 2017 [5] in 16 countries in America, Asia, Europe and Oceania. The importance of the e-scooter in different parts of the world was growing [6,7,8,9,10] until in 2019 sales multiplied by 10 from the previous year and, as a forecast for the future, sales of this mobility vehicle will continue to increase worldwide [11]. This shows the importance of analysing and optimizing its interaction with the user. The scooter has gone from a small child’s toy to a vehicle that has solved the problem of the last mile and small trips. In many countries, regulations began to be developed after their operation was launched. Most of the published papers related to the e-scooter topic are related to usage patterns of shared e-scooters [12,13,14,15,16] or the analysis of injuries [17,18,19,20], but the literature related to mechanical and dynamical analysis is still scarce.

Most of the first e-scooters did not have an additional damping system for the wheels themselves and some of them still had very rigid wheels. This was the main reason why a methodology was proposed in 2019 [21] to study the impact of vibrations on the drivers of e-scooters to evaluate whether, to what degree and under what conditions the vibrations we receive from these new means of transport influence our comfort and possibly our health, following the UNE-2631 standard [22]. Although there are numerous studies of comfort on other types of vehicles [23,24,25,26,27,28,29], nothing had been published about the need to study vibrations in e-scooters.

Cano-Moreno et al. [30] applied the indicated methodology thanks to previously obtaining average stiffness values in two types of commonly used wheels, rigid and pneumatic. The results showed that for a good or very good level of pavement roughness, an electric scooter began to be no longer comfortable at 16 km/h and harmful to health from 23 km/h, all this for one trip of short duration and the use of inflatable tyres, worsening when switching to rigid tyres or a worse degree of pavement roughness.

**Figure 2:**
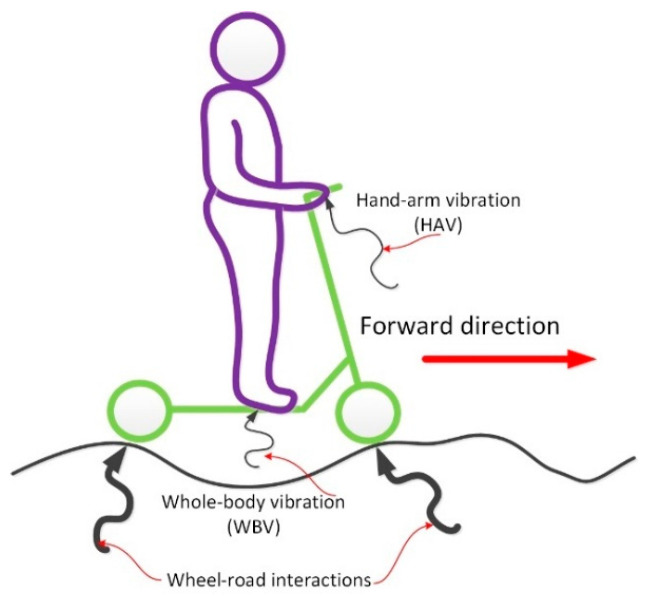
E-scooter vibration scheme [36].

Cafiso et al. [31] measured and compared accelerations on an e-scooter, bicycle and car from a comfort perspective. Boglietti et al. [32] compared vibrational dynamics between e-scooters and e-bikes using UNE-2631, obtaining higher vibration magnitudes for e-scooters. Antoniazzi and Davoli [33] applied the methodology suggested by Cano-Moreno et al. [21] and measured the vibrations received by e-scooters on different pavements and at different speeds to evaluate their influence on driver comfort. Cano-Moreno et al. [34,35] also sensorized a Citycross model scooter, thus obtaining the influence on comfort and health of real measurements of accelerations using an Arduino as data acquisition equipment. The results of all these studies are consistent and coherent with each other and with those obtained in the dynamic simulation model. Figure 2 shows the main sources of vibration for an electric scooter driver.

These research studies suggest that e-scooter design should be improved for reducing vibrations due their impact on driver comfort [21,34] and health [21,36]. They suggest actuating on damping elements, such as tyres or suspensions between the wheels and e-scooter frame. In this line, Ma et al. [36] analysed recently the vibrations for different wheel sizes, finding decreasing vibrations’ impact for higher wheel sizes.

However, it has not been suggested nor has any research studying how to improve e-scooter frame design for reducing driver vibrations and improving comfort been published.

In this paper, we have modelled a real e-scooter to have a reference. Then, we developed a multibody dynamic model for running dynamic simulations studying the influence of the mass geometry parameter of the e-scooter frame (mass, centre of gravity position and lateral inertia moment).

## 2. Methodology

**Figure 3:**
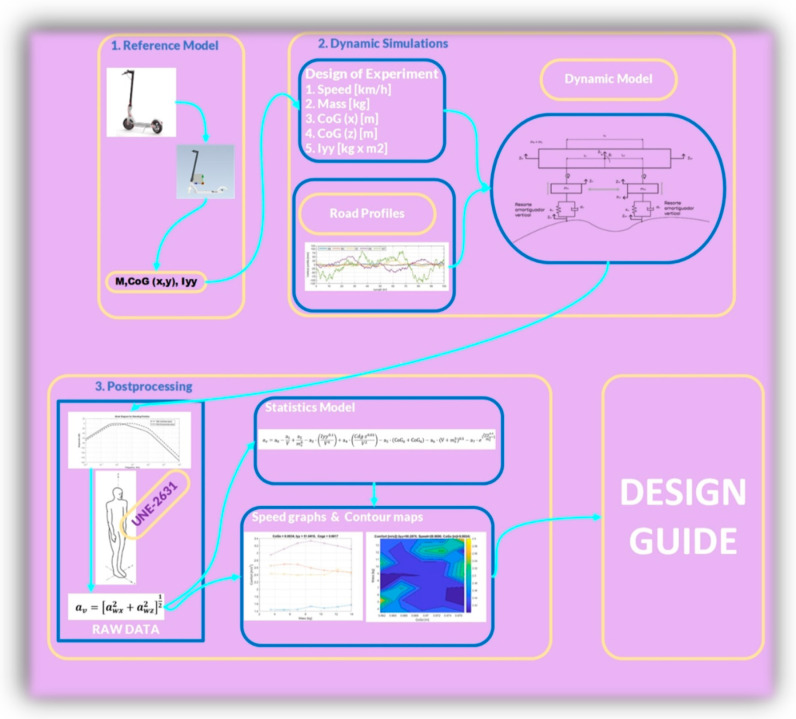
Methodology scheme.

A methodology has been developed to understand the influence of the mass and inertial parameters of the frame of an e-scooter on the driver’s comfort due to the vibrations received through the foot. This methodology will allow the results to be used to develop a design guide for the frame of electric scooters to improve user comfort. Figure 3 shows the main steps and workflow of this methodology.

These steps are briefly described below:

1. Reference model. The starting point was a real e-scooter, which has been modelled in 3D to obtain its values of mass, centre of gravity and moments of inertia to use them as reference values. Driver data will be constant;
2. Dynamic simulations. A dynamic model of multibody systems has been generated to simulate the behaviour of the e-scooter against different vibrations modelled as different vertical road profiles. In this model, the different combinations of parameters defined in the design of the experiment will be simulated;
3. Postprocessing. The accelerations resulting from each simulation will be processed according to the UNE-ISO 2631-1:2008 standar [37] to obtain frequency-averaged values that will allow the degree of comfort to be decided. These values will be used to:
  a. Obtain statistical models by multiple regression;
  b. Obtain graphs from the statistical model and the raw simulation output data:
    i. One variable graph to evaluate the variation of each parameter independently;
    ii. Contour maps (two variable graphs) based on constant comfort lines and colour maps based on the averaged acceleration value.

Finally, with the interpretation of the postprocessed data, the results will be discussed and the design guide for the e-scooter frames will be prepared to improve driver comfort.

### 2.1. E-Scooter Reference Model

**Figure 4:**
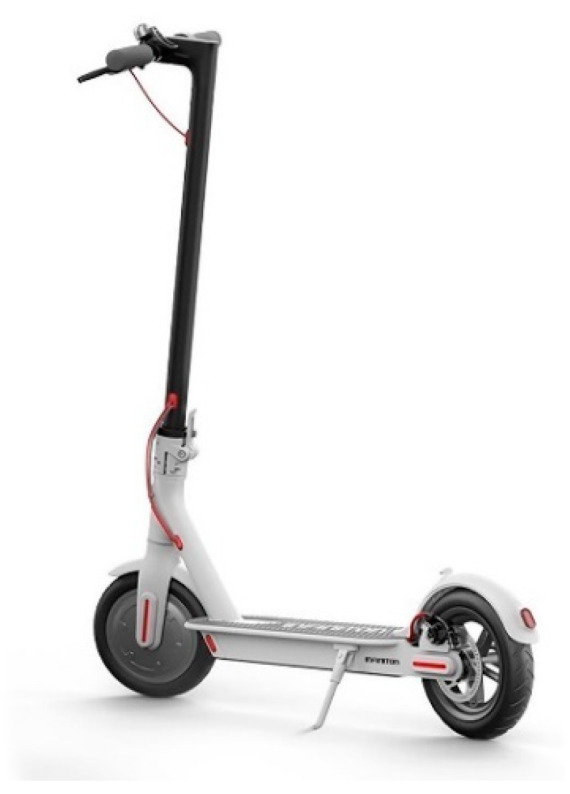
E-scooter model [38].

The INFINITON EASYWAY CITYCROSS model, shown in Figure 4, has been selected as the reference scooter. To model this e-scooter in Inventor, real measurements have been taken on a real e-scooter model available to the Research Group of Industrial Design and Manufacturing from the Escuela Técnica Superior de Ingeniería y Diseño Industrial (ETSIDI) de la Universidad Politécnica de Madrid (UPM).

**Figure 5:**
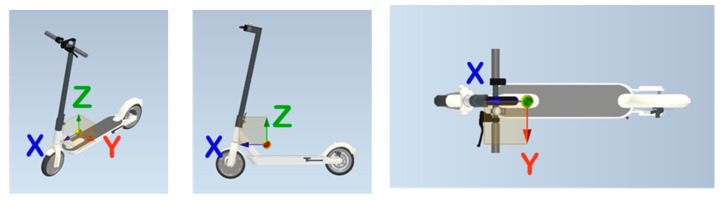
3D model indicating position of the centre of gravity.

Once all the bodies had been modelled and assembled, the complete scooter was obtained, which is shown in Figure 5.

As a verification, the real scooter was weighed with two commercial scales, obtaining a mass of 11.7 kg on both scales. The complete assembled model weighs a total of 11.59 kg, so we consider that both the geometric and material allocation approximation are acceptable (99.1%). The mass and inertia properties of the modelled e-scooter, which were obtained from INVENTOR software (version 2019), are shown in Table 1. A point located in the centre of the scooter’s treadable platform was considered as the origin of the coordinates.

### 2.2. Driver Data

To obtain the mass geometry of the driver, the model has been taken, and a model already used in simulations of electric scooters [30] has been deduced from the inertia data indicated by Chandler [39]. The driver’s position will be standing, with his arms stretched out to hold the handlebars. In this case, the arms, compared to the rest of the body, would practically have no influence on the result, so their position is ignored. The model selected for the simulation is that of a woman from northern Europe, 71 kg and 1.67 m tall, whose mass geometry is shown in Table 2.

### 2.3. Comfort Evaluation

To study the comfort of the user of an electric scooter, use will be made of the UNE-2631 standard, a standard that deals with mechanical vibrations and shocks and the evaluation of human exposure to whole-body vibrations.

**Scheme 1:**
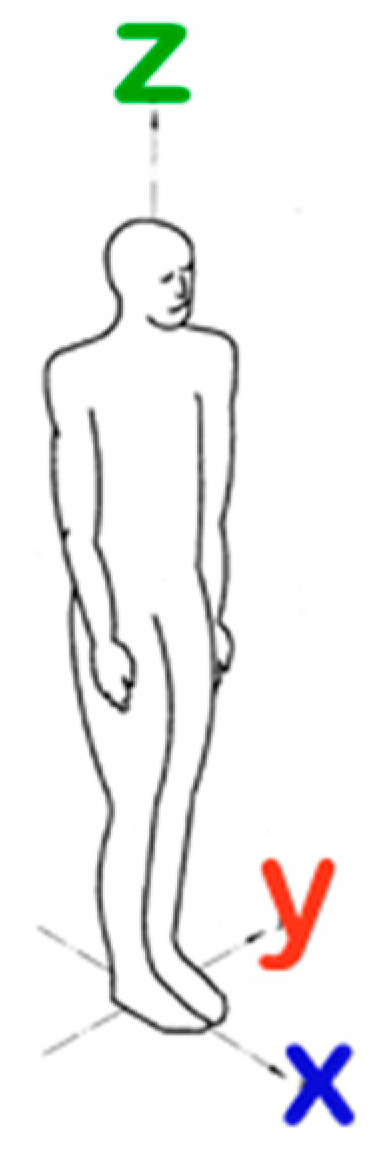
Standing position according to UNE-2631 standard [37].

In this standard, acceleration is established as the primary quantity of vibrations, and the origin of the coordinate system is the point from which the vibrations enter the human body. In the case of calculating the vibrations that are transmitted to a person in a standing position, as is the case of the driver of an electric scooter, the standard stipulates that the measurements must be made on the surface on which the feet are [37] (see Scheme 1).

For a correct evaluation of vibration following the standard, the RMS value of the weighted acceleration ($a_{w}$) has to be calculated. The standard UNE-2631 defines the proper filters for calculating in standing position the acceleration weighed values. These filters are also described by Cano-Moreno et al. [21]. The $a_{w}$ value will be calculated according to the following equation:

(1) $$a_{w}={\left[\;\frac{1}{T}\int_{0}^{T}a_{w}^{2}\left(t\right)dt\;\right]}^{\frac{1}{2}}$$

Once we obtain the values of the RMS weighted acceleration, we can use the comfort scale of the standard UNE-2631, as indicated in Table 3.

### 2.4. Multibody Dynamic Model

A multibody model has been implemented in Simscape, a Simulink library (in Matlab 2018b version). The next subsections will define the different parts of the multibody dynamic model used.

#### 2.4.1. Topologic Diagram

**Figure 6:**
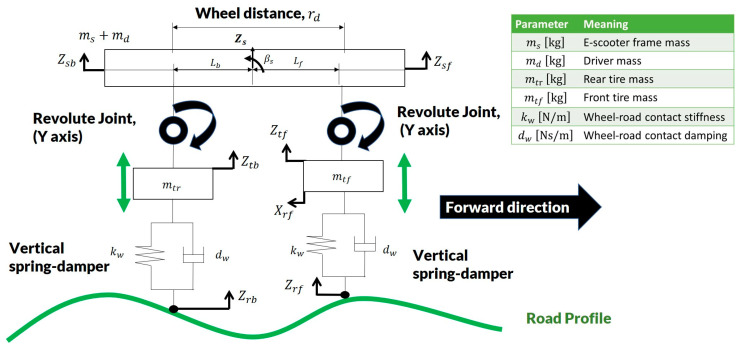
Topologic diagram of dynamic model.

This model includes bodies and their properties (mass, centre of gravity coordinates and inertia tensor) but also joints to define relative allowed movement between bodies and force elements-type spring-damper elements. Figure 6 shows the topologic diagram of the multibody dynamic model. This model runs like a 2D model, with tree mail rigid bodies: front and rear wheels ($m_{tf}$ and $m_{tr}$) and a body including e-scooter frame ($m_{s}$) and driver body ($m_{d}$) joined as a rigid solid.

#### 2.4.2. Simulation Data

**Figure 7:**
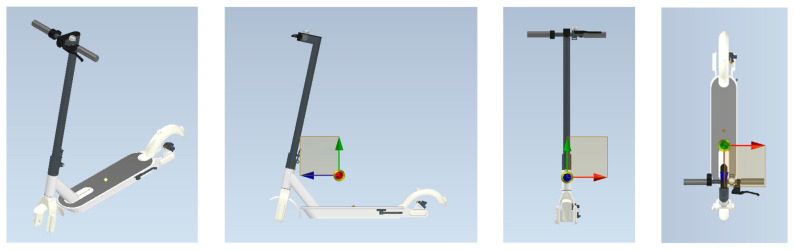
E-scooter frame 3D model.

For the simulation, the weight of the wheels is entered independently, both front and rear, as is the structure of the scooter, that is, handlebars and base. Therefore, it is important to know the properties of the mass geometry of the e-scooter chassis or frame. Figure 7 shows only the frame of the e-scooter without wheels.

Considering the same origin of coordinates as before, they are shown in Table 4.

In the dynamic simulation model, the driver is considered rigidly attached to the base of the scooter. To obtain the geometric mass properties of the complete model, that is, of the scooter frame plus its driver, the data entered in Table 4 (frame) are considered to complete Table 5, where the properties of frame and driver all together as an unique rigid solid have been calculated:

To obtain some of these values, the Steiner theorem [40] has been used, as shown in the following equations:

(2) $$Z_{g}=\frac{Z_{g_{\mathrm{driver}}}\cdot {\;m}_{c}+Z_{g_{\mathrm{frame}}}\cdot m_{e}}{m_{c}+{\;m}_{e}}=\frac{0.7181\cdot 71+0.172\cdot 6.961}{71+6.961}=0.6693\;m$$

(3) $$X_{g}=\frac{X_{g_{\mathrm{driver}}}\cdot {\;m}_{c}+X_{g_{\mathrm{frame}}}\cdot m_{e}}{m_{c}+{\;m}_{e}}=\frac{0\cdot 71+0.077\cdot 6.961}{71+6.961}=0.0069\;m$$

(4) $$I_{\mathrm{xx}}=I_{\mathrm{xx}}\left(\mathrm{CdG}\right)+m_{c}\cdot Z_{g}^{2}=11.7+71\cdot {\left(0.7181\right)}^{2}=48.31\;\mathrm{kg}\cdot m^{2}$$

(5) $$I_{\mathrm{yy}}=I_{\mathrm{yy}}\left(\mathrm{CdG}\right)+m_{c}\cdot Z_{g}^{2}=13+71\cdot {\left(0.7181\right)}^{2}=49.61\;\mathrm{kg}\cdot m^{2}$$

For the simulation model, the geometric properties of the wheels have also been obtained from the point of origin taken for the simulation, that is, from the centre of the base, where the driver’s feet would rest. The properties are summarized in Table 6.

The stiffness of the wheels has been extracted from the model from the average data obtained experimentally and described in the study of Cano-Moreno et al. [30]. In this, wheels are studied, such as those of the scooter model used in this case, that is, tyres with an inflatable air chamber, whose rigidity varies from 0.9·10^5^ a 1.3·10^5^ N/m depending on the inflation pressure of the wheels, from 30 to 60 psi, respectively. In this case, an inflation pressure of 60 psi for the front wheel and 50 psi for the rear wheel has been considered, which represents a vertical stiffness of 1.3 10^5^ N/m for the front wheel and 1.2 10^5^ N/m for the rear wheel. In the case of tyres damping, Heißing and Ersoy [41] have indicated that the damping value of a tyre is almost negligible and that values of 50 Ns/m or 100 Ns/m can be used. In this paper, the value of 100 Ns/m has been used for all simulations.

#### 2.4.3. Road Profiles

Vertical road profiles have been defined as a random road profile according to ISO 8608 [42]. In this paper, only two best road classes have been considered (A and B). Table 7 shows the values of the power spectral density of vertical displacement (referred to as Φ in this research) according to reference values of spatial frequency, *n*_0_ = 0.1 cycles/m and the angular spatial frequency, *Ω*_0_ = 1 rad/m. 

We are going to consider a mix of these road classes for defining a very good–good road for simulations considering the value of Φ (*Ω_0_*) = 2∙10^−6^ m^3^. 

Likewise, the road profiles defined by Cano-Moreno et al. [30] have been used, for a length of 300 m per simulation. In this paper, random road profiles are generated as a sum of sine functions, as described by Tyan et al. [43]. This sum provides the elevation value of the road, $h\left(x\right)$, for each value of length traveled, s.

(6) $$h\left(s\right)=\sum_{i=1}^{i=N}A_{i}\cdot sin\left(\Omega_{i}\cdot s-\varphi_{i}\right)$$

where

- *φ_i_* is the random phase angle that follows a uniform probabilistic distribution within the interval $\left[0,\left.2\pi \right)\right.$;
- Ω*_i_* is the angular spatial frequency i, which for N points will have a value of
  (7) $$\Omega_{i}=\Omega_{1}+\Delta \Omega \cdot \left(i-1\right)\;\left[\mathrm{rad}/m\right],\;\mathrm{con}\;\Delta \Omega =\frac{\Omega_{N}-\Omega_{1}}{N-1}\;\left[\mathrm{rad}/s\right]$$

Next, values $\Omega_{1}=0.02\cdot \pi \;\left[\mathrm{rad}/m\right]$ and $\Omega_{N}=6\cdot \pi \;\left[\mathrm{rad}/m\right]\;\mathrm{have}\;\mathrm{been}\;\mathrm{selected}$. If $A_{i}$ is the amplitude that is defined according to the following equation:

(8) $$A_{i}=\sqrt{\Phi \left(\Omega_{i}\right)\frac{\Delta \Omega }{\pi }},\;\;\;i=1,\;\ldots ,\;N$$

In the previous equation, $\Phi \left(\Omega_{i}\right)\;\left[m^{2}/\left(\mathrm{rad}/m\right)\right]$, are the values of the Power Spectral Density (PSD) of displacement for the angular spatial frequency $\Omega_{i}$. Now, Equation (4) in its general form is:

(9) $$\Phi \left(\Omega_{i}\right)=\Phi \left(\Omega_{0}\right)\cdot {\left(\frac{\Omega_{i}}{\Omega_{0}}\right)}^{-w}$$

**Figure 8:**
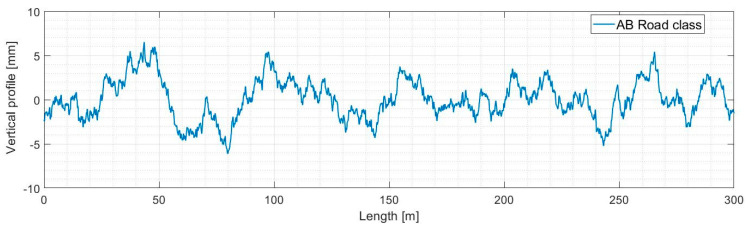
Road profile example (300 m).

The value $\Phi \left(\Omega_{0}\right)$ defines the PSD value for the reference wave value $\Omega_{0}=1\;\mathrm{rad}/m$. In this paper, only one type of road is going to be studied (AB class, with $\Phi \left(\Omega_{0}\right)=2\cdot 10^{-6}\;m^{3}$. The w value corresponds to the undulation value, the value of 2 being normally accepted for most road surfaces. This will generate a vertical road profile that will be travelled at 5 constant velocities (5 km/h, 10 km/h, 15 km/h, 20 km/h and 25 km/h). Figure 8 shows an example of a random vertical profile generated for the AB road class during 300 m.

### 2.5. Brief of Mass Geometry

In the dynamics of the rigid solid [44] magnitudes called centres of gravity, moments of inertia appear, which are directly related to how the mass is geometrically distributed in space. The study and calculation of these magnitudes is called “mass geometry” [45]. The three-dimensional centre of gravity position of a ‘rigid solid’ is given by the vector ${\left(\vec{r_{g}}\right)}^{\;\to }$ of Equation (10), with M being the total mass of the system.

(10) $$\vec{r_{g}}=\frac{1}{M}\int_{M}\vec{r}\cdot dm$$

The moment of inertia conceptually measures the degree of dispersion of masses with respect to a point, line or plane. If r is the distance of the mass of the rigid solid considered from the element studied (point, line or plane), in a generic way, the moment of inertia can be formulated as:

(11) $$I=\int_{M}r^{2}\cdot dm$$

In the dynamics of the rigid solid, both the centre of gravity and the moments of inertia take on special importance. A rigid solid can be considered as a point mass concentrated at the centre of mass and with the inertial properties of the rigid solid. In the case of the dynamic model implemented in Matlab, it is a simulation model of multibody systems where each body is a rigid solid joined by joints, restrictions or forces to the rest of the bodies. In this case, the model is two-dimensional in the axis perpendicular to that plane, that is, $I_{yy}$, which can be formulated as follows:

(12) $$I_{yy}=\int_{M}\left(x^{2}+z^{2}\right)\cdot dm$$

This is intended to emphasize the relationship between the coordinates of the centre of gravity, from where this main moment of inertia, $I_{yy}$, is considered and the mass of the rigid solid being studied. These variables, along with speed, will be studied as proposed in the following section.

### 2.6. Design of Experiment

The design of the experiment aims for the results obtained to evaluate the sensitivity of the different characteristic parameters of the e-scooter frame to the driver’s comfort. For this, the following parameters related to the mass geometry and that have influence on the implemented 2D dynamic model will be studied independently. Table 8 shows the variations in mass, centre of gravity and moment of inertia with respect to the lateral axis and the percentage of variation they represent with respect to the model value taken as a reference (0%). The simulation values for the rigid solid formed by the e-scooter frame and human can be calculated with these values and using Equations (2) and (3) for coordinates of centre of gravity. The mass will be added to the human mass, and the lateral inertia moment is presented in this table also for the total rigid solid.

The mass has been varied from −50% to +100%, with a total of 7 levels. This parameter has more levels due to its greatest influence in relation to others. The coordinates of the centre of gravity in both x and z and the value of the moment of inertia of the axis perpendicular to the simulation plane have been varied from −50% to 50% of each reference value, with each one being divided into 5 levels. Additionally, each combination of these 875 combinations will be simulated for each of the following 4 speeds: 10 km/h, 15 km/h, 20 km/h and 25 km/h. This means that in total 3500 different combinations will be simulated.

## 3. Results

### 3.1. Statistical Model

After running a full factorial design of experiment, a non-linear multiple regression model has been developed. The presented model reaches an adjusted R-square value of 98.4%, as shown in the next equation:

(13) $$a_{v}=13.4577-\frac{373,537}{V}+\frac{215,027}{m_{s}^{6}}-5078550000\cdot \left(\frac{Iyy^{0.1}}{V^{9}}\right)+3377.8\cdot \left(\frac{Cdg\;z^{0.01}}{V^{2}}\right)-0.494219\cdot \left({\mathrm{CoG}}_{z}+{\mathrm{CoG}}_{x}\right)\;-0.000147025\cdot {\left(V+m_{s}^{5}\right)}^{0.5}-0.303662\cdot e^{\left(\frac{Iyy^{0.5}}{m_{s}^{2}}\right)}$$

where

- $a_{v}$, weighted acceleration [$m/s^{2}$];
- $m_{s}$, e-scooter frame mass [kg];
- V, e-scooter longitudinal speed [km/h];
- Iyy, lateral axis inertia moment [$\mathrm{kg}\cdot m^{2}$];
- $CoG_{z},$ z (vertical) coordinate of centre of gravity [m];
- $CoG_{x},$ x (longitudinal) coordinate of centre of gravity [m].

As can be seen, the model presents a high non-linearity in all the used variables. This explains why, in the following sections, graphical results from this model and also from the raw results of the dynamic simulations are used with the aim of also comparing both sources.

### 3.2. Graphical Results

**Figure 9:**
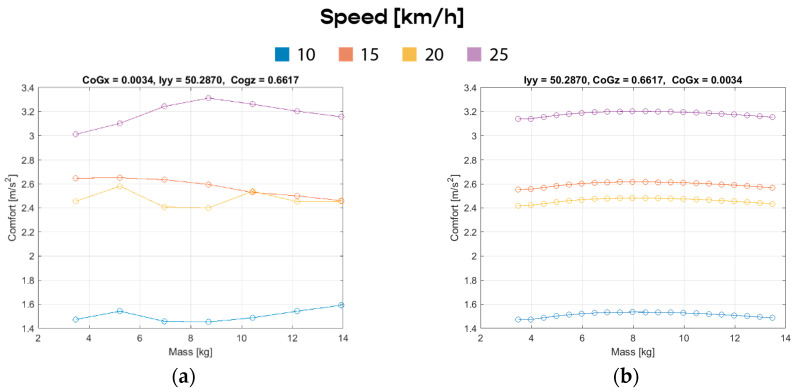
Comfort variation depending on frame mass (**a**) from raw data and (**b**) from statistical model.

Given the high non-linearity of the statistical model obtained, it has been graphically represented how each variable varies as a function of speed for each variable, in addition to obtaining the contour maps for each pair of variables. This has been accomplished with the statistical model obtained and with the raw results of the simulations. Thus, graphs have been obtained that show the variation of each variable depending on each parameter defined in the design of the experiment for all the variations of the rest of the parameters (a total of 162 figures). An example of this type of graph for the variation of the mass of the e-scooter frame is shown in Figure 9. In this case, the variation in comfort is observed as a function of speed by varying the mass for specific values of $CoG_{x}$, $CoG_{z}$ and Iyy. The graph obtained from the statistical model and from the raw data has been represented.

**Figure 10:**
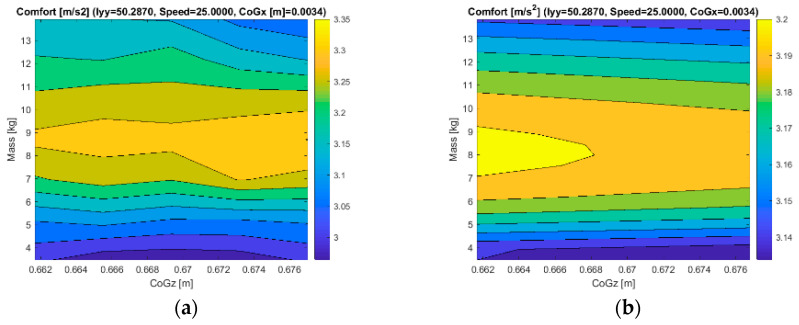
Comfort acceleration ($a_{v}$) variation using contour maps depending on frame mass and CoGz position (**a**) from raw data and (**b**) from statistical model.

In addition, contour maps have been represented for analysing simultaneously the interaction of two parameters and comfort level. These graphical results have been studied also for both statistical and raw data results (a total of 312 figures have been collected in the Supplementary Materials). Figure 10 shows the contour maps representing how comfort values vary with the interaction of mass and CoGz values. These kinds of results for each combination of pairs of parameters and the variation of the others have been obtained by following the hereinbefore described design of the experiment. This figure represents both results from statistical model and simulation raw data.

All these graphical results have been attached as Supplementary Materials.

## 4. Discussion: Design Guide

### 4.1. Analysis of Graphical Results

In this section, graphical results obtained by graphical results derived from statistical and simulation raw data results will be analysed. Both graphical sources are going to be compared.

**Figure 11:**
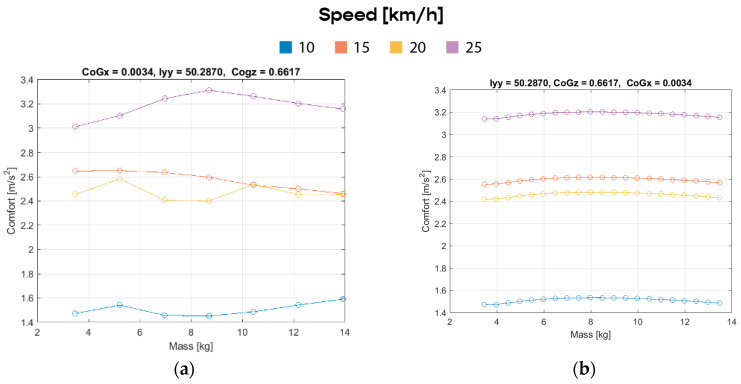
Comfort acceleration and e-scooter frame mass variation depending upon different speeds (**a**) from raw data and (**b**) from statistical model.

**Figure 12:**
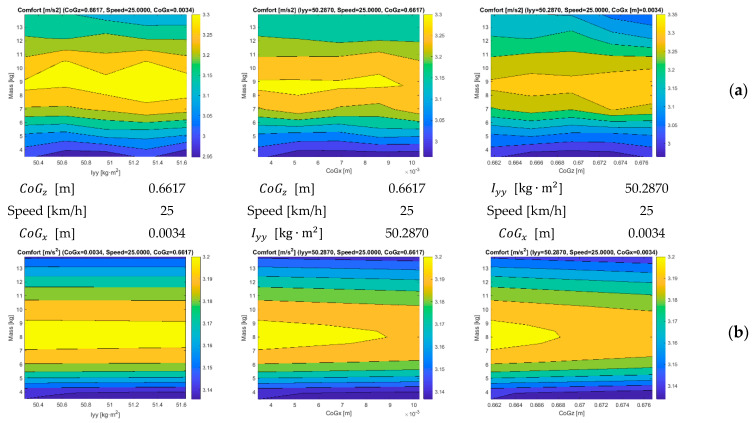
Contour maps for comfort acceleration and mass variation depending upon lateral inertia moment and centre of gravity coordinates (**a**) from raw data and (**b**) from statistical model.

**Figure 13:**
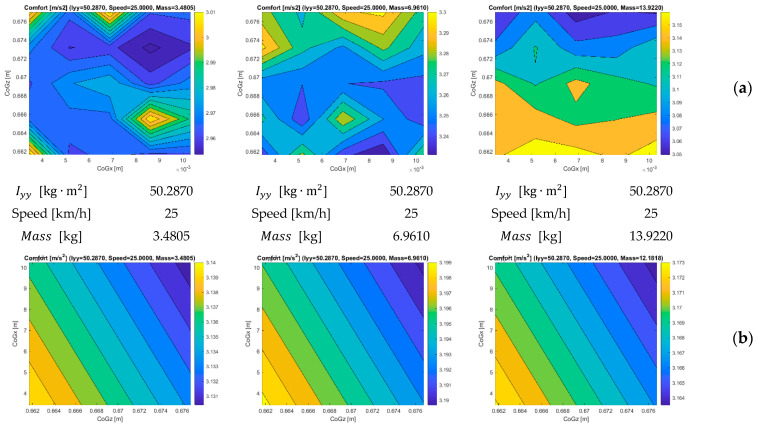
Comfort acceleration depending upon longitudinal and vertical coordinates of the centre of gravity (**a**) from raw data and (**b**) from statistical model.

1. It is observed how the increase in mass causes a maximum of 8.4 kg, and the minimum value of comfort acceleration (*a_v_*) is achieved with 3.48 kg. Figure 11 shows an example of this tendency in both graphs (a) from raw data and (b) from the statistical model.

a. Mass is the second variable with the most weight after speed. Depending upon this, it has greater or lesser influence, as Figure 12 shows.
b. Increasing mass may cause graphs to become more regular for the simulation raw result. This effect is not appreciated though contour maps obtained from the statistical model, as can be seen in Figure 13.

**Figure 14:**
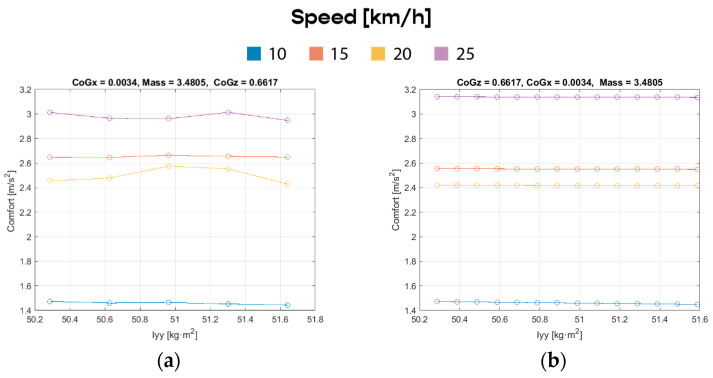
Comfort acceleration depending upon $I_{yy}$ and different speeds (**a**) from raw data and (**b**) from statistical model.

2. A higher value of *I_yy_* leads to lower values of comfort acceleration (*a_v_*), although it is not a linear variation. Figure 14 shows an example for a 3.48 kg e-scooter frame mass.

**Figure 15:**
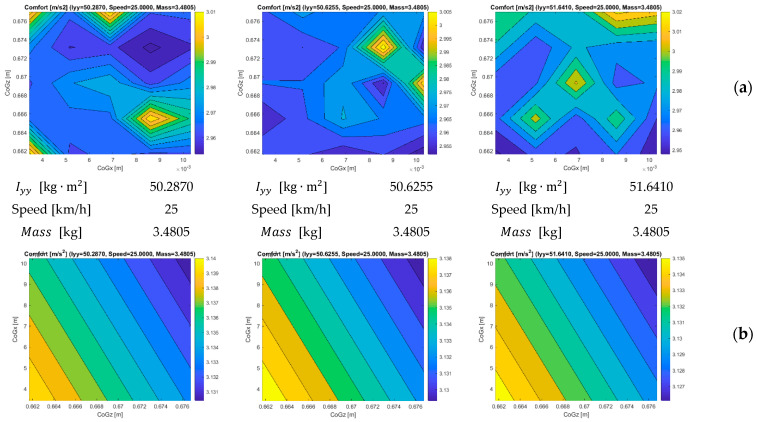
Comfort acceleration depending upon longitudinal and vertical coordinates of the centre of gravity ($CoG_{x}$ and $CoG_{z}$) for several values of $I_{yy}$ (**a**) from raw data and (**b**) from statistical model.

**Figure 16:**
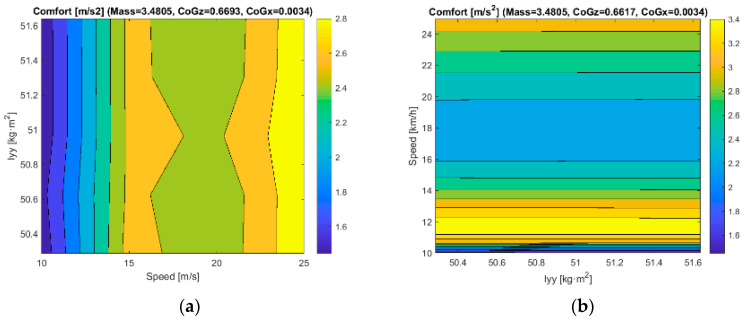
Comfort acceleration depending upon lateral inertia moment ($I_{yy}$) and speed (**a**) from raw data and (**b**) from statistical model.

**Figure 17:**
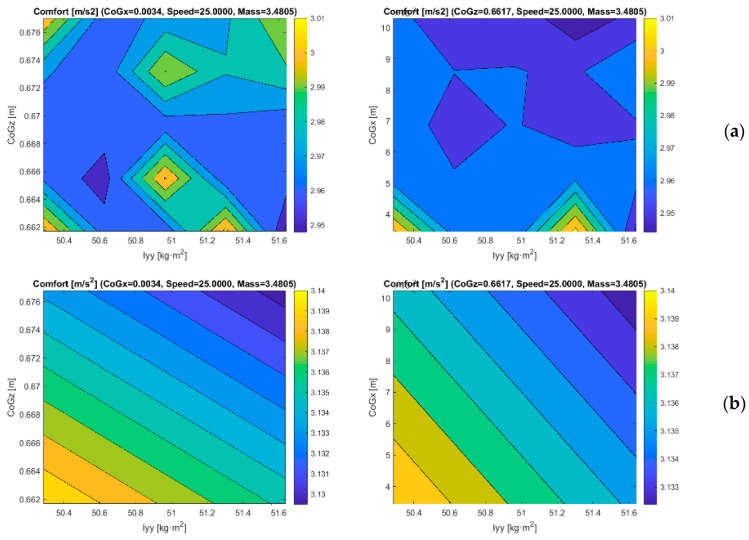
Comfort acceleration depending lateral inertia moment ($I_{yy}$) and vertical coordinate of centre of mass ($CoG_{z}$) for (**a**) from raw data and (**b**) from statistical model.

Simulation results (raw data) show that the change in lateral inertia moment, $I_{yy}$, causes irregularities to move within the contour maps, as can be seen in the Figure 15a. The statistical model filters these effects (see Figure 15b).

a. The influence of $I_{yy}$ is more relevant for speeds higher than 15 km/h (see an example for a 3.48 kg e-scooter frame mass in Figure 16). The effect is similar in both results (a) from raw data and (b) from the statistical model.
b. In raw data contour maps, the influence of $I_{yy}$ does not have a linear variation linked to $CoG_{z}$ and $CoG_{x}$, but it causes areas of favourable or unfavourable combinations with this, as Figure 17 shows. This tendency is filtered into contour maps of the statistical model.

**Figure 18:**
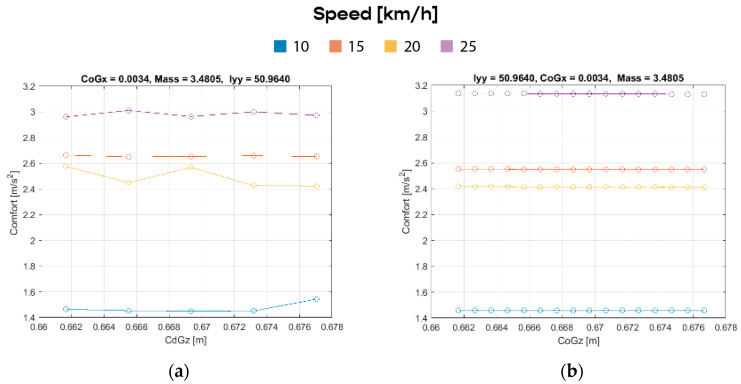
Comfort acceleration depending upon vertical coordinate of centre of mass ($CoG_{z}$) at different speeds (**a**) from raw data and (**b**) from statistical model.

The variation of $CoG_{z}$ is very irregular and depends on the combination of other elements. This parameter does not have a regular behaviour, and we are not going to include it in the design guide as a tendency. Figure 18 shows a random tendency in raw results (a) and, on the other hand, horizontal lines for each speed (b) from the statistical model.

**Figure 19:**
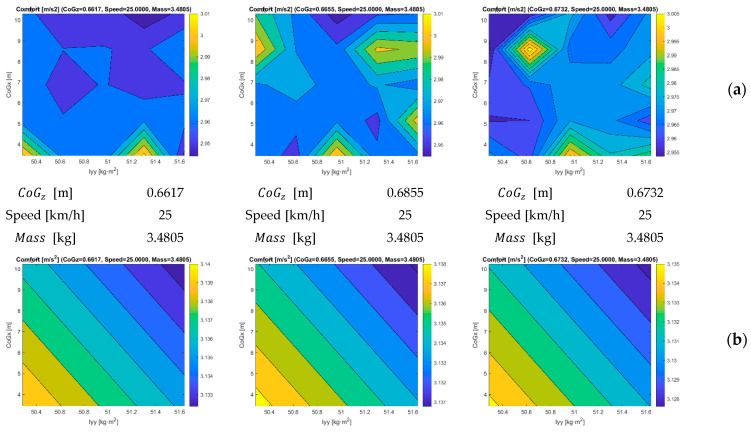
Comfort acceleration depending upon lateral inertia moment ($I_{yy}$) and longitudinal coordinate of centre of mass ($CoG_{x}$) (**a**) from raw data and (**b**) from statistical model.

**Figure 20:**
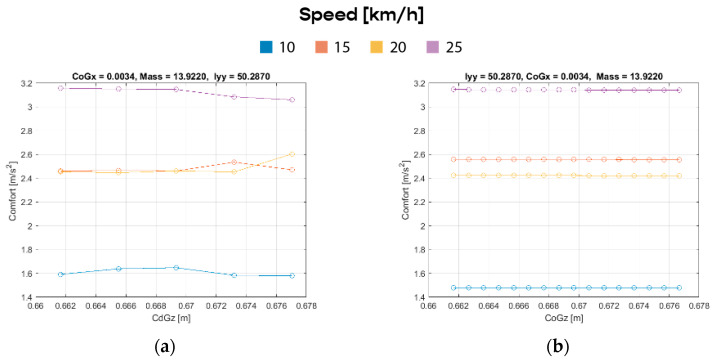
Comfort acceleration depending upon $CoG_{z}$ at different speeds (**a**) from raw data and (**b**) from statistical model.

(a) Based on the raw data of simulation results, changing $CoG_{z}$ causes the irregularities that move with the change of other parameters. Figure 19a shows this in the variation of $CoG_{x}$ and $I_{yy}$. Raw data contour maps also show the existence of regions with better behaviour than others. The contour maps provided from the statistical model filters this effect. However, these contour maps show the worst comfort behaviour for the middle value of $CoG_{z}$.

3. It is observed how the increase in $CoG_{x}$ causes a slight decrease in the value of comfort acceleration ($a_{v}$) at least for the maximum speed, 25 km/h (Figure 20). These results are obtained from both raw data (a) and from the statistical model (b).

The conclusions drawn from these results from the raw simulation data and the developed statistical model are similar; however, more details and more complex conclusions are drawn from those drawn from the raw simulation results. This also validates the statistical model obtained.

### 4.2. Design Guide

Based on the graphical analysis of the previous section, a list of technical points to consider for improving the comfort of e-scooters varying only the e-scooter frame geometry mass properties has been collected. Although the results and statistical model have a high non-linearities, some technical guides for increasing driver comfort considering the change of one parameter remaining constant the others can be concluded:

1. Decrease the mass of the e-scooter frame. Probed up to 50%;
2. Increase the transversal inertia moment $I_{yy}$. Probed up to 50%;
3. Decrease/increase the height of the centre of gravity, $CoG_{z}$. Probed ±50%;
4. Increase the longitudinal position of the centre of gravity, $CoG_{x}$.Probed up to 50%;
5. Combine points 1 to 4, reaching the maximum comfort acceleration and maximum driver comfort for the combination of the four points;
6. If there were some difficulties in achieving points 1–4 in the e-scooter frame design, increasing the mass up to double (100% of increment), increasing to 50% of $I_{yy}$ and decreasing to 50% of $CoG_{x\;}\mathrm{could}\;\mathrm{be}\;\mathrm{tried}$.

We have considered that the change in one mass geometry parameter normally is not a direct action because each parameter can influence the value of the others. As an example, if we decrease the mass by decreasing the density value of all the e-scooter frame, by changing material properties for example, we find that the centre of gravity will maintain its position, but the transversal inertia moment will decrease as well. Thus, we should try to increase the degree of mass dispersion in order to increase the inertia moment value. These changes, however, could also change the previous centre of gravity position. Other ways to decrease the e-scooter mass could be adding holes somewhere in the e-scooter frame or redesigning completely the e-scooter frame (widths, materials, shapes), considering the influence in each case on the other parameters.

### 4.3. Application Cases

**Figure 21:**
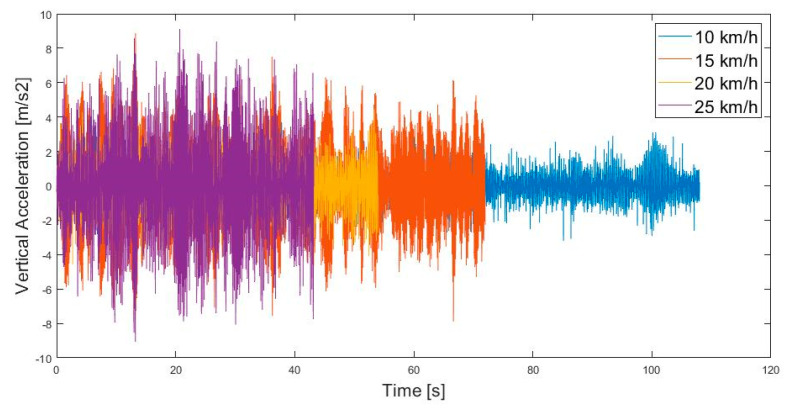
Simulation results (vertical acceleration, raw data) with original geometric properties (reference model).

First of all, the results that would be given in the case of the geometric properties of the original scooter are introduced (Figure 21).

The geometric properties in this case have been collected in Table 9:

As application cases, four have been defined in which only one parameter is varied, six cases in which two parameters have been varied, four cases with variations of three parameters and one in which all four parameters have been varied simultaneously. In these examples, the improvement (decrease) in the indicator value of the vibrations associated with the driver’s comfort, comfort acceleration ($a_{v}$), is compared with the reference value previously indicated. In Table 10, you can see in the main diagonal the improvement results for the modification in the simulation model of a single parameter, while the rest of the boxes show the improvement results for comfort acceleration ($a_{v}$) for the rest of the combination of cases, where two parameters have been varied simultaneously. The highest value is obtained for a mass reduction of 50%, although, as indicated, in a real design it cannot be changed directly while keeping the rest of the parameters constant.

Table 11 shows each combination of three and four parameters used as an application case for this design guide. The best value is obtained for changing the four parameters simultaneously.

As the mass parameter has a maximum, it has also been simulated by increasing the mass by 100% (doubling the mass). The values obtained are lower than those shown in Table 10 and Table 11. The maximum value if the mass is increased to 100% is 7.32% if $CoG_{x}$ is reduced to 50% and $I_{yy}$ is increased to 50%. On the other hand, as the variation of $CoG_{z}$ does not have a clear variation, the influence on comfort acceleration if $CoG_{z}$ increases its value to 50% has also been calculated. The best combination would be if mass is 50% reduced and $CoG_{x}$, $CoG_{z}$ and $I_{yy}$ are 50% increased, reaching a reduction in comfort acceleration of 8.83%. Thus, depending on how successful the task design following the design guide would be, both options (increase or decrease $CoG_{z}$) should be considered.

The presented results show combinations of parameters that will provide an improvement in driver comfort of more than 9% in several cases, all having as a common denominator the 50% reduction in the mass of the scooter frame. The case with the highest percentage of improvement is the result of changing the four parameters simultaneously.

### 4.4. Validation and Sensor Basic Proposal

**Figure 22:**
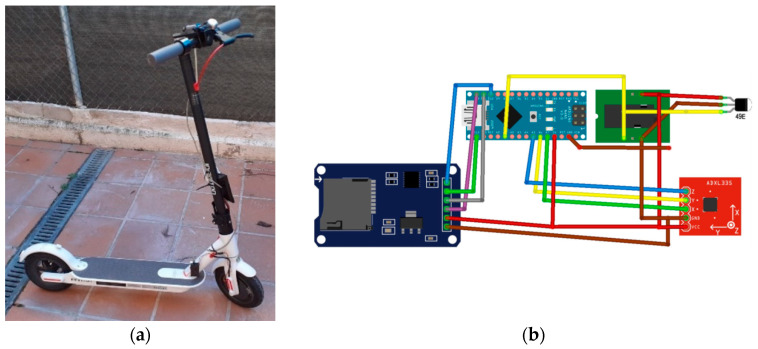
Sensorized e-scooter [34]: (**a**) real e-scooter and (**b**) sensorization scheme used.

The presented results and other published studies [34,36,46] about the impact of accelerations on e-scooter driver comfort present the high importance of received vibrations. In a previous study [34], the comfort for this e-scooter reference model was sensorized and measured by using an Arduino Nano 3.x microcontroller, such as a Data Acquisition System (DAS), and using 200 Hz of sampling frequency. In this research, longitudinal speed (using AH49E Hall effect linear magnetic sensor and neodymium magnet) and triaxial accelerations (using low-cost sensors for triaxial accelerations, ADXL335) were measured. Figure 22 shows (a) this model with sensors and DAS installed and (b) the hardware sensorization scheme for measuring and recording received signals.

These real measurements were made for speeds starting at 20 km/h to 28 km/h and two types of road (asphalt and pavers). The closest case to the simulated conditions is called maximum speed that normally reaches around 25–28 km/h. For these cases, the average comfort values, including starting and stopping, was between 2.6 and 4.5 m/s^2^ depending on the driver. The obtained value for 25 km/h in this paper was 3.26 m/s^2^, an intermediate value. The main differences could be in driver data and road definition. Ventura et al. [46] measured ride comfort comparing a similar e-scooter model with a bike. They tested several types of pavement. The closest to a good road are bituminous conglomerate and alternated dirt and concrete roads. The mean RMS acceleration values are from 2.62 m/s^2^ to 3.37 m/s^2^ for speeds between 10 and 15 km/h. Thus, higher values are expected when speed increases. Antoniazzi and Davoli [33] applied the methodology of Cano-Moreno et al. [21] and measured the vibrations of e-scooters to study their influence on driver comfort. The obtained results for 25 km/h vary from 1.95 m/s^2^ to 4.76 m/s^2^ for uniform asphalt and asphalt with cracks, respectively.

The three real measurements shown make coherent the obtained value for the dynamic model and qualitatively validate the dynamic model.

In the analysed literature, it is demonstrated that for each e-scooter model, the received vibration level depends on several changing parameters, such as speed, quality of road or even user parameter for sharing e-scooters.

Meanwhile, new generations and designs of e-scooters are going to be developed. It seems recommendable to report on time feedback about the comfort level to e-scooter drivers. Thus, it is proposed for existing models to add an acceleration sensor that could be placed below the foot of driver, fixed to the e-scooter frame just below the central zone of the e-scooter treadable zone, in the centre if possible. The sensor could be a low-cost sensor, such as the ADXL335 triaxial accelerometer used in other e-scooter experiments [34]. E-scooter manufacturers should power this sensor and read it at least at 80 Hz [34] (200 Hz if possible [37]) and postprocess the accelerations following the recommendations of the standard UNE-2631 [37], explained hereinbefore. Based on Table 3, where the comfort scale has been collected, we propose here different colours for advising the e-scooter driver about the received vibration. The colour scale is presented in Table 12.

## 5. Conclusions

A design methodology for mitigating vibrations associated with the comfort of the driver of an e-scooter has been presented throughout the article. This methodology is based on the study of the sensitivity of the mass geometry parameters of the e-scooter to the impact of the vibrations when driving an electric scooter. The methodology uses a dynamic simulation model of an e-scooter based on real data from a type of e-scooter widely used today.

The main conclusions of this research are listed below:

- Inertial parameters have a very non-linear and complex relationship with user comfort, which makes the study of these parameters and how they affect comfort quite difficult;
- Speed has the greatest weight in user comfort. Then, mass stands out as an influencing factor, followed by the moment of inertia $I_{yy}$ and the centre of gravity z, $CoG_{z}$, followed by the mass and finally the centre of gravity x, $CoG_{x}$, whose influence is small;
- A design guide for the electric scooter frame has been developed that will reduce the impact of the vibrations received on the driver’s comfort. In this guide, the indications on each independent parameter will be difficult to carry out since the change of any of the mass geometry parameters can have an influence on the rest;
- The results show that the vibrations associated with the comfort that drivers of this type of scooters currently receive could be improved by more than 9%. This improvement could be achieved with several actions on the mass geometry parameters, such as simply lowering the mass of the scooter frame by 50% and keeping the rest of the parameters constant (centre of gravity and lateral moment of inertia);
- The proposed dynamic model has been qualitatively validated based on the results of real measurements taken in similar models;
- A basic sensor proposal and colour scale for ride comfort has been proposed due to the variability of vibrations for different reasons (mainly e-scooter model, user, speed and road quality). This is a recommendation for e-scooter manufacturers.

The study is limited to a single e-scooter model; in other cases with other scooter models, the results could be different due to both the morphology of the scooter and its components, such as suspensions or tyres. Likewise, the variations of the inertial parameters are limited, and there may be cases where with values lower or higher than those studied, better results would be obtained.

However, the methodology presented here is suitable for use on other scooter models, including other types of vehicles, even with other types of suspension systems, such as spring-damper systems and/or other types and sizes of tyres. The study methodology presented here based on mass geometry is novel, and no articles have been found that use these variables exclusively for the optimization of a vehicle from the point of view of mitigation of received vibrations.

The most immediate future work is to try to implement the conclusions of the design guide in a new electric scooter structure design that meets these characteristics and improves driver comfort. Other planned works include a variation in user data for searching for more robust designs, something very relevant in shared e-scooters.

## Figures and Tables

**Table 1:** Inertia and mass properties of modelled e-scooter [full model].

Mass [kg]	11.593	
Centre of gravity coordinates [m]	X	0.002
	Y	0.082
	Z	0.139
Inertia moments [kg·m^2^]	Ixx	1.828
	Ixy	0.781
	Iyy	1.084
	Ixz	0.001
	Iyz	−0.282
	Izz	1.029

**Table 2:** Driver inertia properties.

Properties	Values
**Centre of gravity height [cm]**	71.81
**Mass [kg]**	71
**I_xx_ [kg·m^2^]**	11.7
**I_yy_ [kg·m^2^]**	13
**I_zz_ [kg·m^2^]**	1.5

**Table 3:** Comfort scale based on UNE-2631 standard.

$a_{w}$ (m/s^2^)	Comfort Scale
**<0.315**	Not uncomfortable
**0.315–0.63**	Slightly uncomfortable
**0.5–1**	Fairly uncomfortable
**0.8–1.6**	Uncomfortable
**1.25–2.5**	Very uncomfortable
**>2**	Extremely uncomfortable

**Table 4:** Inertia and mass properties of modelled e-scooter [frame].

Mass [kg]	6.961	
**Centre of gravity coordinates** **[m]**	X	0.077
	Y	0.003
	Z	0.172
**Inertia moments** **[kg·m^2^]**	Ixx	1.005
	Ixy	−0.001
	Iyy	1.354
	Ixz	−0.356
	Iyz	−0.01 kg m^2^
	Izz	0.383 kg m^2^

**Table 5:** Full model inertia properties.

Mass Geometry Properties	Frame	Driver	Total
**Mass [kg]**	6.961	71	77.961
**Coordinate centre of mass in Z [m]**	0.172	0.7181	0.6693
**Coordinate centre of mass in X [m]**	0.077	0	0.0069
**I_xx_ [kg·m^2^]**	1.005	48.31	49.315
**I_yy_ [kg·m^2^]**	1.354	49.61	50.964
**I_zz_ [kg·m^2^]**	0.383	1.5	1.883
**I_xy_ [kg·m^2^]**	−0.001	0	−0.01
**I_xz_ [kg·m^2^]**	−0.356	0	−0.001
**I_yz_ [kg·m^2^]**	−0.01	0	−0.356

**Table 6:** Properties of e-scooter wheels.

Properties		Rear Whell	Front Wheel
Mass [kg]		1.002	3.63
Centre of gravity coordinates [m]	X	−3.91	0.399
	Y	−0.04	0
	Z	−0.06	0.06
Inertia moments [kg·m^2^]	Ixx	0.006	0.02
	Ixy	−0.002	0
	Iyy	0.160	0.601
	Ixz	−0.024	0.086
	Iyz	0	0
	Izz	0.155	0.584

**Table 7:** ISO 8608 values of $\Phi \left(n_{0}\right)$ and $\Phi \left(\Omega_{0}\right)$ [42].

Road Class	$\Phi \left(n_{0}\right)\;\left(10^{-6}\;m^{3}\right)$		$\Phi \left(\Omega_{0}\right)\left(10^{-6}\;m^{3}\right)$	
	Lower Limit	Upper Limit	Lower Limit	Upper Limit
**A**	-	32	-	2
**B**	32	128	2	8
	$n_{0}=0.1\;\mathrm{cycles}/m$		$\Omega_{0}=1\mathrm{rad}/m$	

**Table 8:** Design of experiment.

Parameters	Values [% Ref]						
**%**	−50%	−25%	0% Ref	+25%	+50%	+75%	+100%
**Mass [kg]**	3.4805	5.22075	6.961	8.70125	10.4415	12.18175	13.922
**CoG z [m]**	0.086	0.129	0.172	0.215	0.258		
**CoG x [m]**	0.00385	0.005775	0.0077	0.009625	0.01155		
**Iyy [kg·m^2^** **]** **Frame/Total**	0.677/ 50.2870	0.677/ 50.6255	1.354/ 50.9640	1.6925/ 51.3025	2.031/ 51.6410		

**Table 9:** Geometric properties and comfort of the original scooter at 25 km/h.

$a_{v}\;[m/s^{2}$]	Speed [km/h]	$I_{yy}$$\;[kg\cdot m^{2}$]	$CoG_{z}$ [m]	$CoG_{x}$ [m]	Mass [kg]
**3.26**	25	50.964	0.6693	0.0069	6.961

**Table 10:** Comfort improvement for the modification in the simulation model of a single parameter.

Improvement	↓50% Mass	$↑50\%\;I_{yy}$	$↓50\%\;CoG_{z}$	$↑50\%\;CoG_{x}$
** ↓50% mass **	9.37%	-	-	-
** $↑50\%\;I_{yy}$ **	7.72%	0.8%	-	-
** $↓50\%\;CoG_{z}$ **	9.28%	0.14%	0.67%	-
** $↑50\%\;CoG_{x}$ **	9.16%	0.47%	0.69%	0.01%

**Table 11:** Comfort improvement varying one or two parameters simultaneously.

Cases	↓50% Mass	$↑50\%\;I_{yy}$	$↓50\%\;CoG_{z}$	$↑50\%\;CoG_{x}$	Improvement
**01**	x	-	x	x	9.34%
**02**	x	x	-	x	8.94%
**03**	x	-	x	x	9.44%
**04**	-	x	x	x	0.17%
**05**	x	x	x	x	9.53%

**Table 12:** Comfort colours scale.

$a_{w}$ (m/s^2^)	Comfort Colour Scale
**<0.63**	Green
**0.63–1.25**	Orange
**>1.6**	Red

## Data Availability

Data are contained within the article.

## Supplementary Materials

The following supporting information can be downloaded at https://www.mdpi.com/1424-8220/24/2/399/s1, Graphical Results.
