# Supplementary material for: Sensitivity of Mass Geometry Parameters on E-Scooter Comfort: Design Guide
Source: Sensors (Basel). 2024 Jan 9;24(2):399. doi: 10.3390/s24020399 (PMC11154470; doi:10.3390/s24020399)

1 Graphical results

1.1 Comfort variation for each variable

1.1.1 Statistical model

1.1.1.1 Variation with I_{yy}

1.1.1.1.1 Varying CoGz

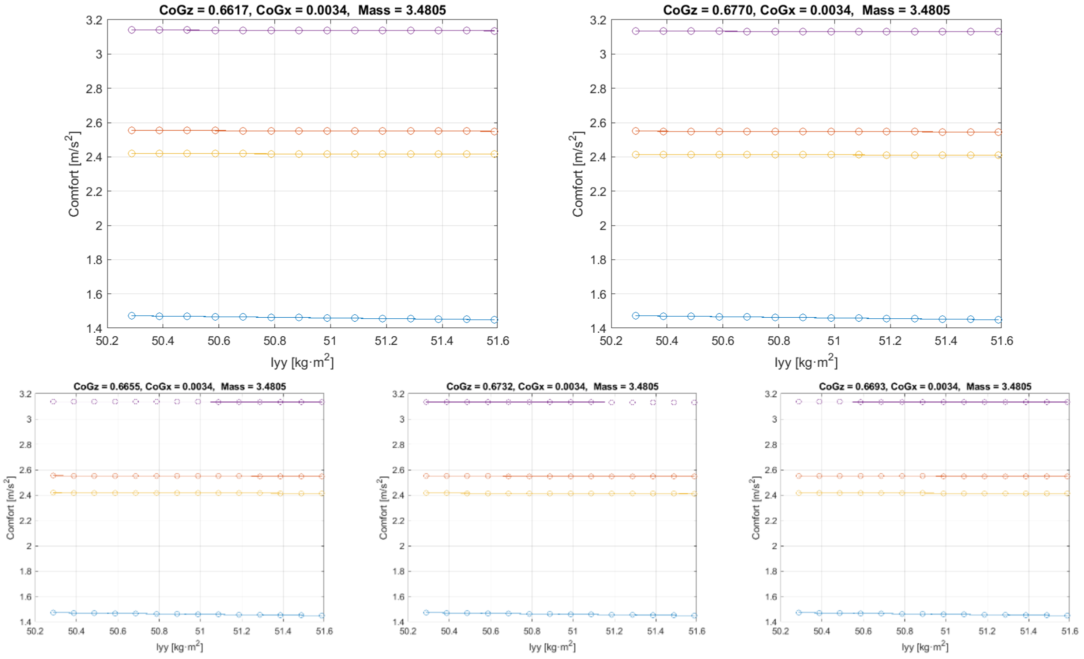

1.1.1.1.2 Varying CoGx

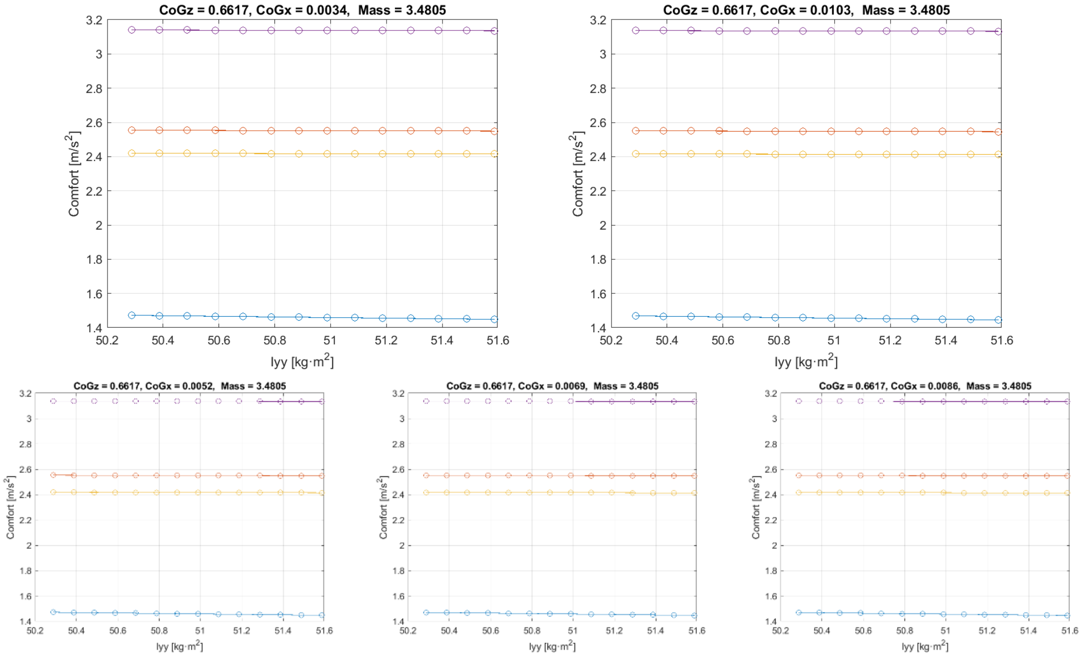

1.1.1.1.3 Varying mass

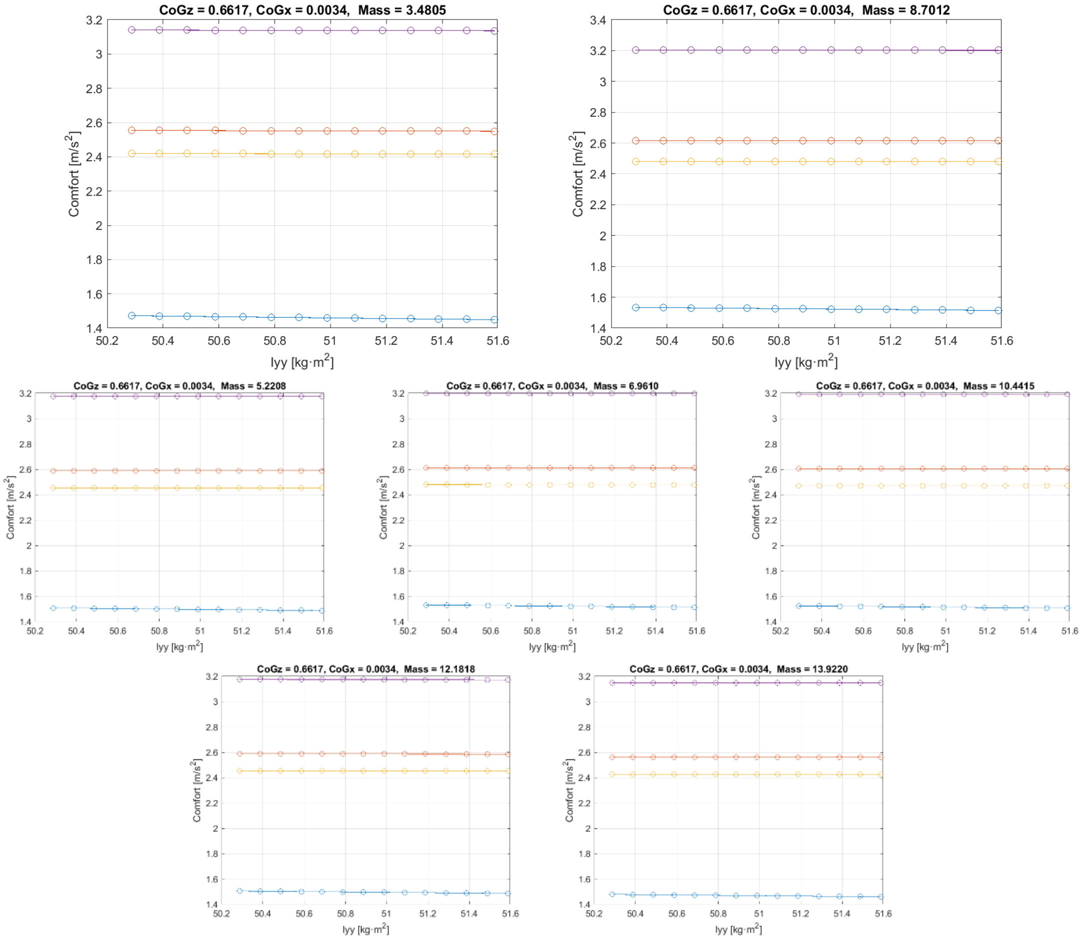

1.1.1.2 Variation with CoGz

1.1.1.2.1 Varying I_{yy}

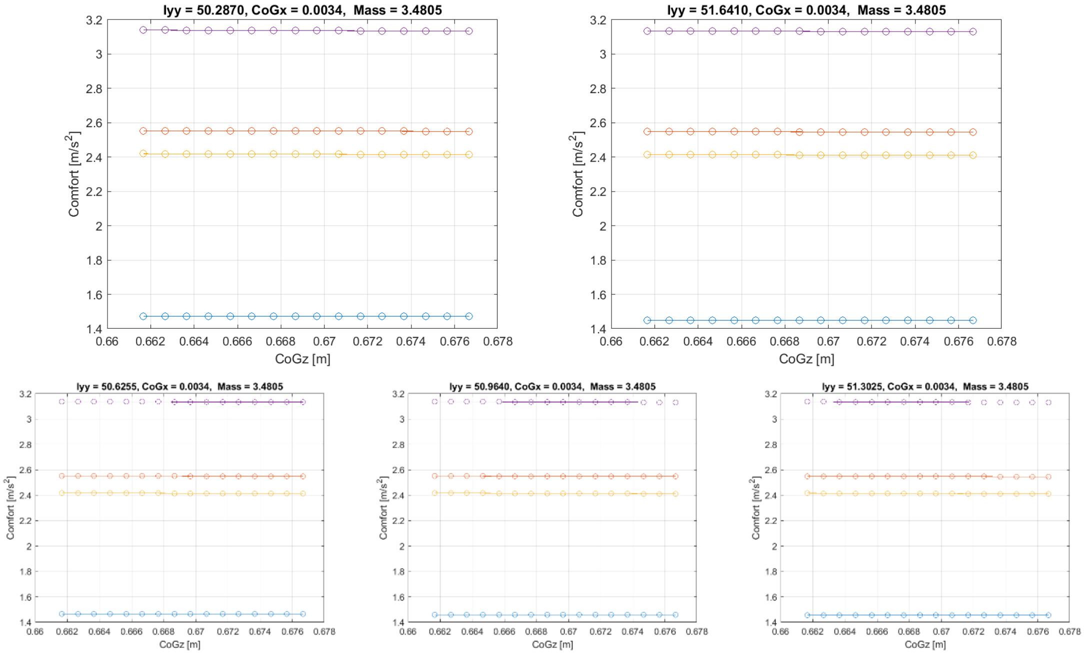

1.1.1.2.2 Varying CoGx

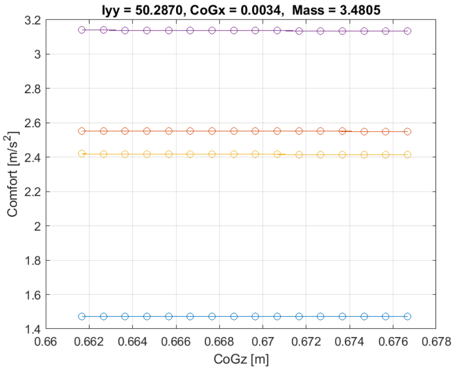

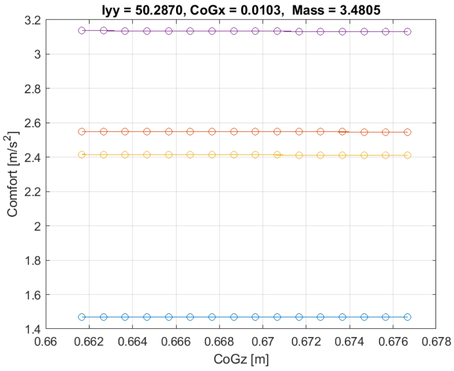

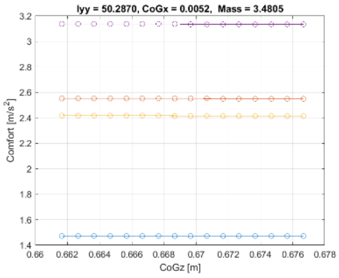

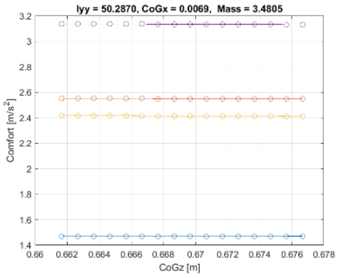

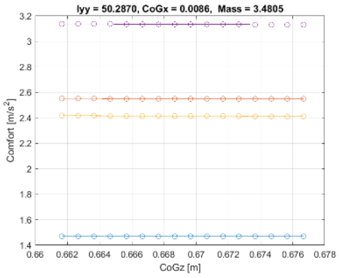

1.1.1.2.3 Varying mass

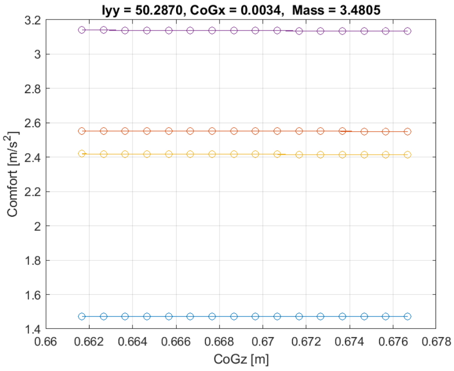

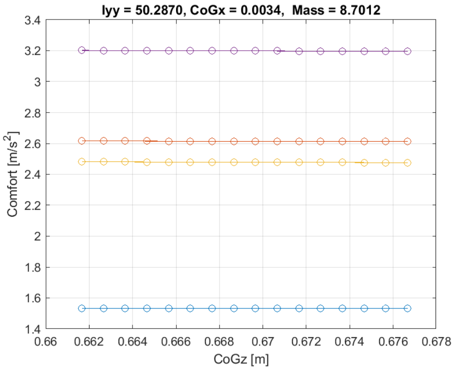

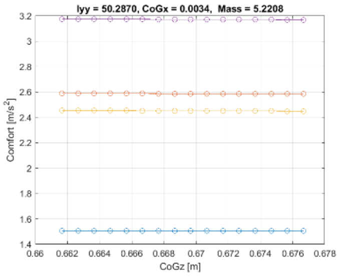

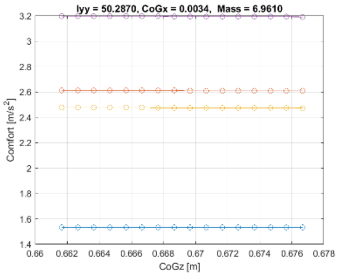

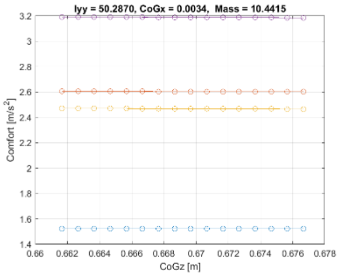

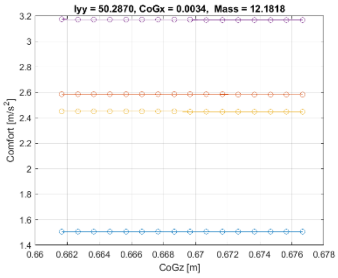

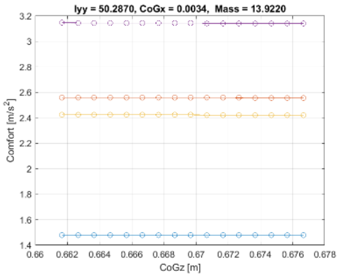

1.1.1.3 Variation with CoGx

1.1.1.3.1 Varying I_{yy}

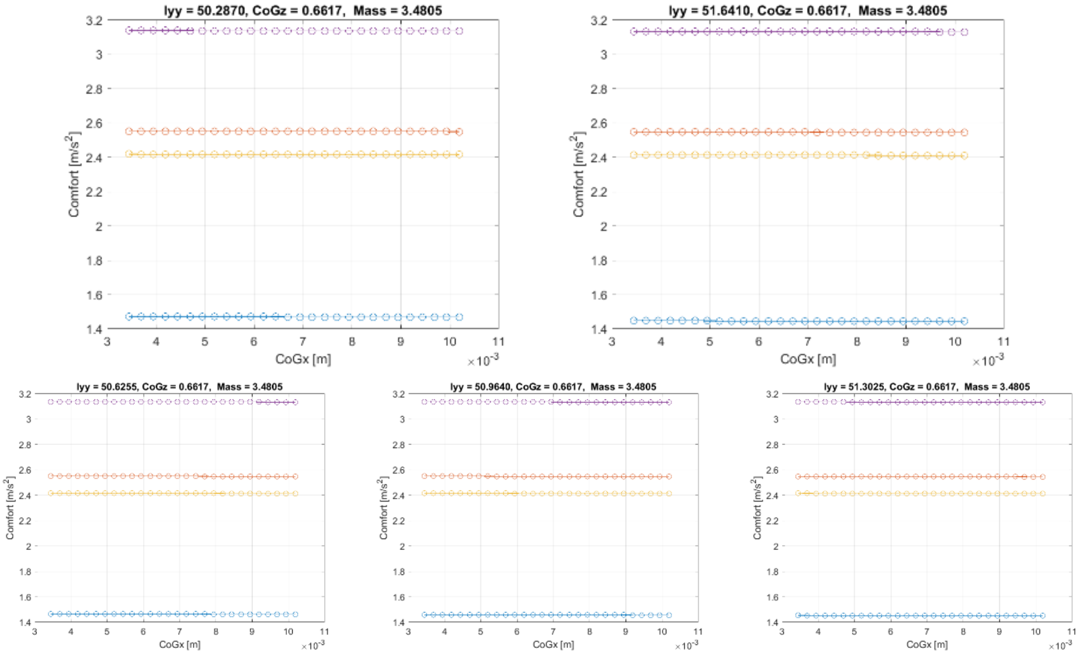

1.1.1.3.2 Varying CoGz

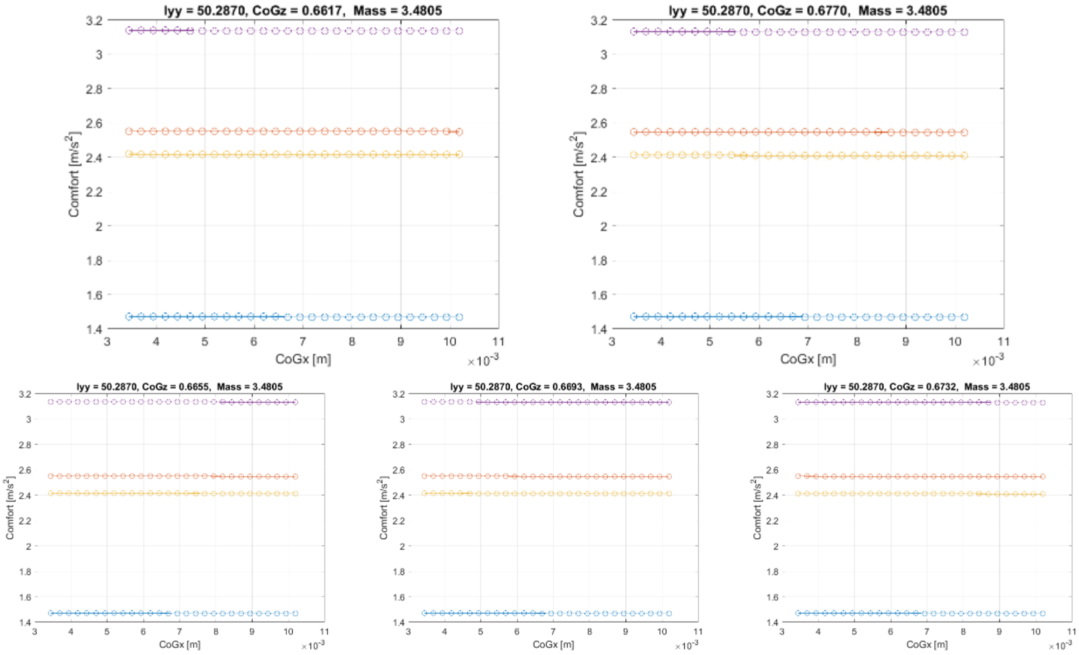

1.1.1.3.3 Varying mass

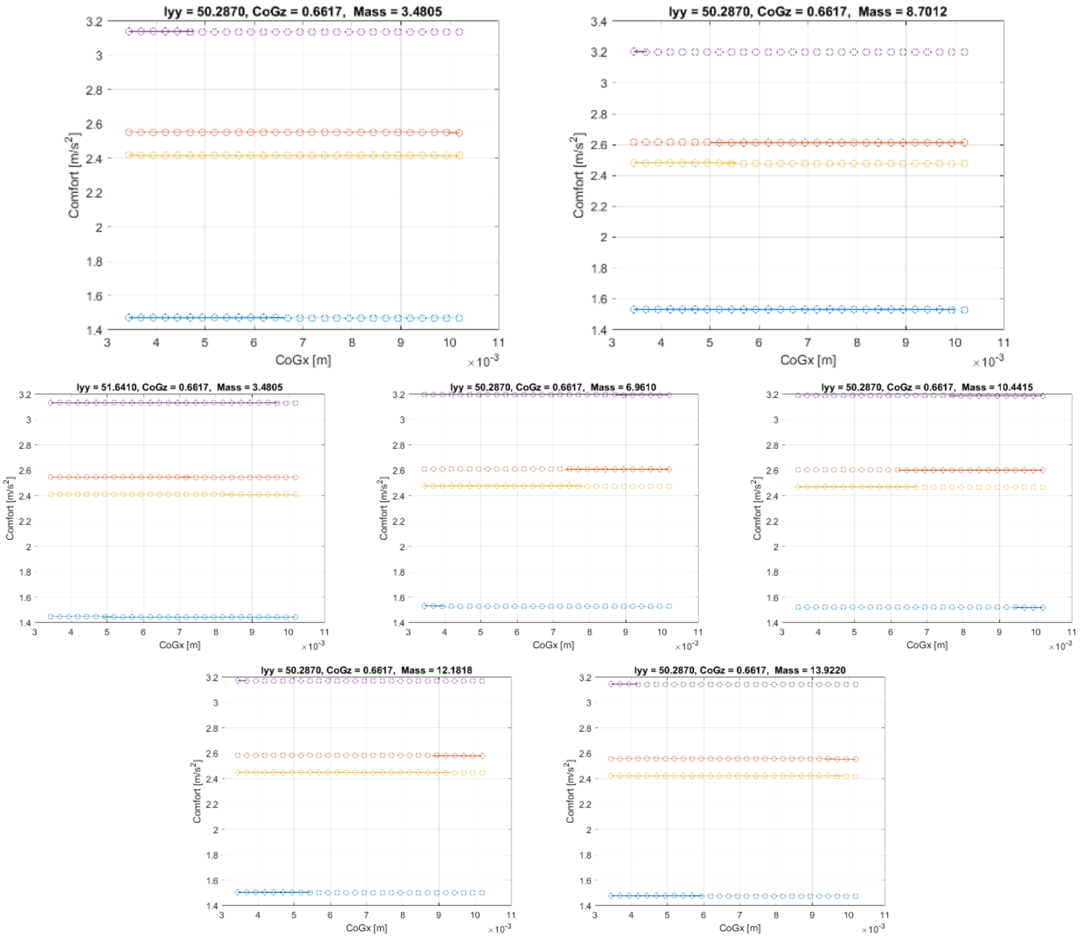

1.1.1.4 Variation with mass

1.1.1.4.1 Varying I_{yy}

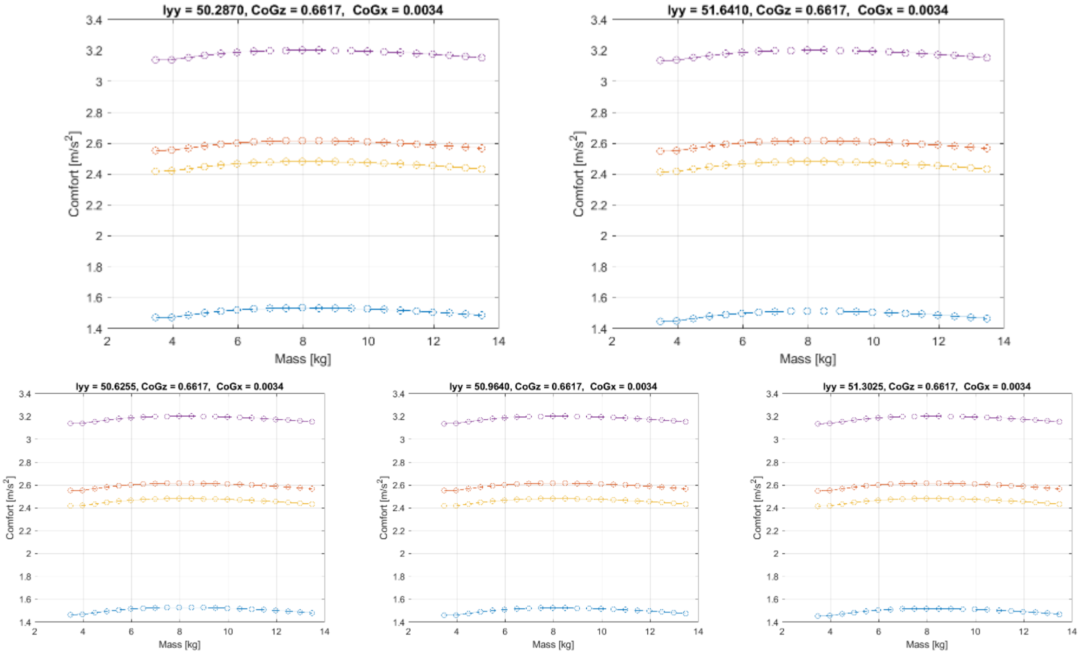

1.1.1.4.2 Varying CoGz

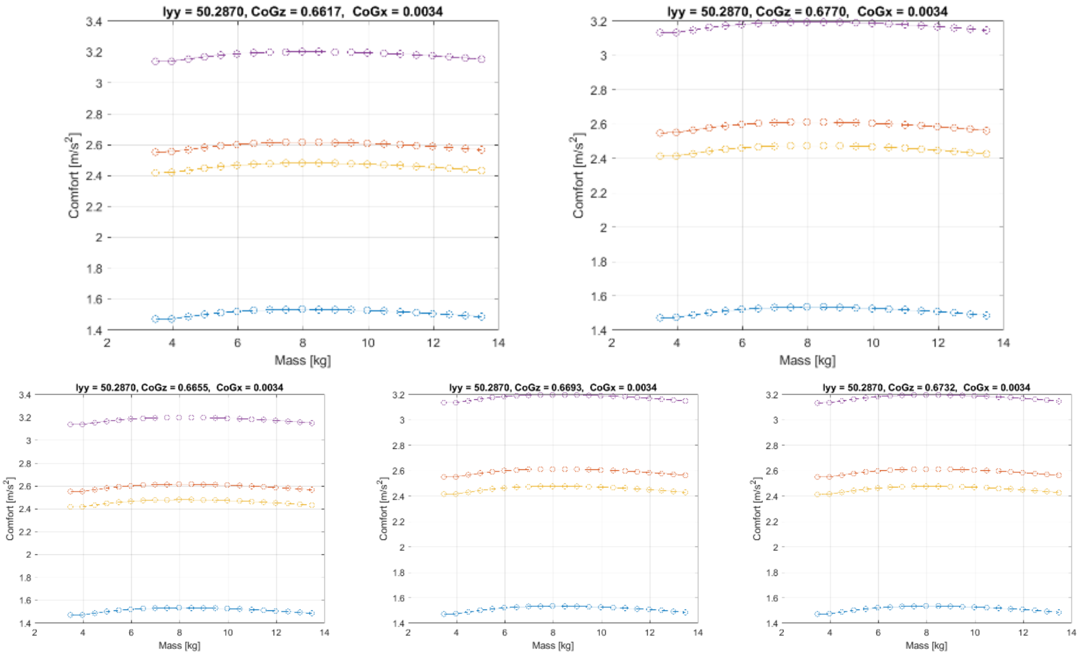

1.1.1.4.3 Varying CoGx

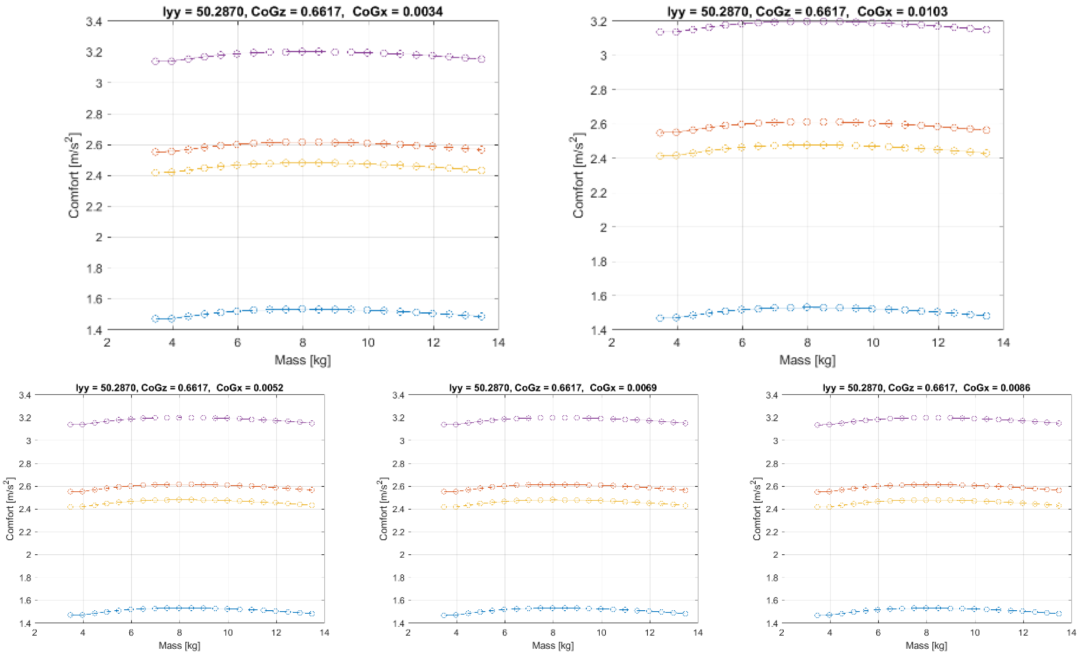

1.1.1.5 Variation with speed

1.1.1.5.1 Varying I_{yy}

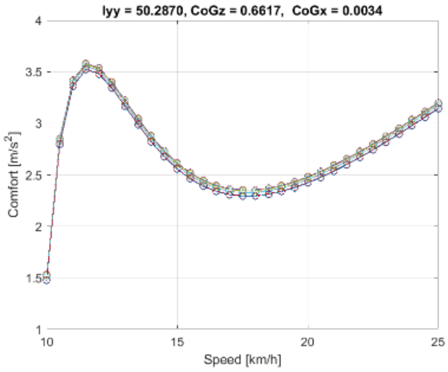

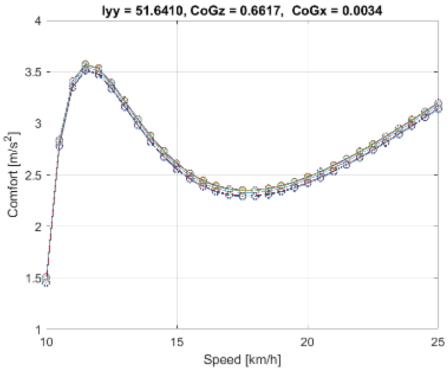

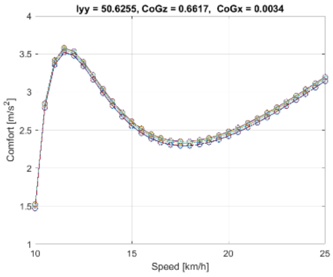

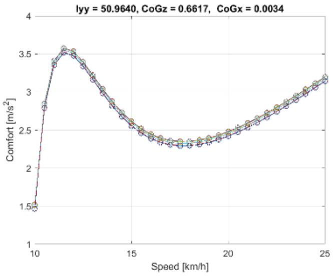

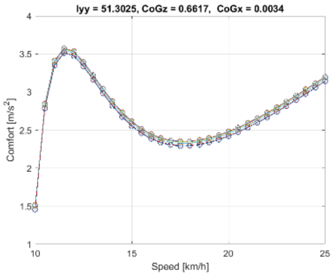

1.1.1.5.2 Varying CoGx

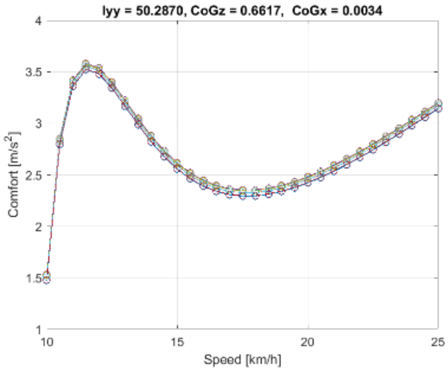

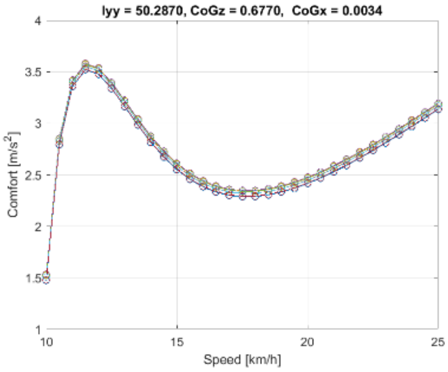

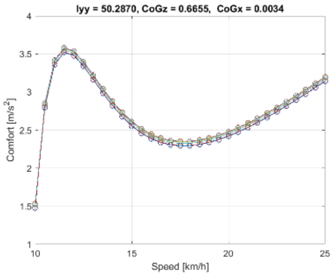

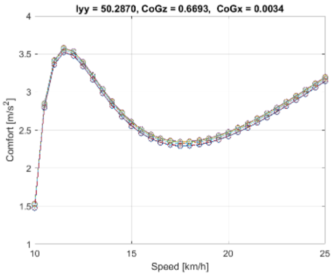

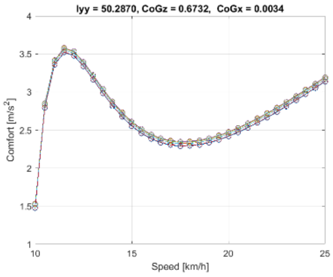

1.1.1.5.3 Varying CoGz

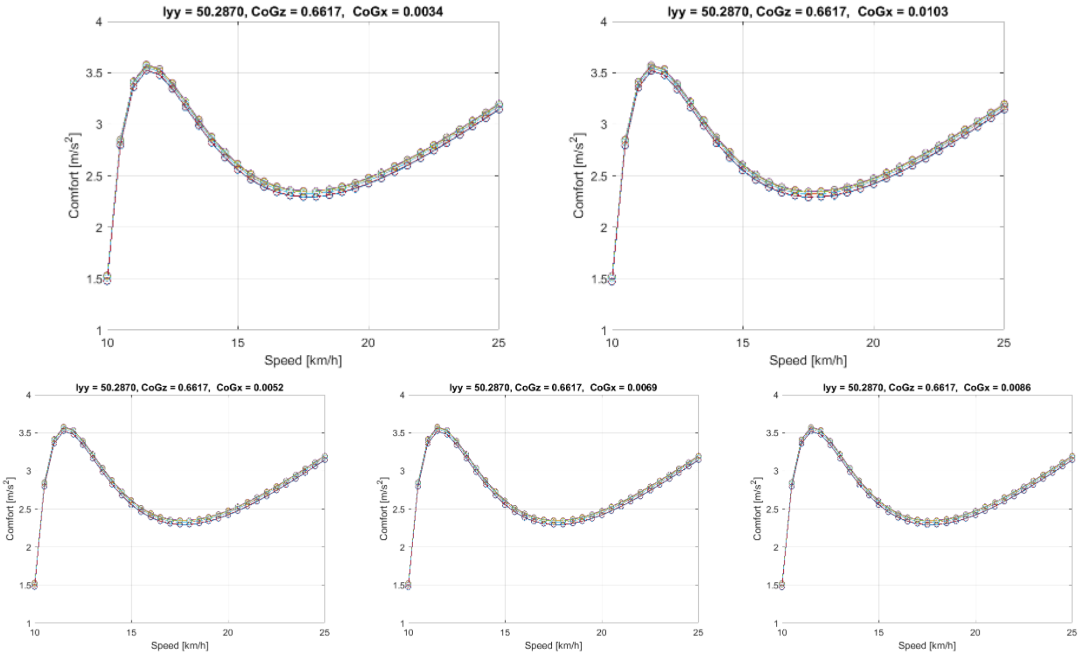

1.1.2 Raw data

1.1.2.1 Variation with I_{yy}

1.1.2.1.1 Varying CoGz

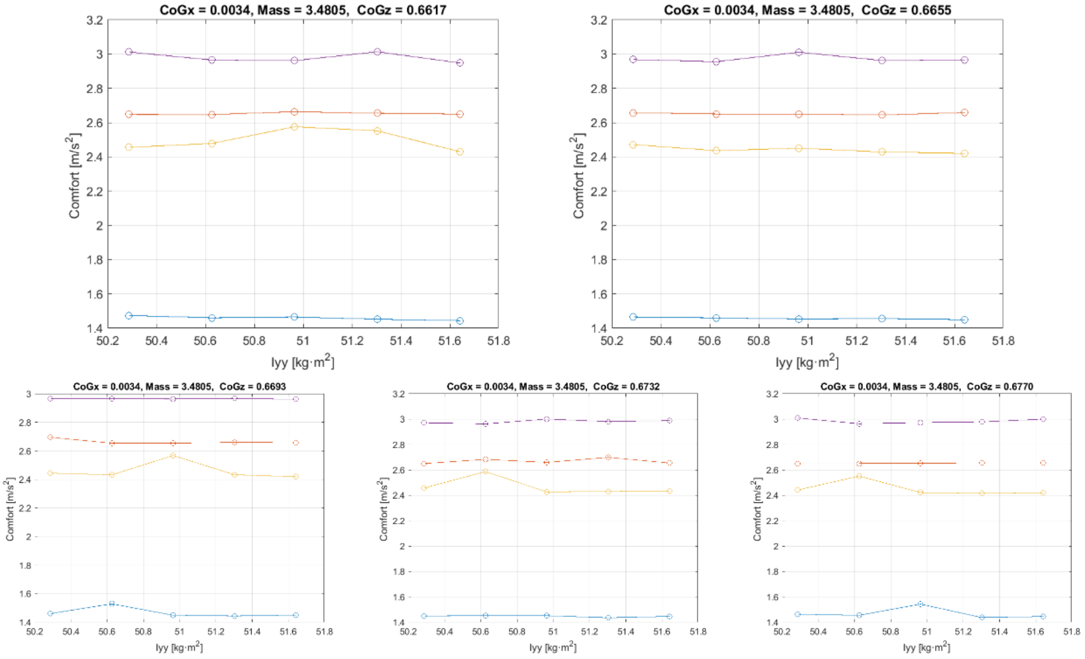

1.1.2.1.2 Varying CoGx

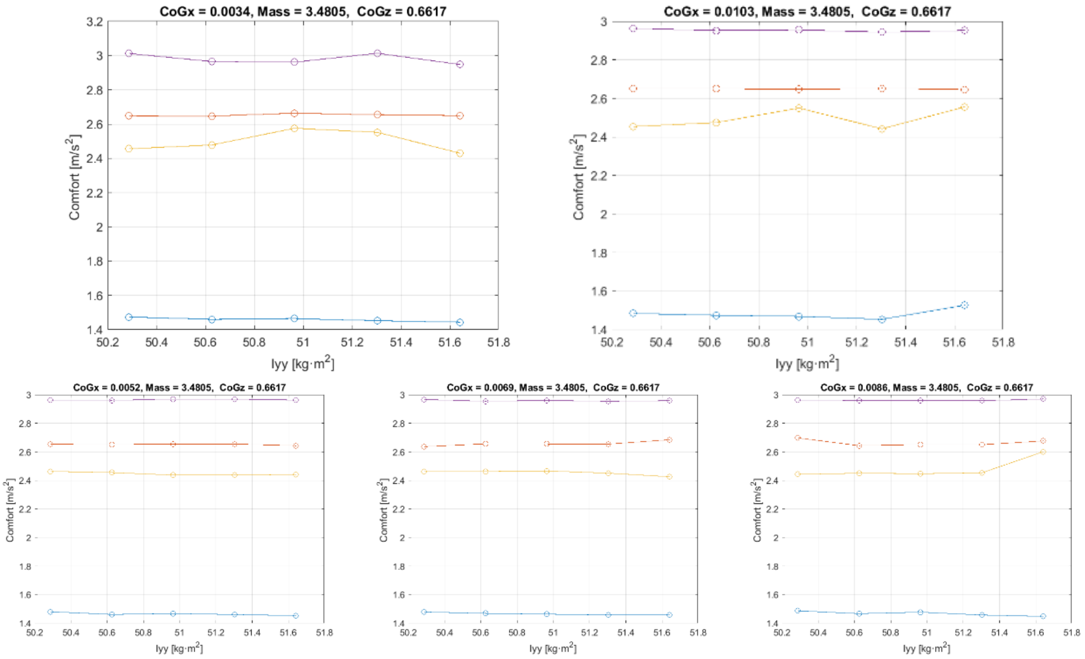

1.1.2.1.3 Varying mass

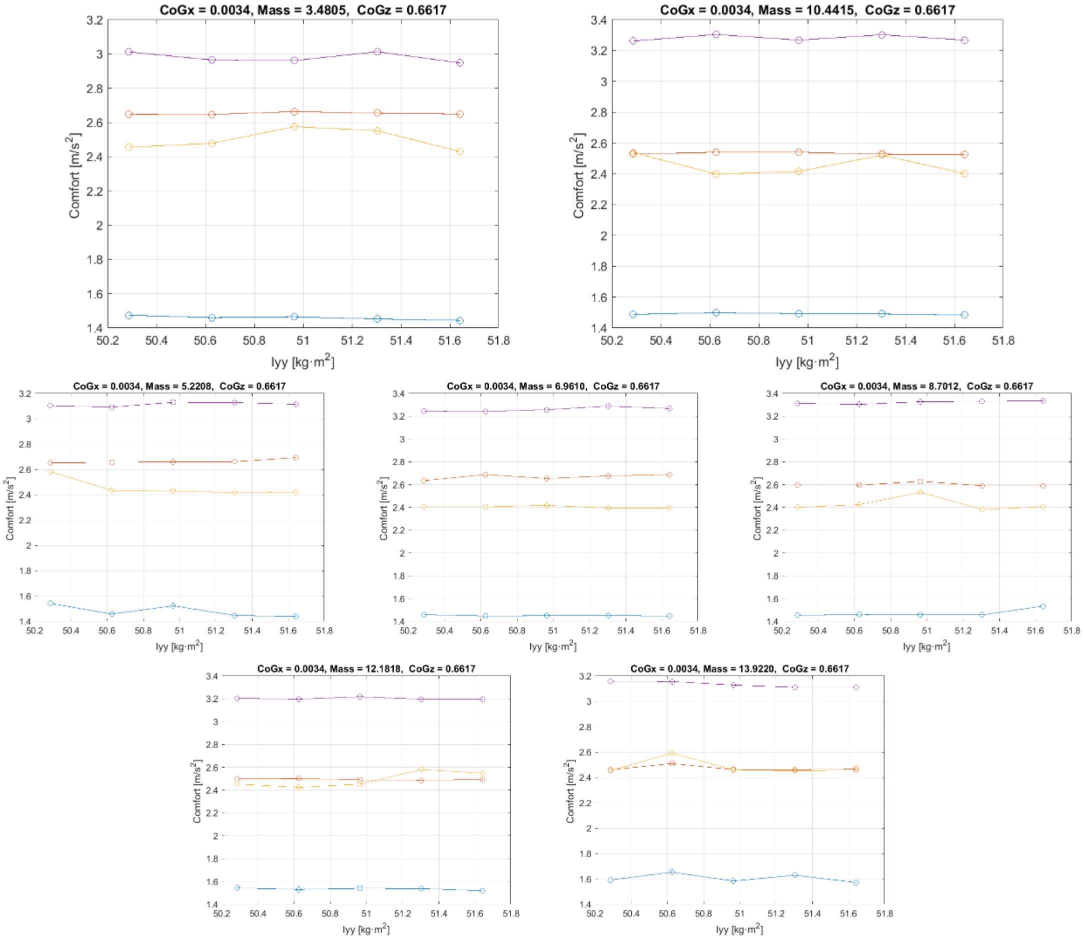

1.1.2.2 Variation with CoGz

1.1.2.2.1 Varying I_{yy}

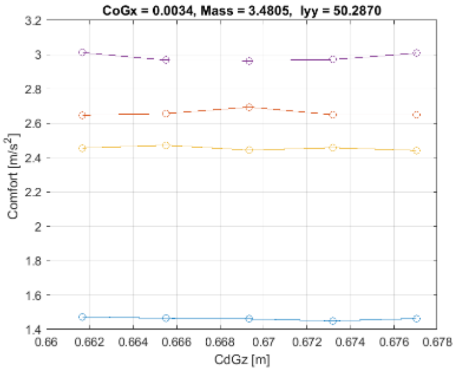

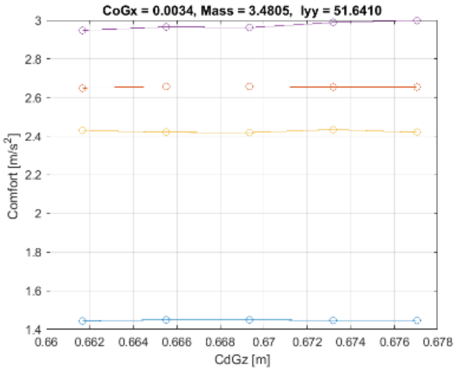

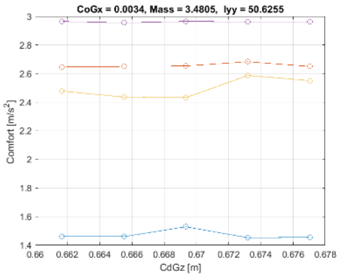

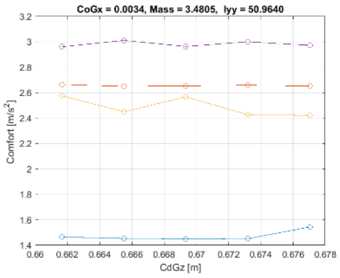

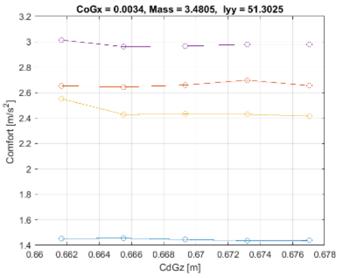

1.1.2.2.2 Varying CoGx

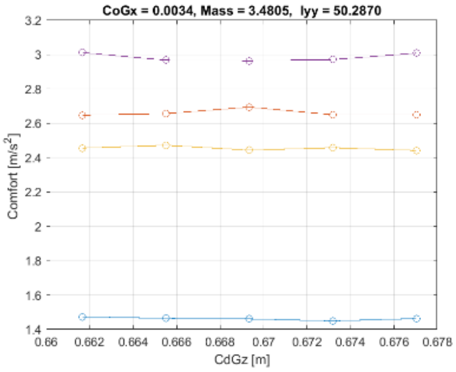

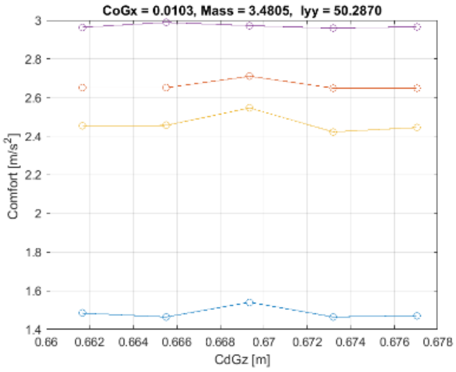

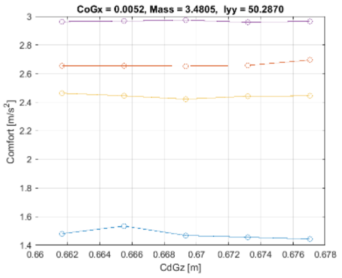

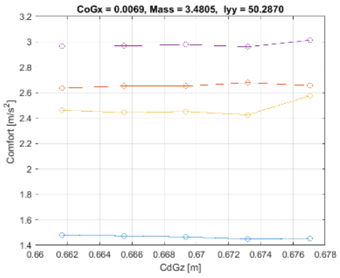

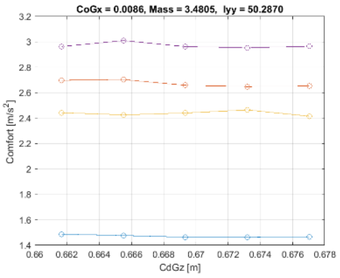

1.1.2.2.3 Varying mass

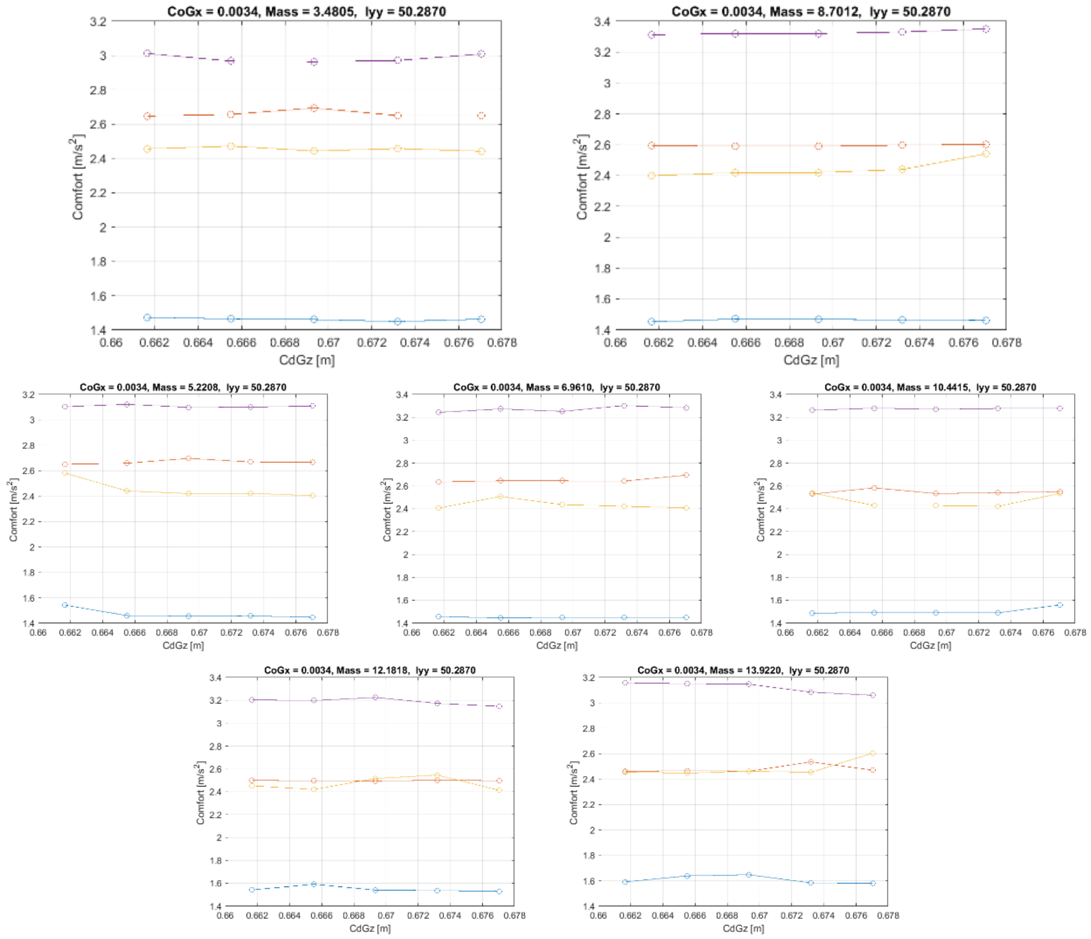

1.1.2.3 Variation with CoGx

1.1.2.3.1 Varying I_{yy}

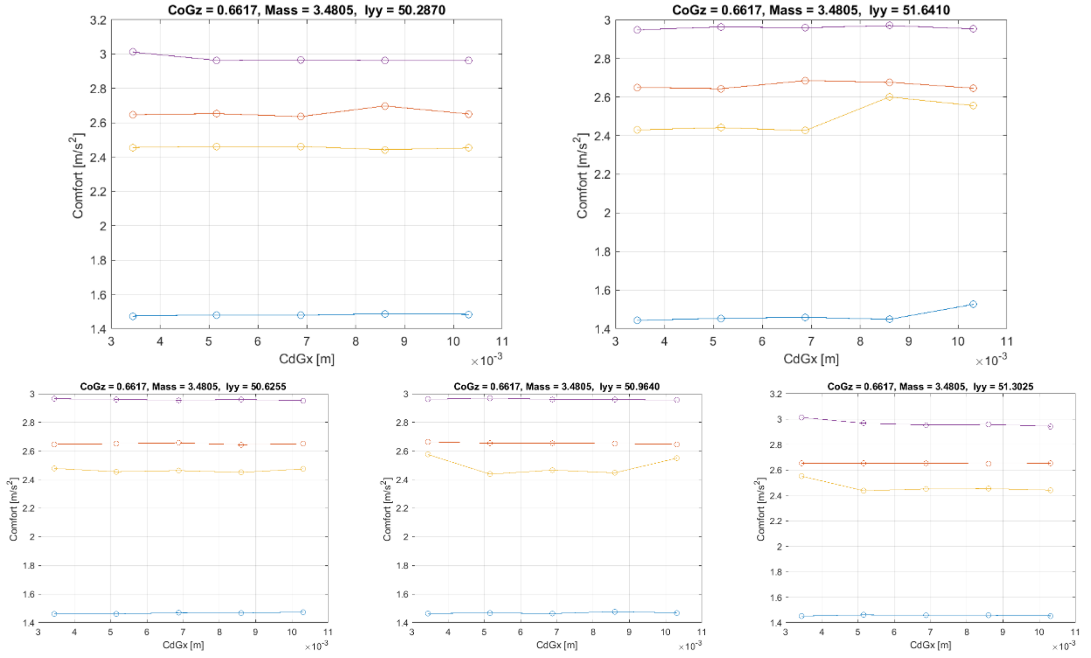

1.1.2.3.2 Varying CoGz

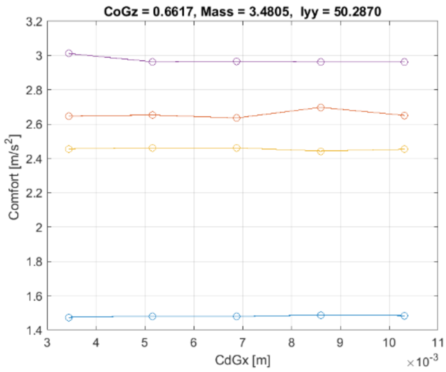

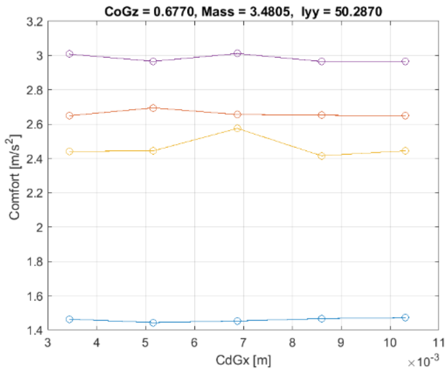

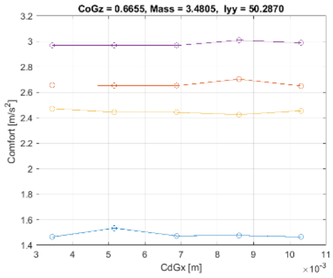

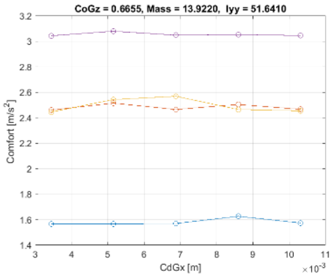

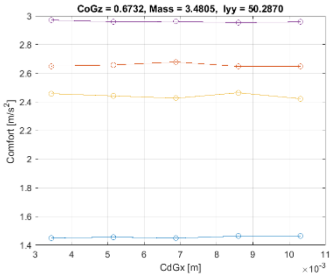

1.1.2.3.3 Varying mass

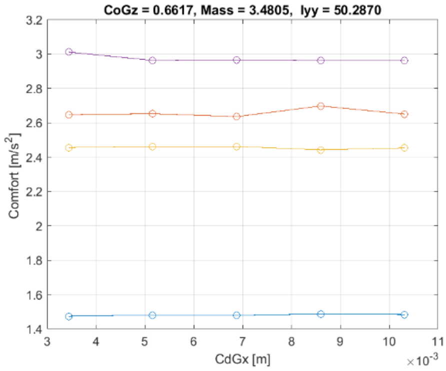

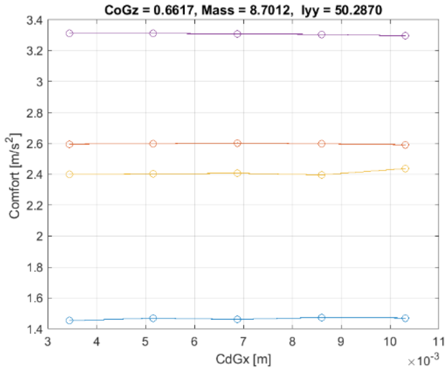

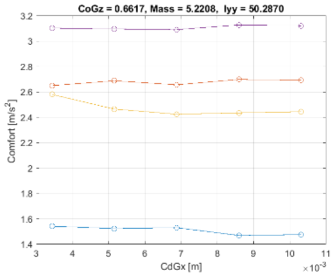

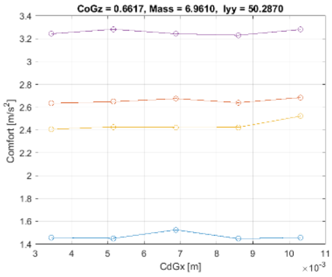

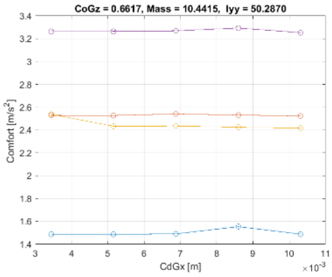

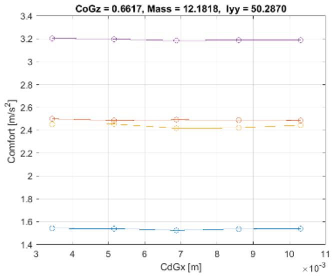

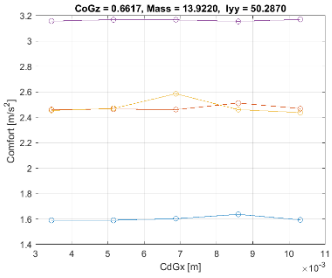

1.1.2.4 Variation with mass

1.1.2.4.1 Varying I_{yy}

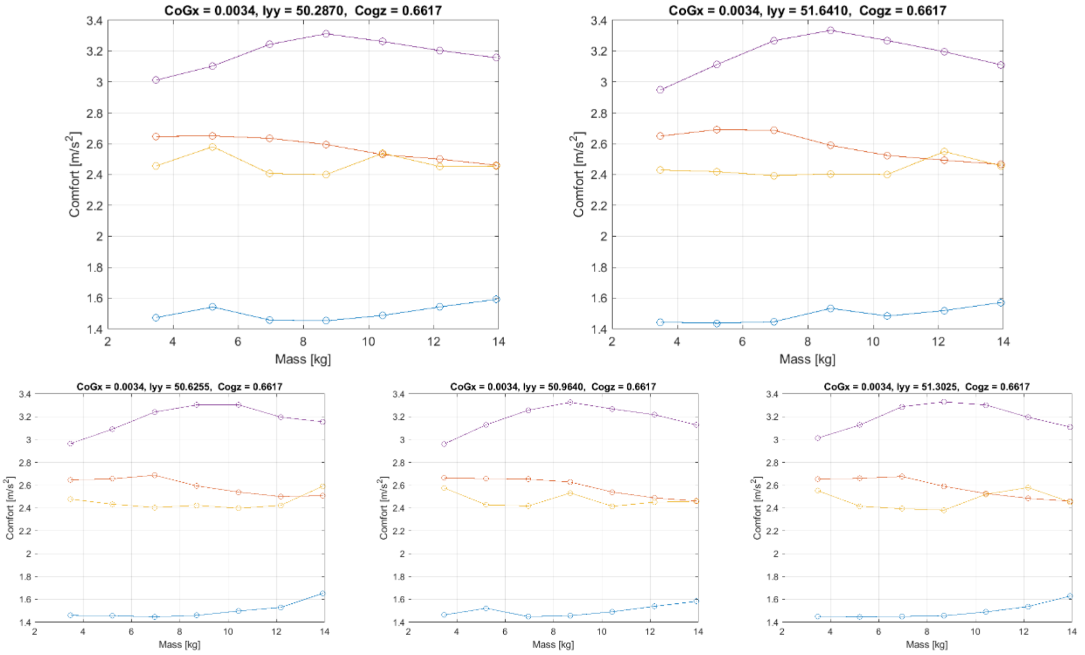

1.1.2.4.2 Varying CoGz

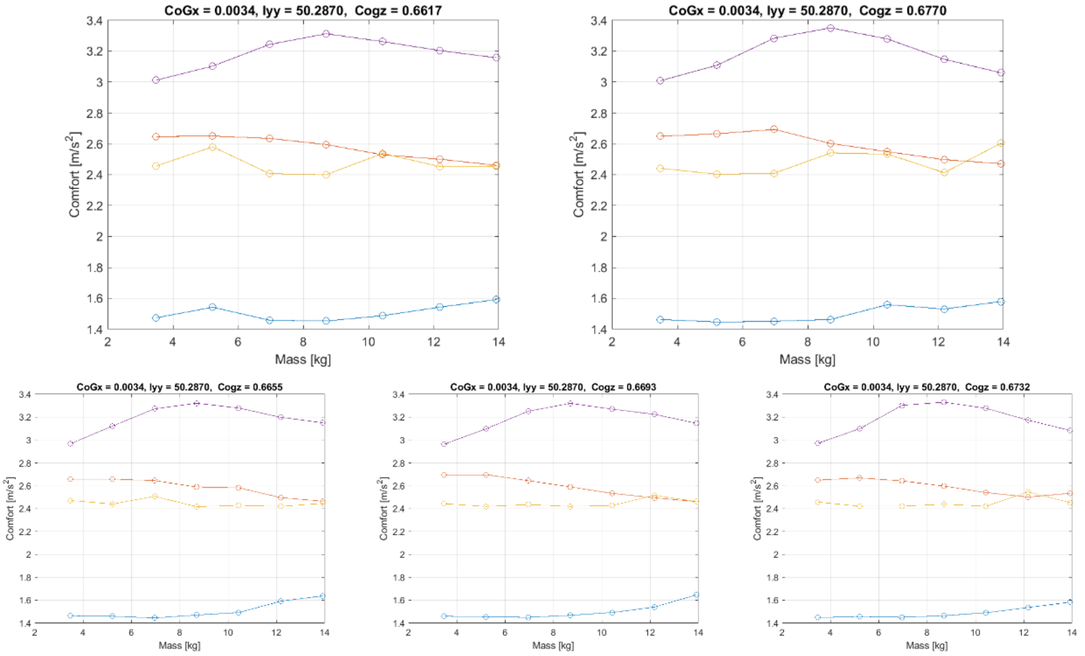

1.1.2.4.3 Varying CoGx

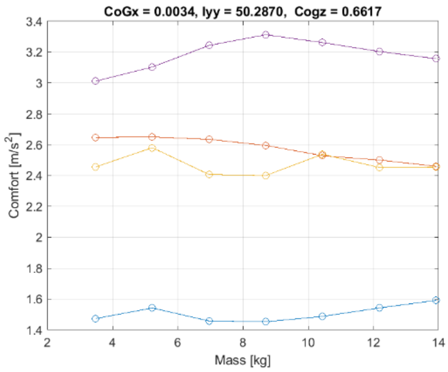

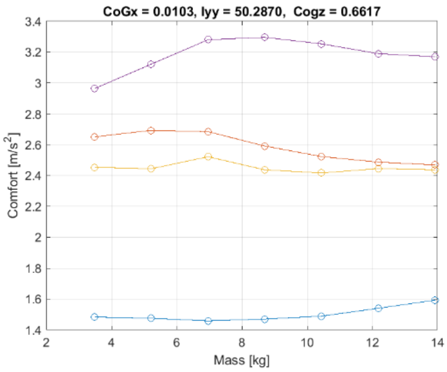

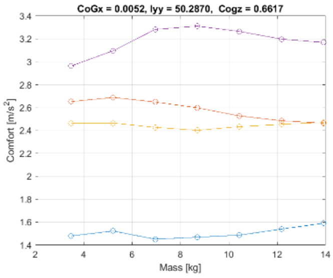

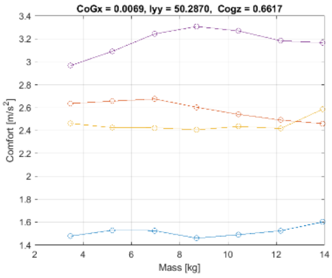

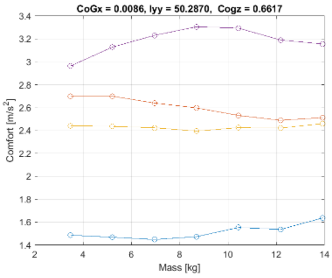

1.1.2.5 Variation with speed

1.1.2.5.1 Varying I_{yy}

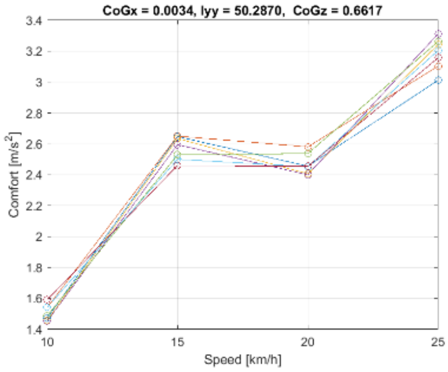

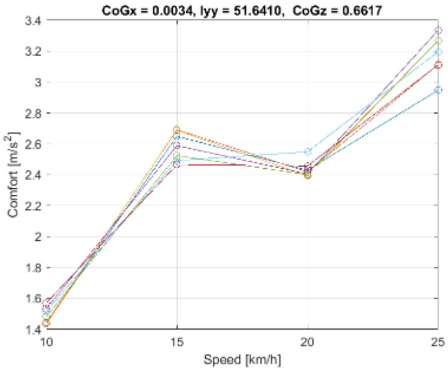

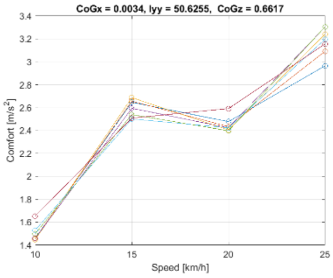

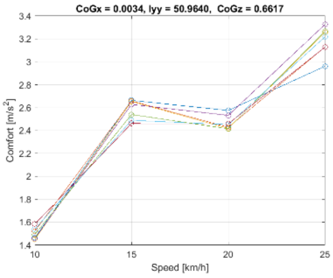

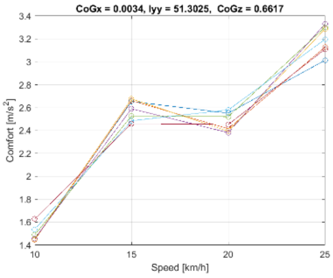

1.1.2.5.2 Varying CoGx

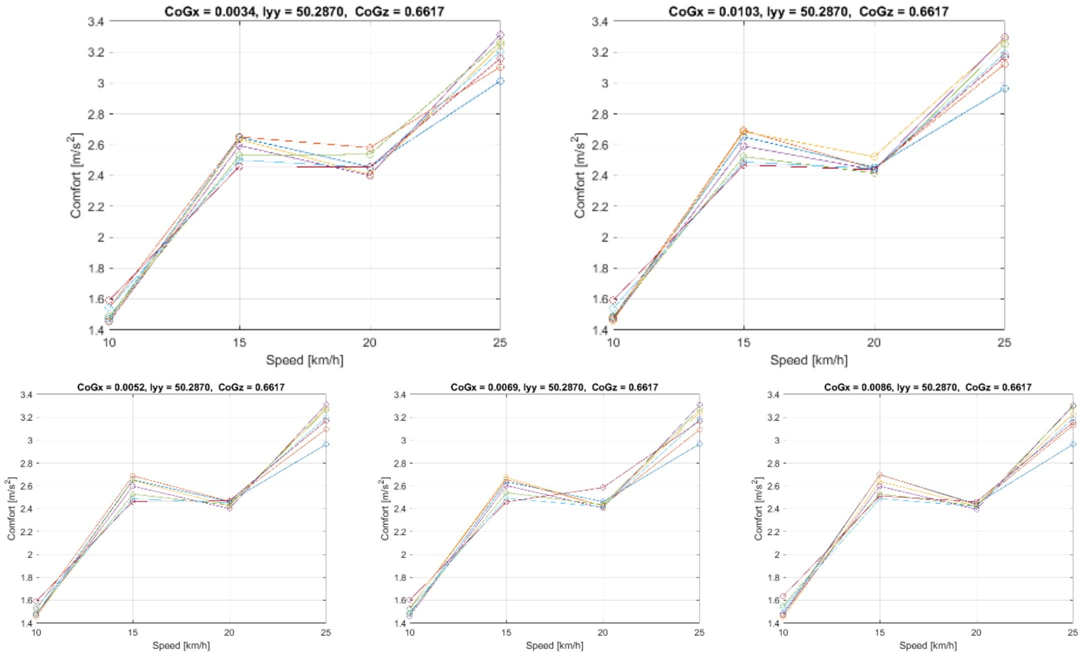

1.1.2.5.3 Varying CoGz

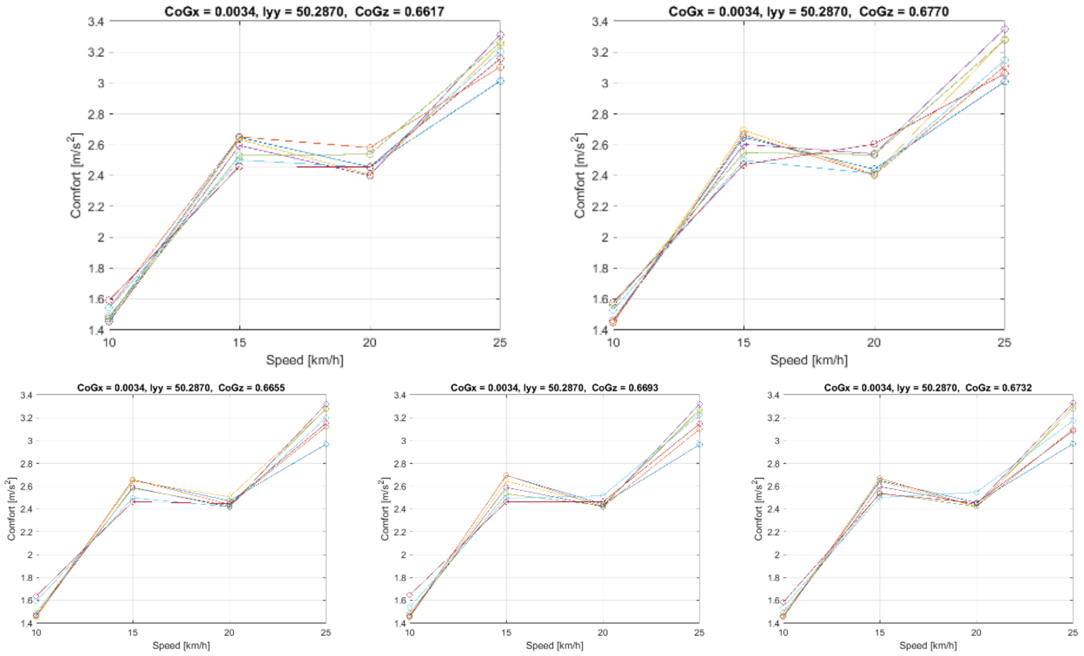

1.2 Contour Maps

1.2.1 Statistical data

1.2.1.1 Variation with speed and I_{yy}

1.2.1.1.1 Varying CoGz

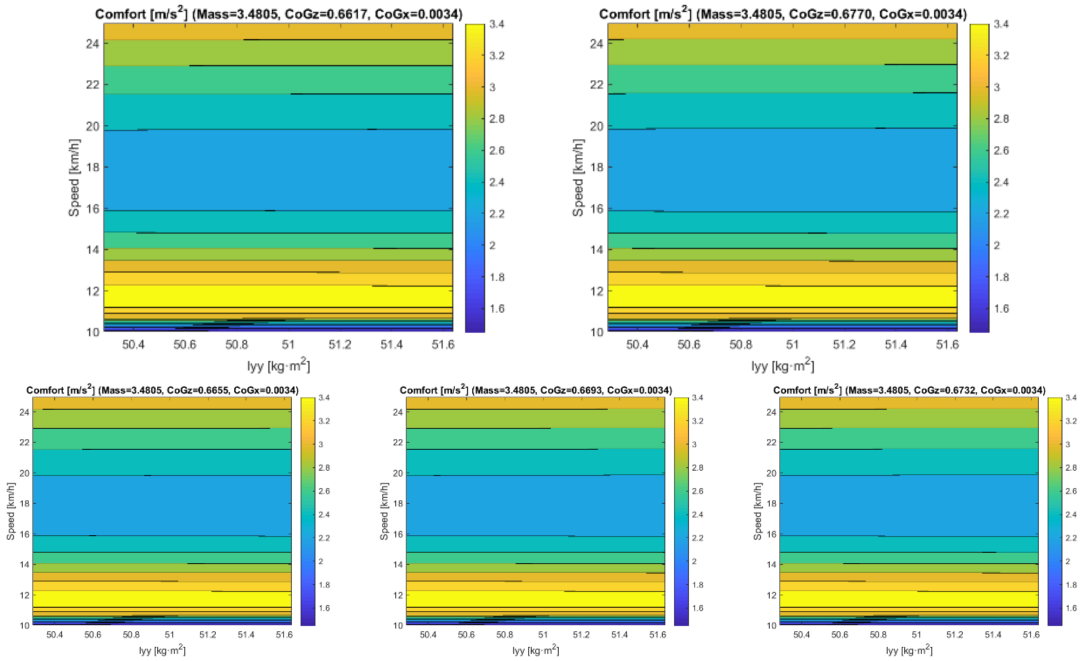

1.2.1.1.2 Varying CoGx

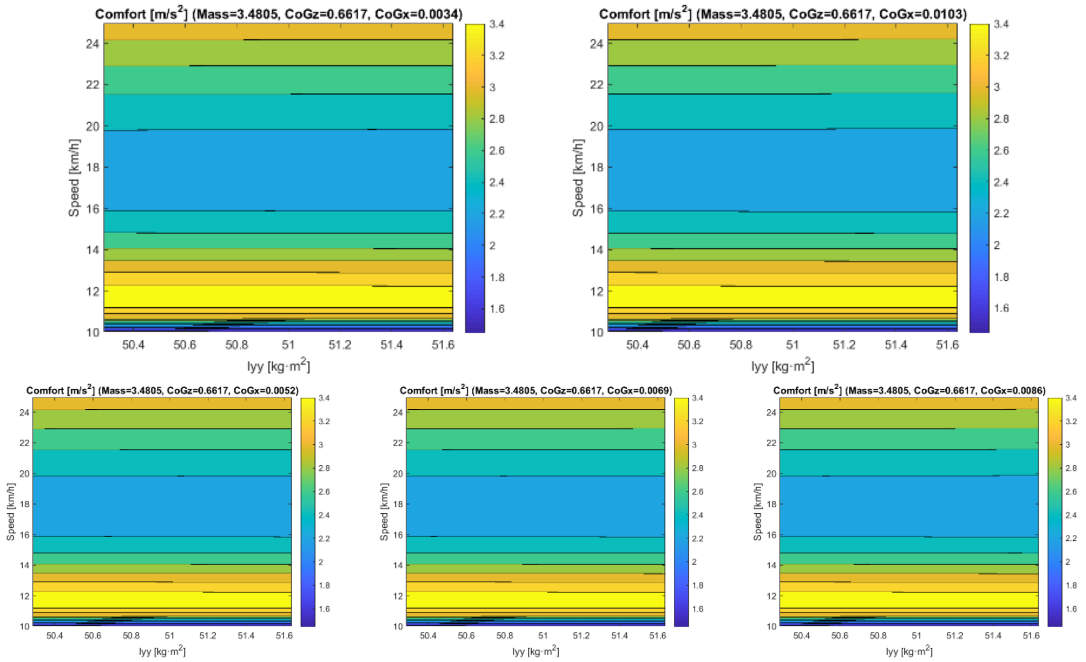

1.2.1.1.3 Varying mass

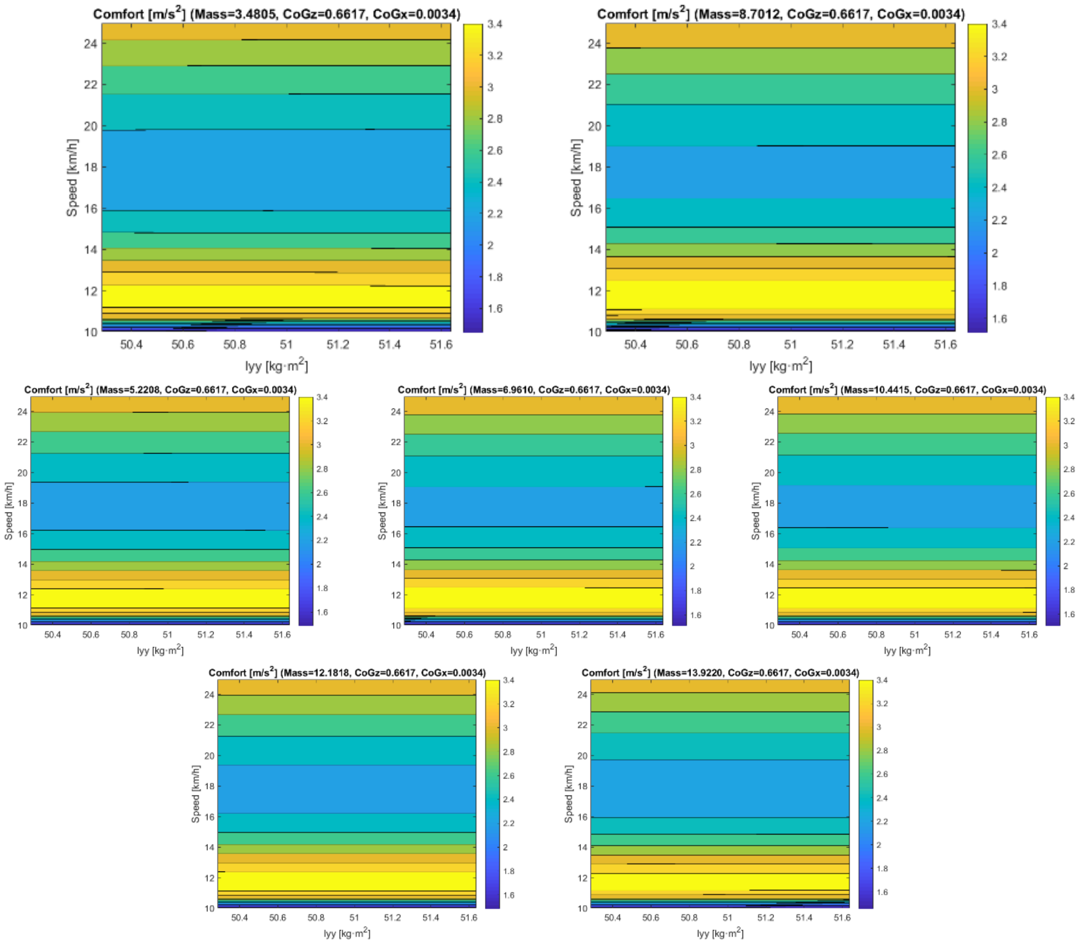

1.2.1.2 Variation with speed and CoGz

1.2.1.2.1 Varying I_{yy}

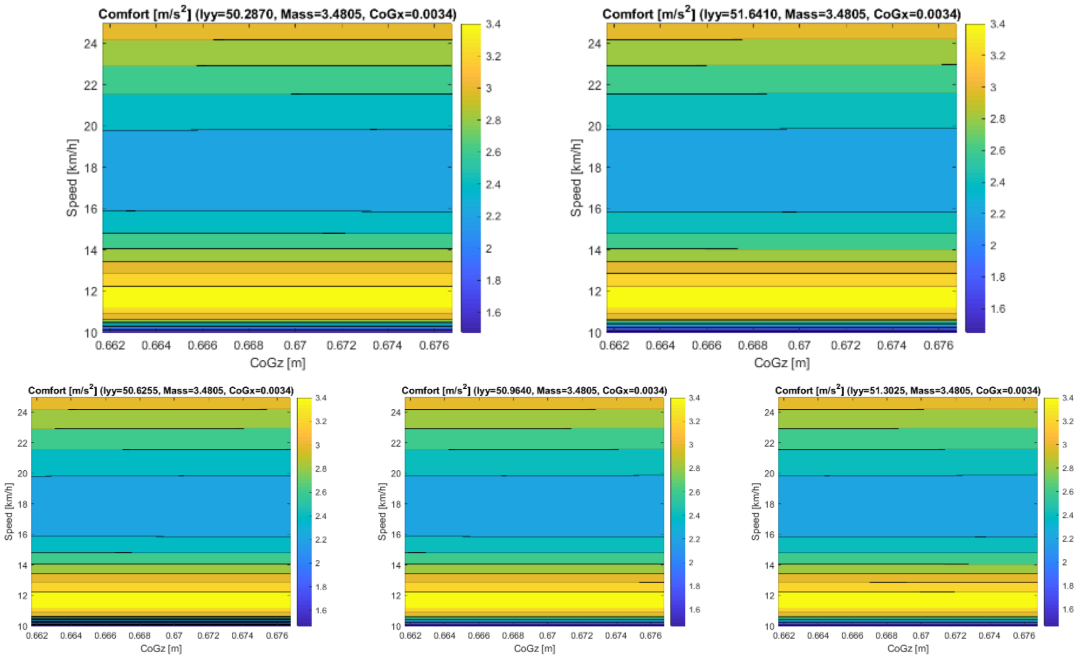

1.2.1.2.2 Varying CoGx

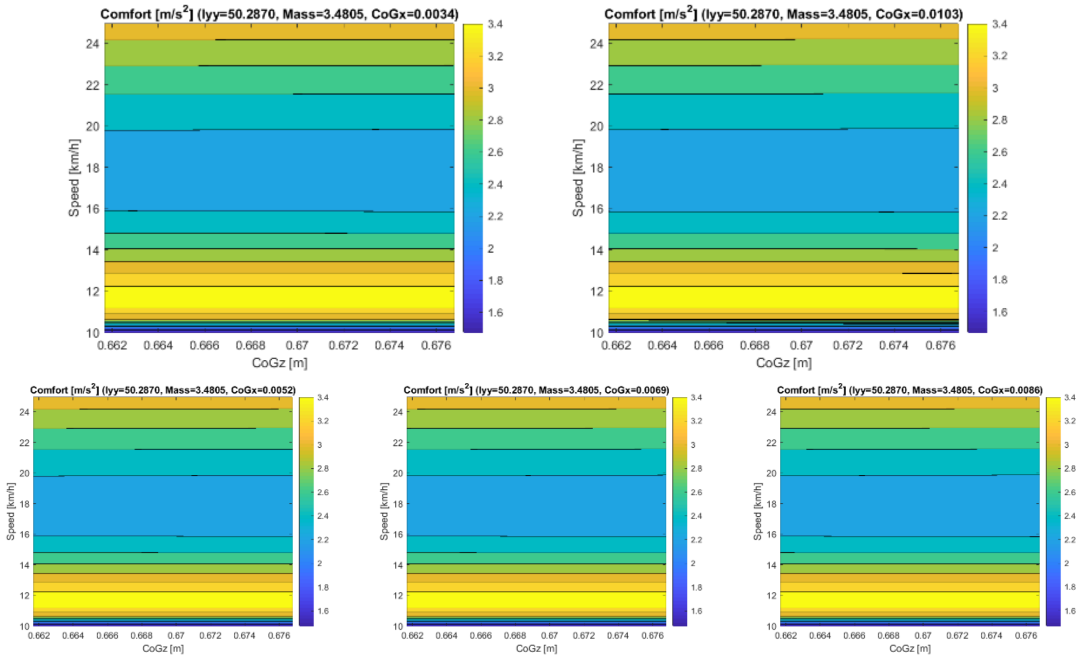

1.2.1.2.3 Varying mass

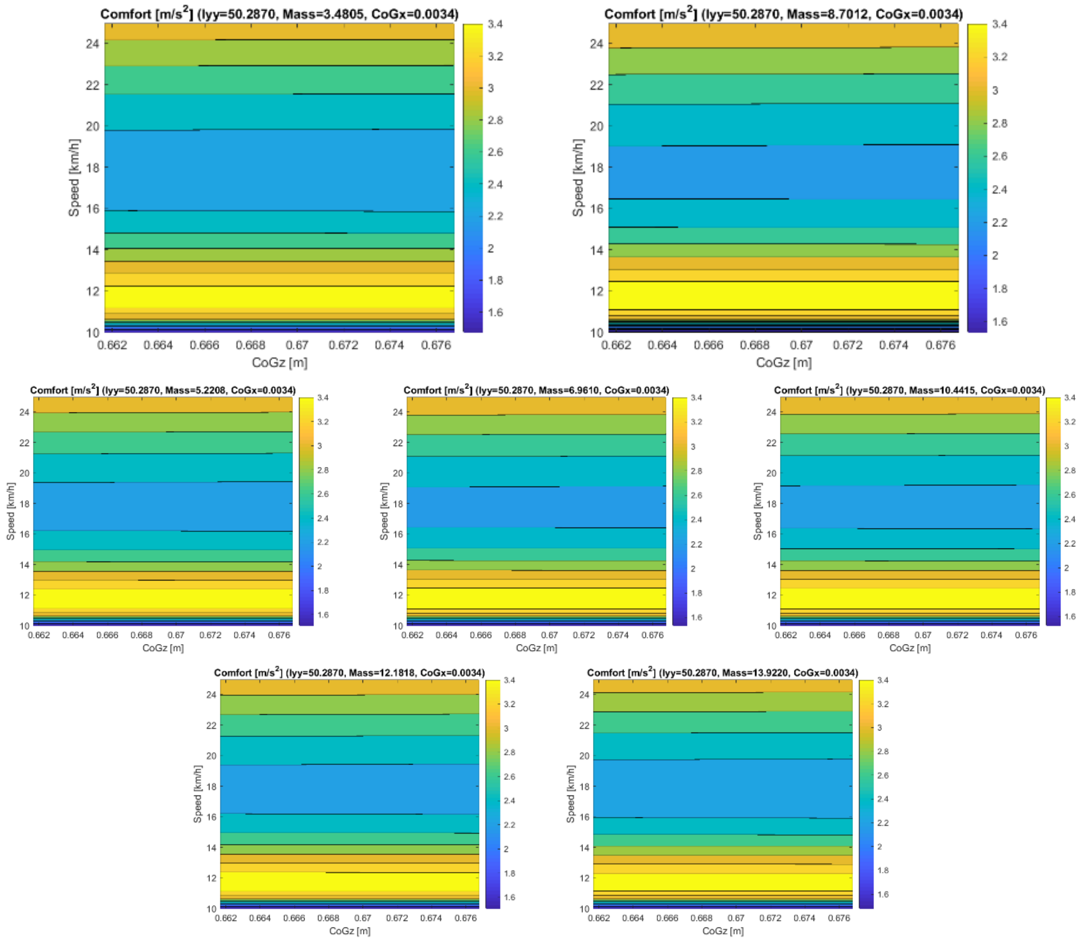

1.2.1.3 *Variation with speed and CoGx*

1.2.1.3.1 Varying I_{yy}

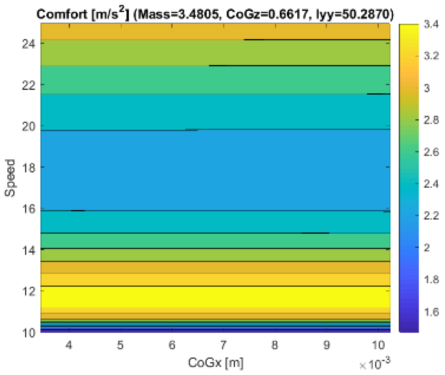

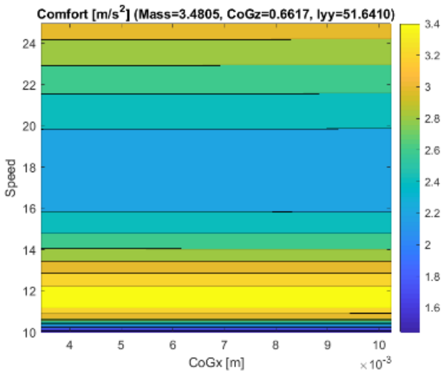

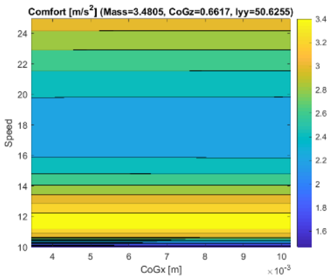

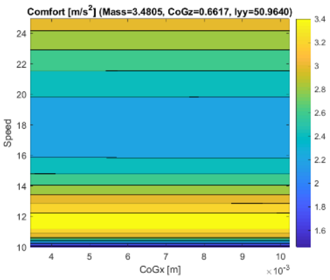

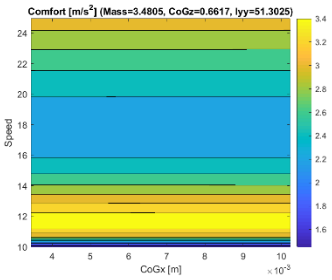

1.2.1.3.2 Varying CoGz

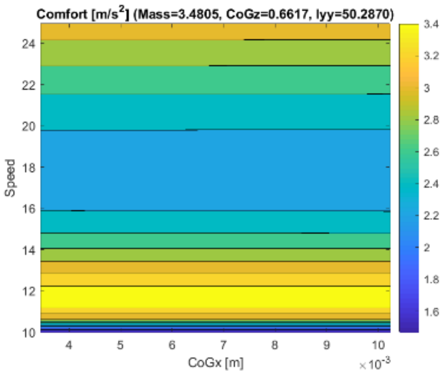

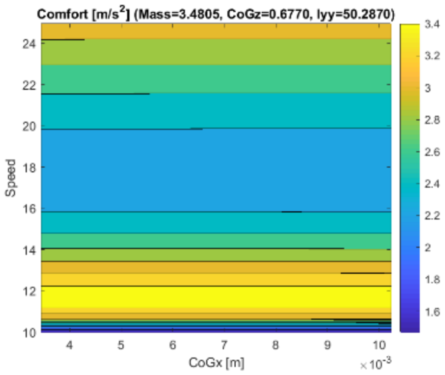

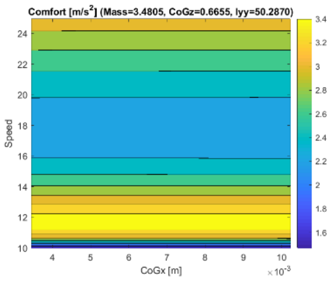

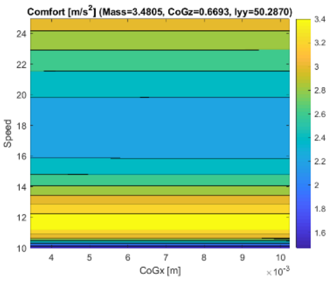

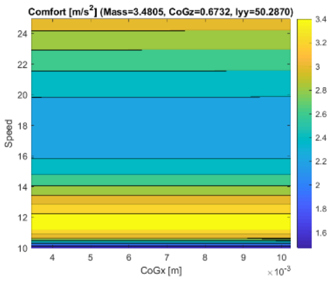

1.2.1.3.3 Varying mass

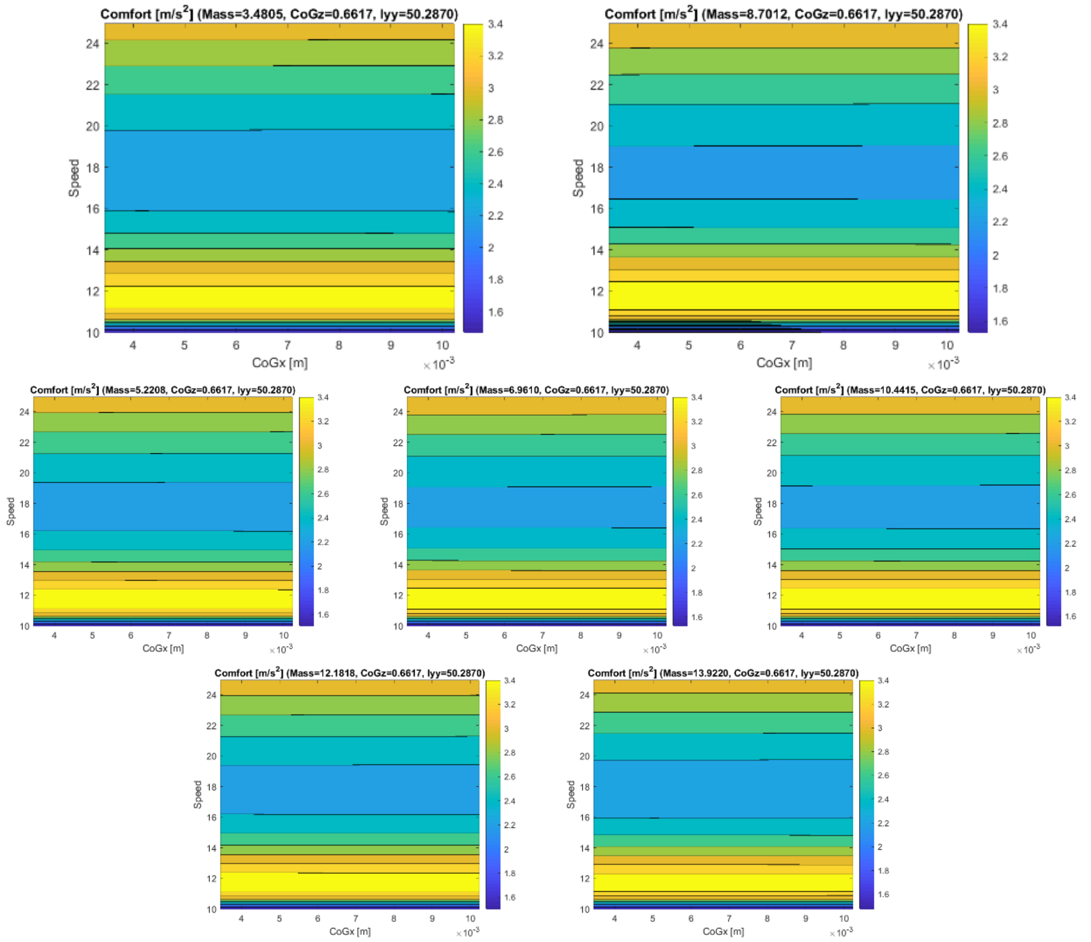

1.2.1.4 Variation with speed and mass

1.2.1.4.1 Varying I_{yy}

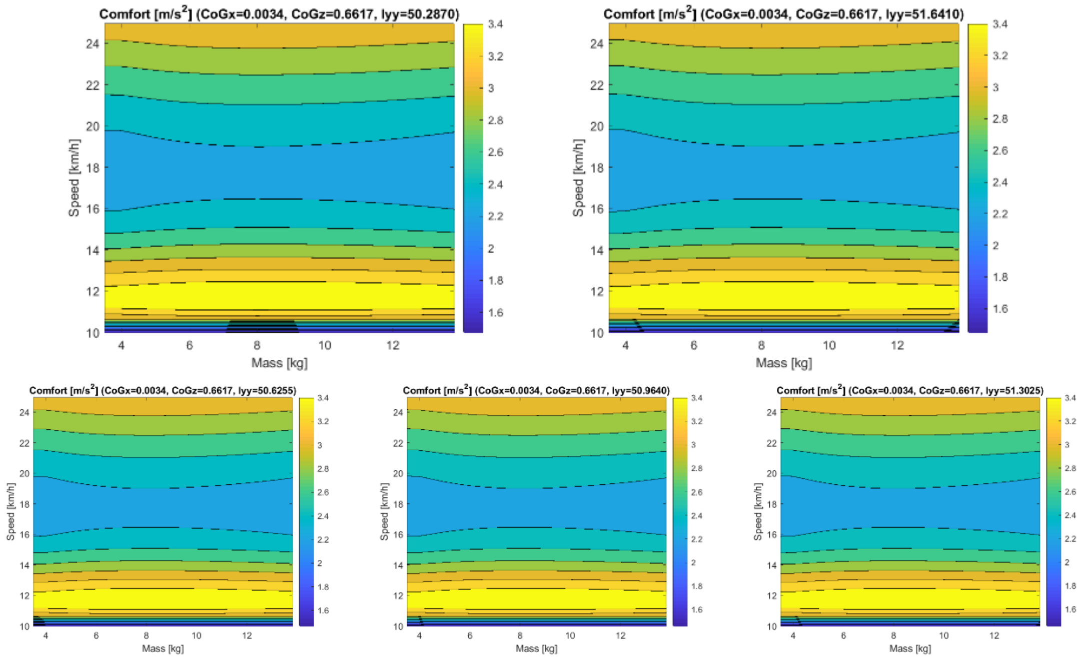

1.2.1.4.2 Varying CoGz

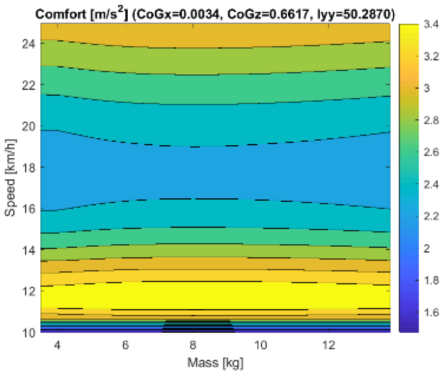

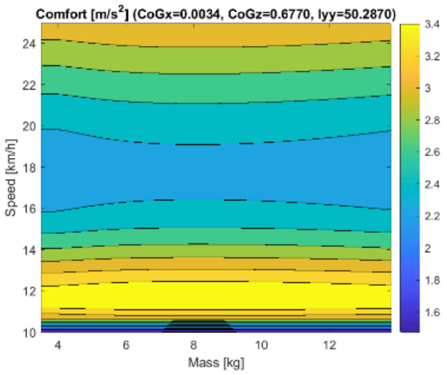

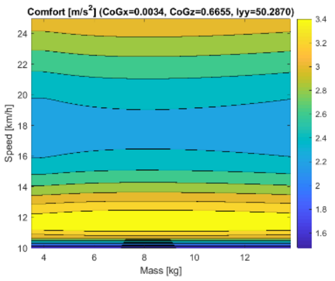

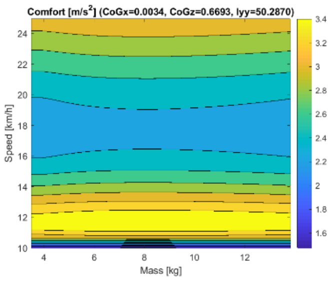

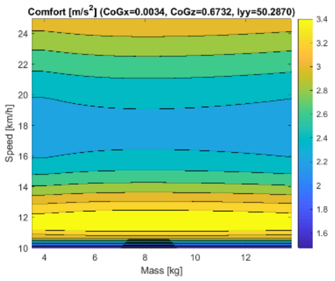

1.2.1.4.3 Varying CoGx

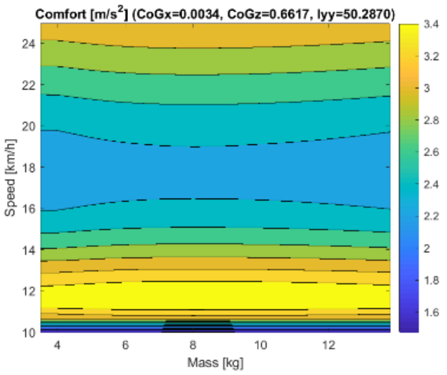

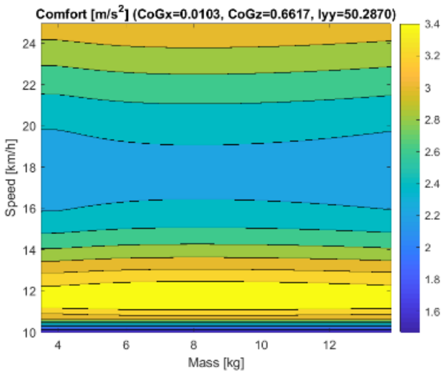

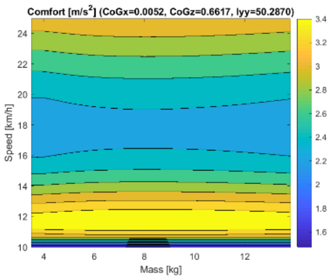

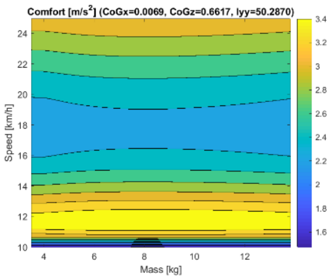

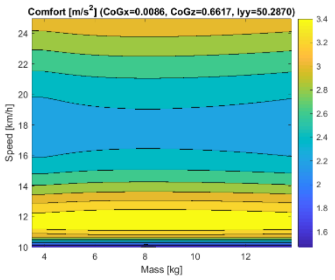

1.2.1.5 *Variation with mass and I_{yy}*

1.2.1.5.1 Varying CoGz

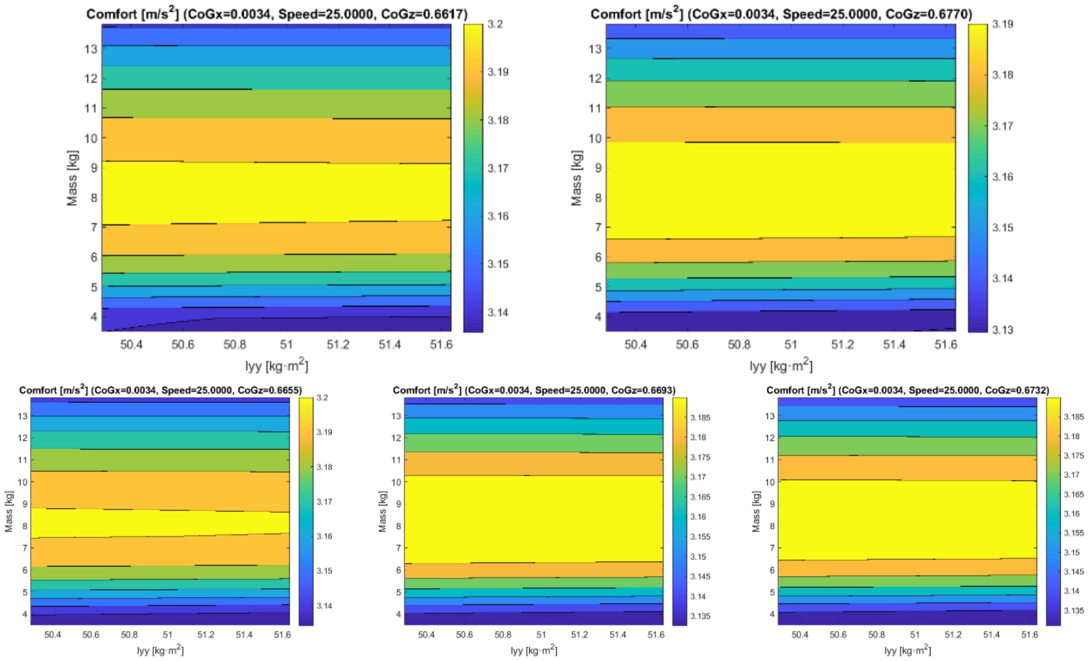

1.2.1.5.2 Varying CoGx

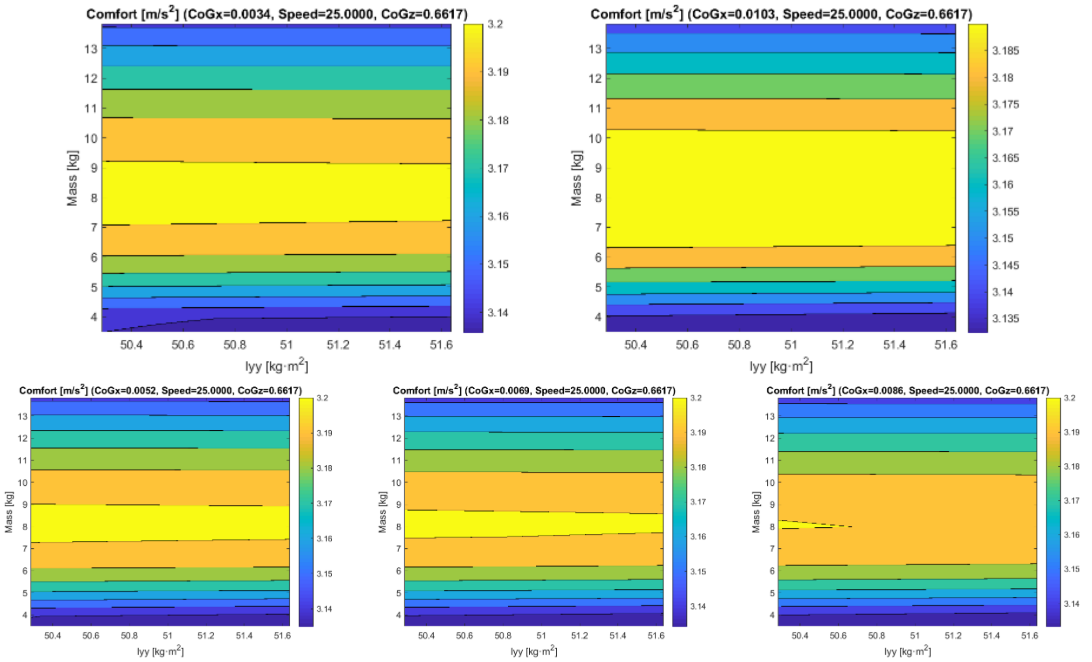

1.2.1.5.3 Varying speed

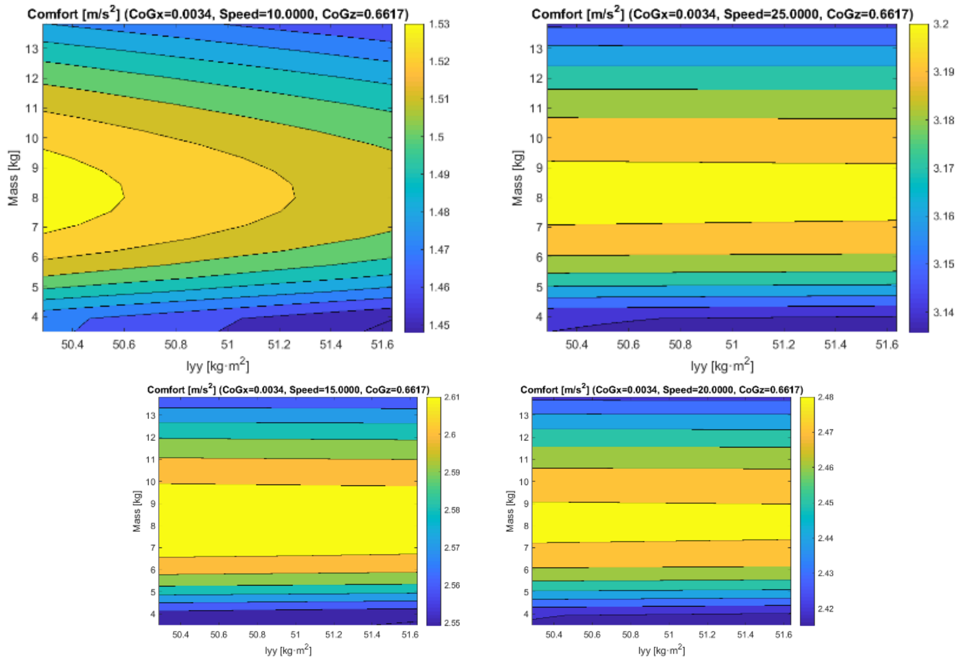

1.2.1.6 Variation with mass and CoGz

1.2.1.6.1 Varying I_{yy}

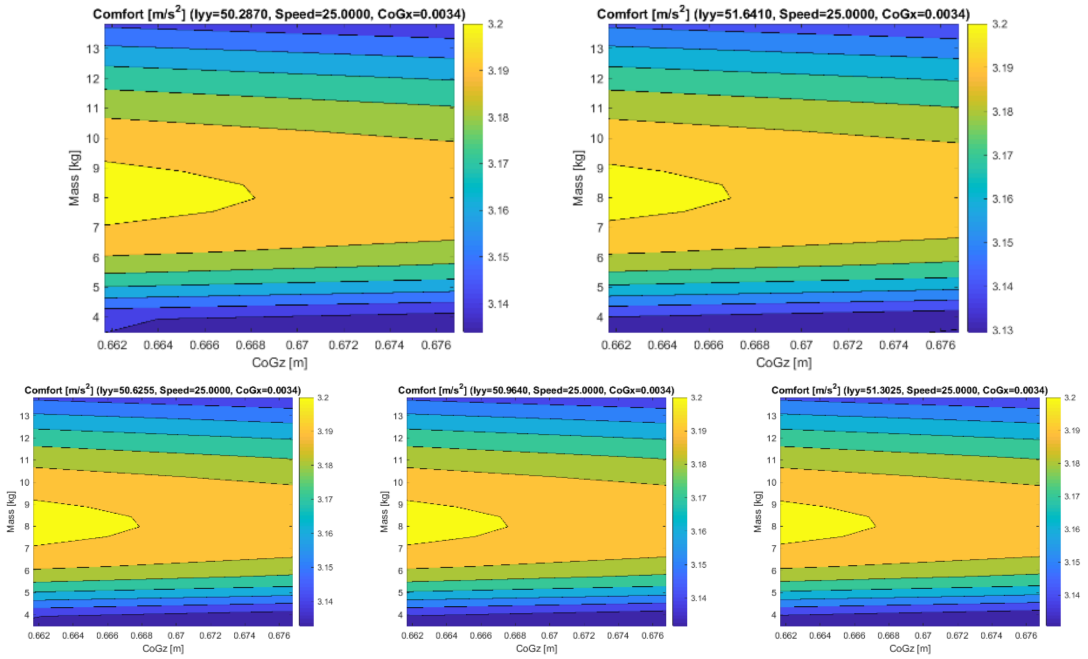

1.2.1.6.2 Varying CoGx

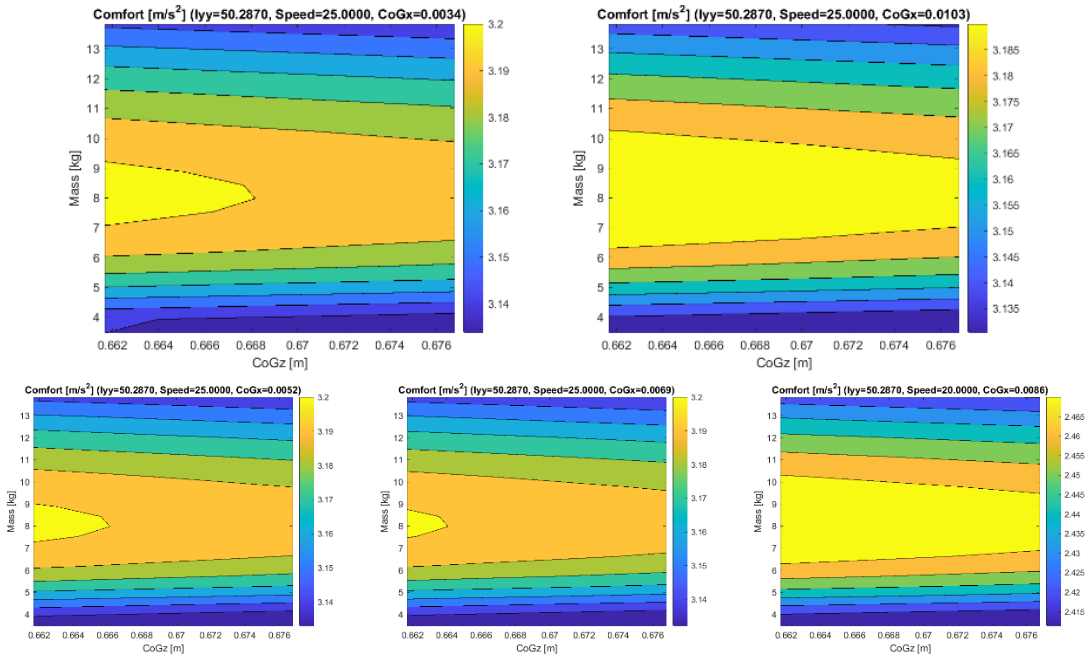

1.2.1.6.3 Varying speed

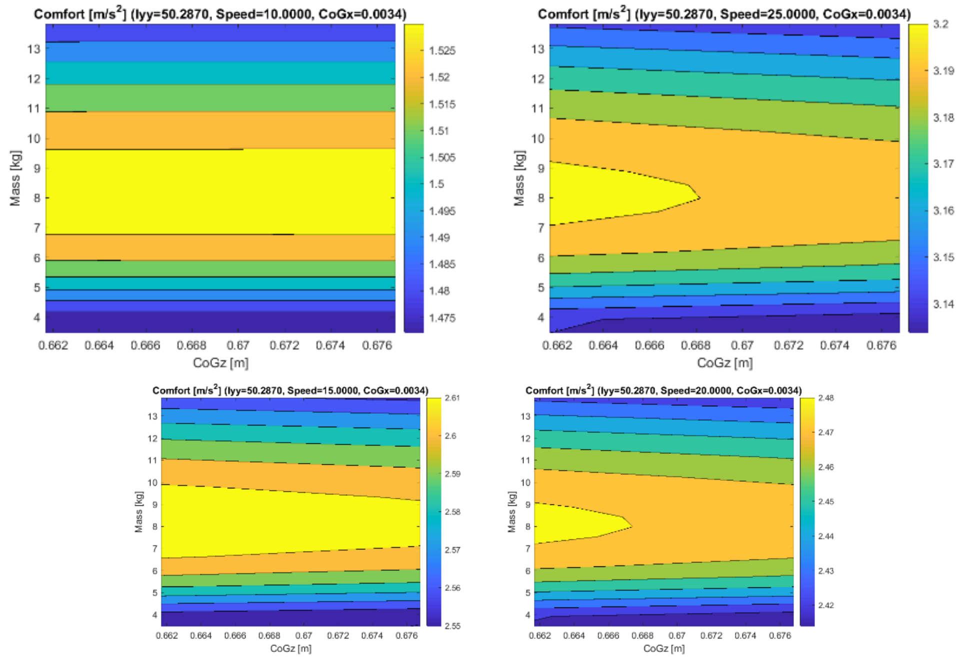

1.2.1.7 Variation with mass and CoGx

1.2.1.7.1 Varying I_{yy}

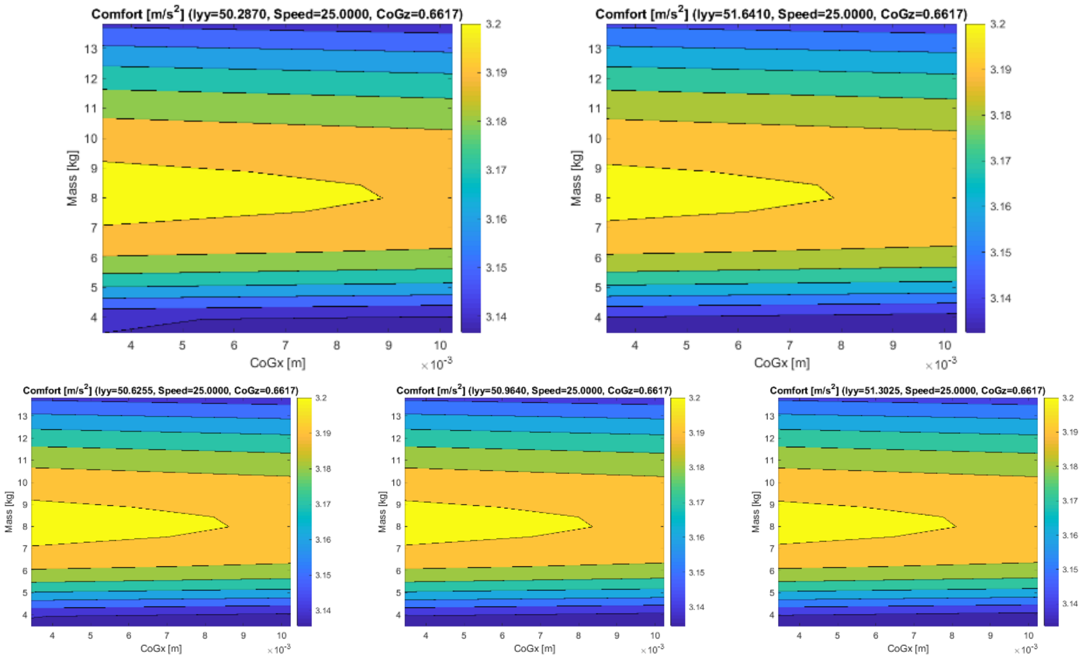

1.2.1.7.2 Varying CoGz

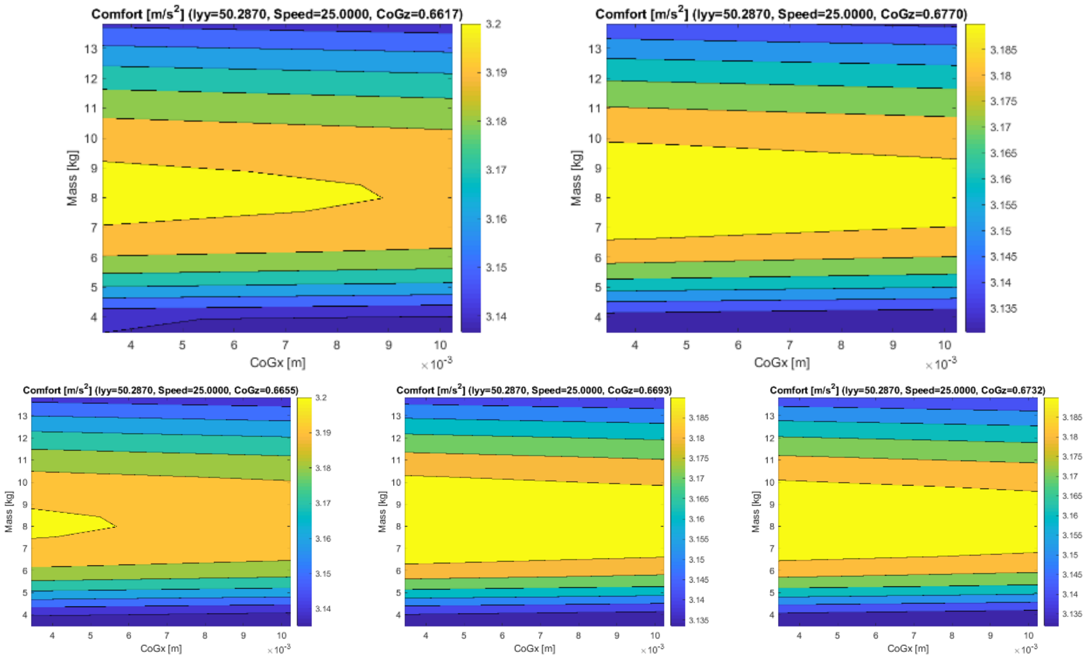

1.2.1.7.3 Varying speed

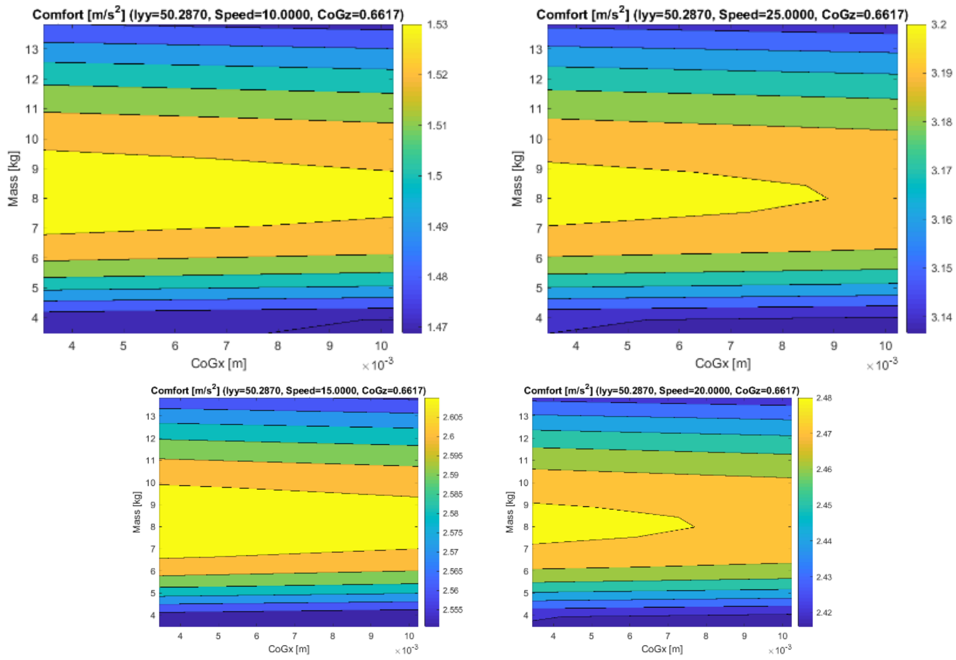

1.2.1.8 Variation with CoGz and CoGx

1.2.1.8.1 Varying I_{yy}

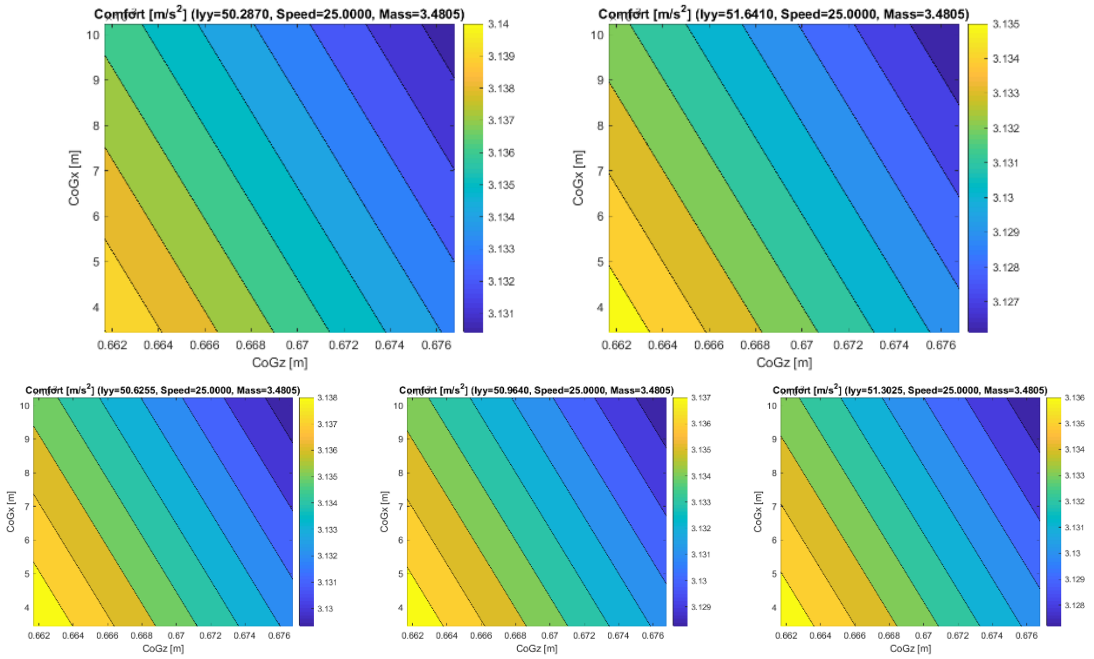

1.2.1.8.2 Varying mass

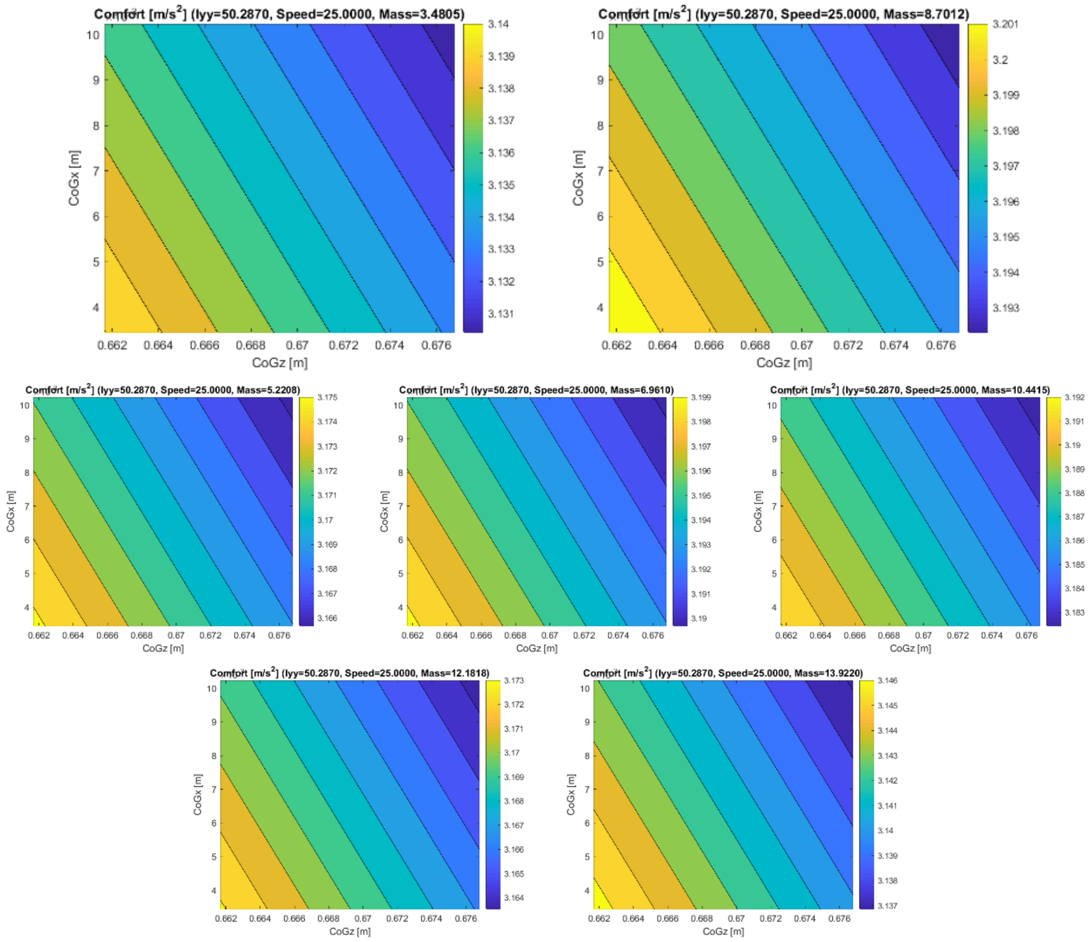

1.2.1.8.3 Varying speed

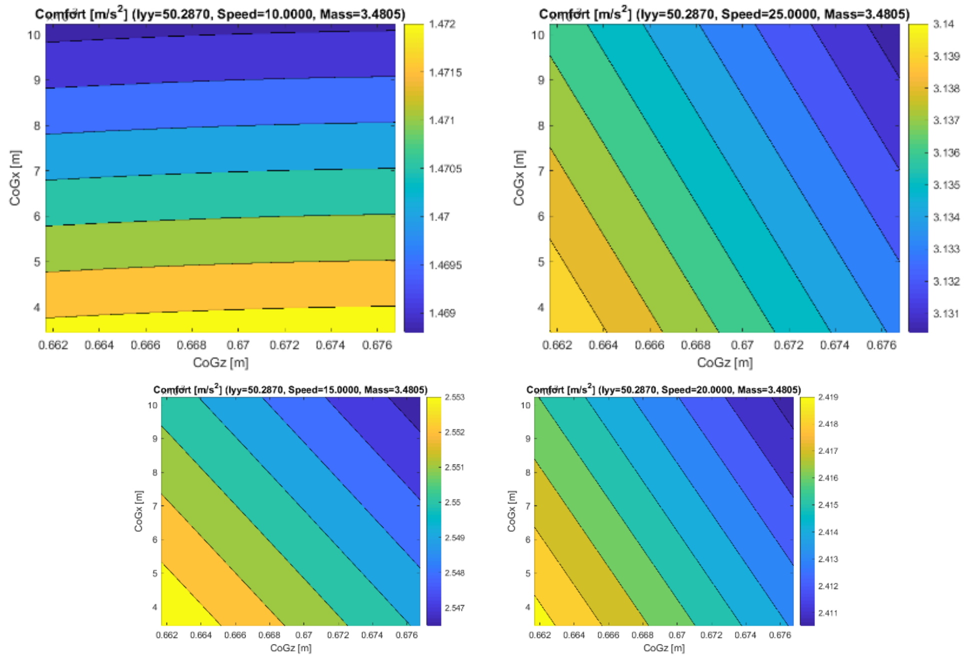

1.2.1.9 *Variation with CoGz and I_{yy}*

1.2.1.9.1 Varying CoGx

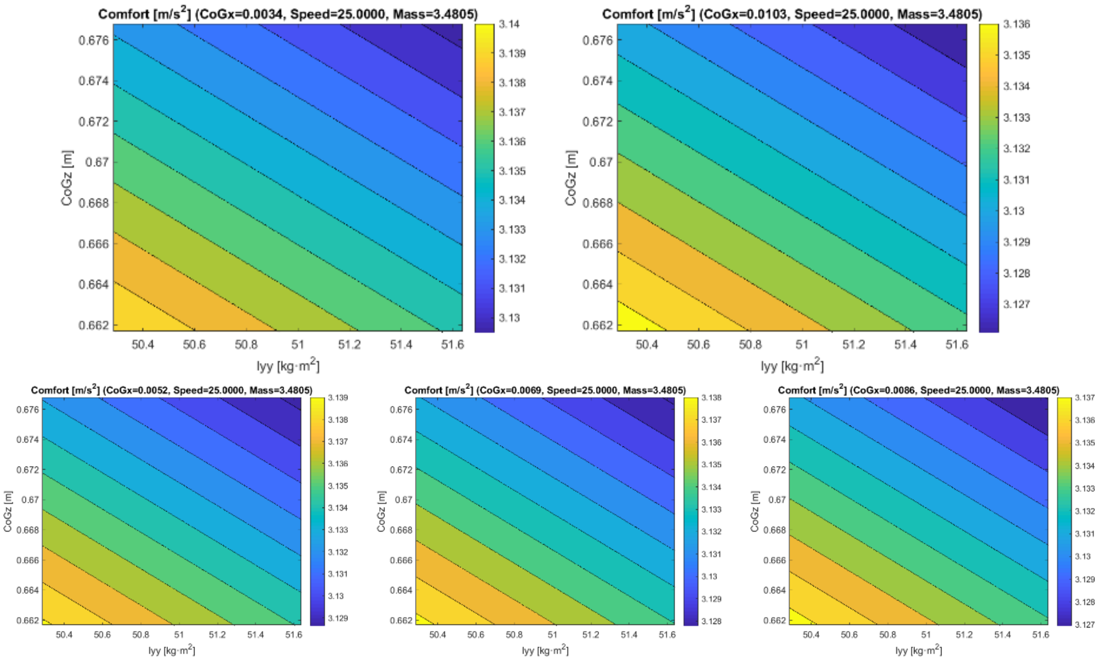

1.2.1.9.2 Varying mass

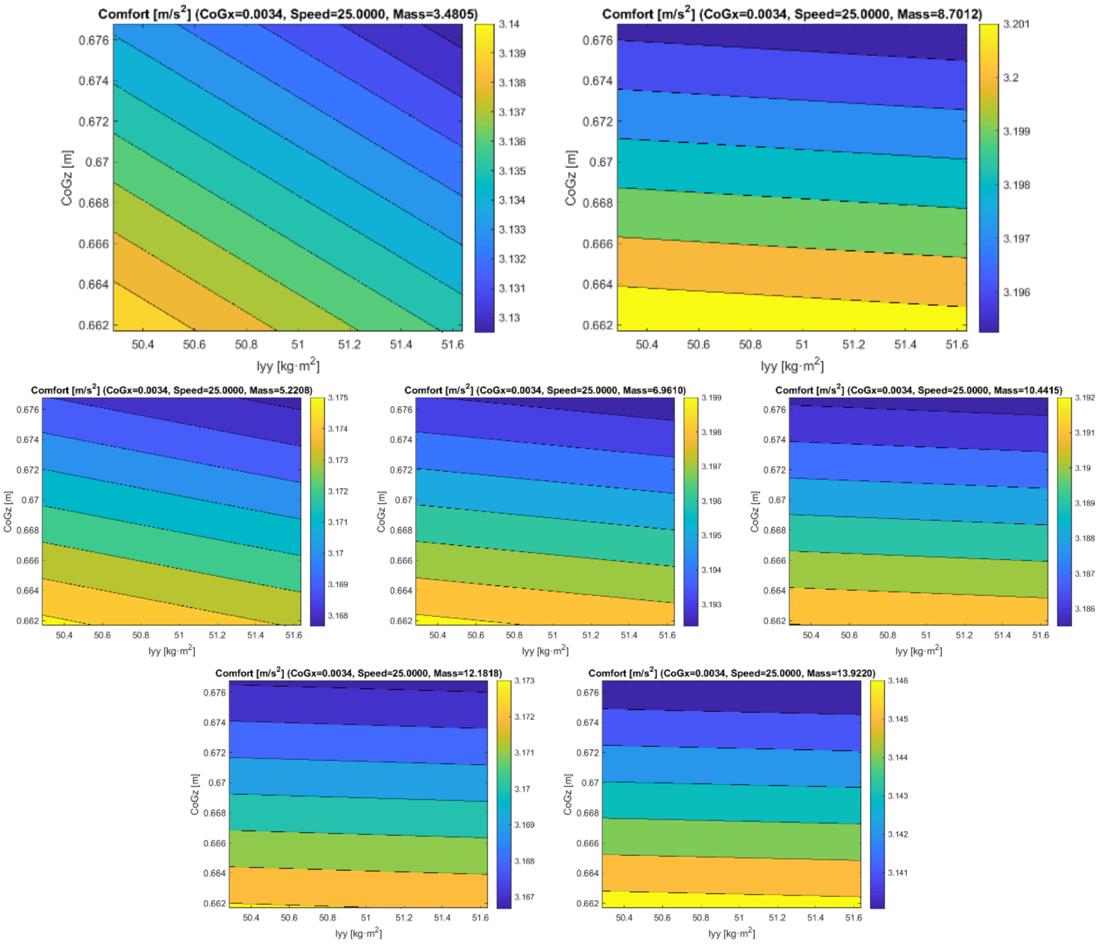

1.2.1.9.3 Varying speed

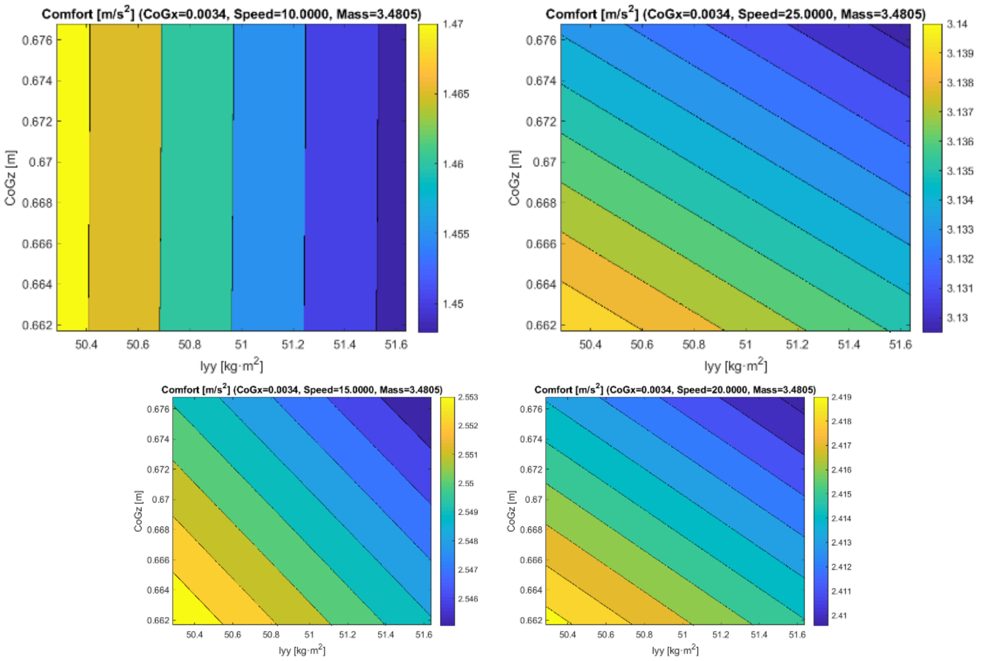

1.2.1.10 Variation with CoGx and I_{yy}

1.2.1.10.1 Varying CoGz

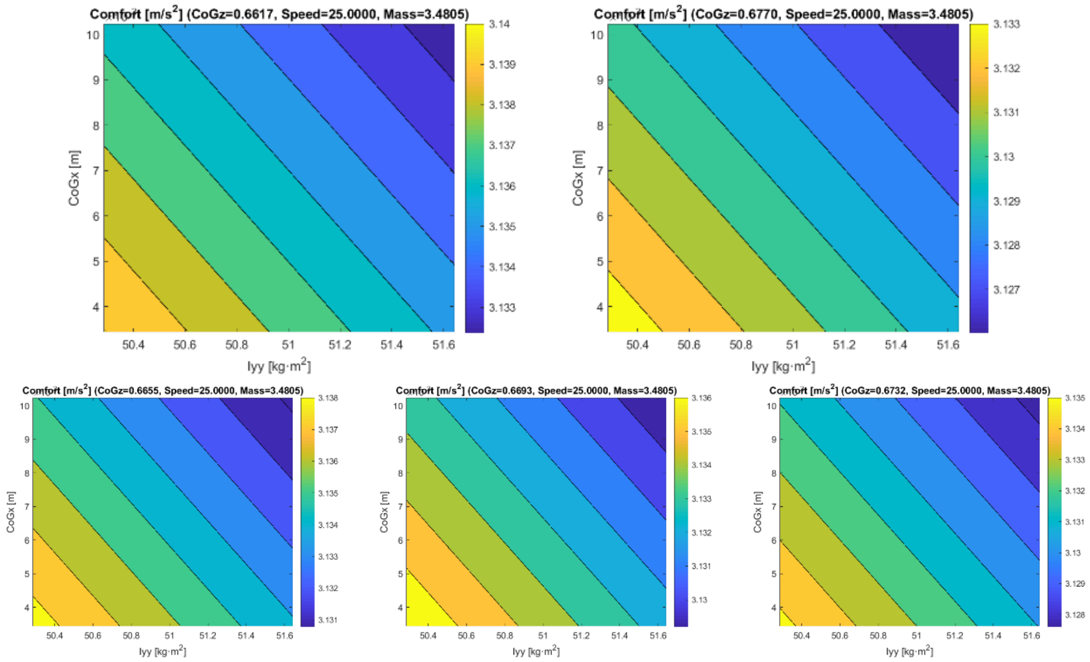

1.2.1.10.2 Varying mass

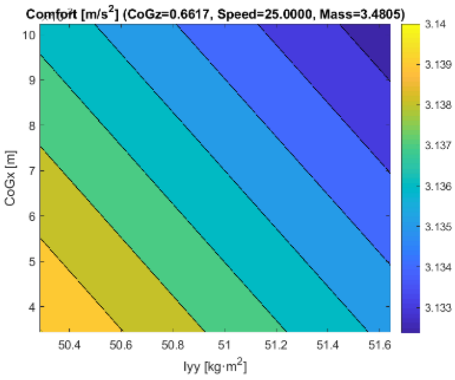

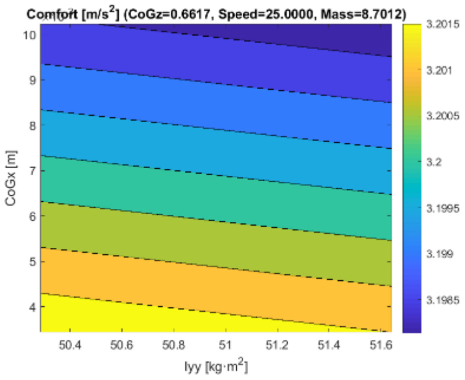

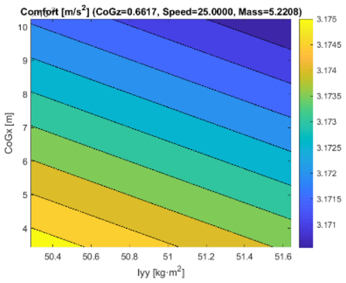

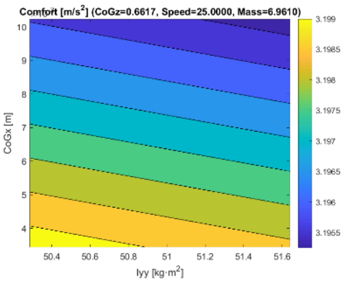

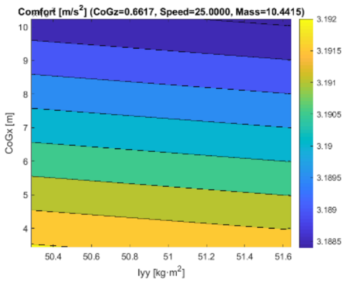

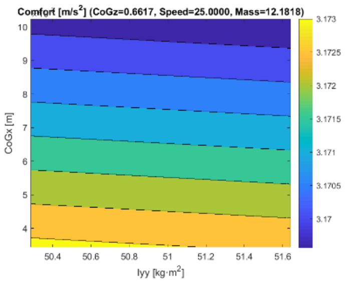

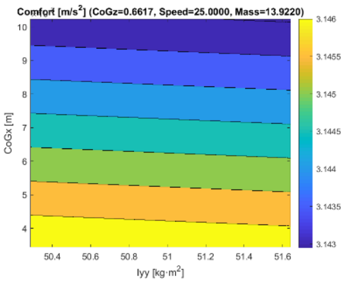

1.2.1.10.3 Varying speed

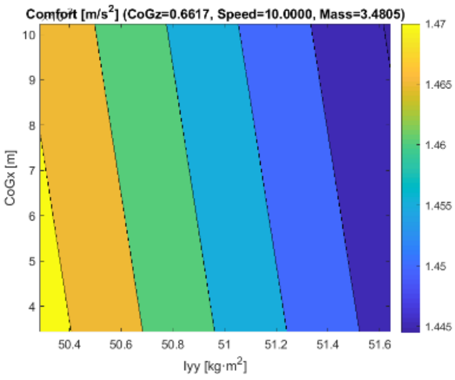

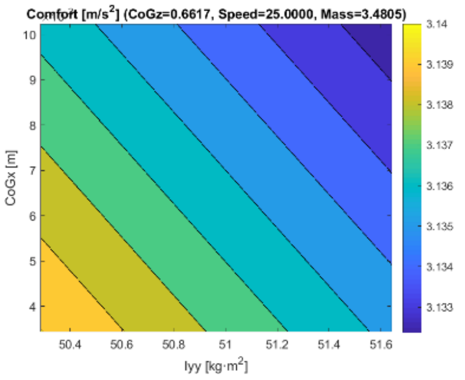

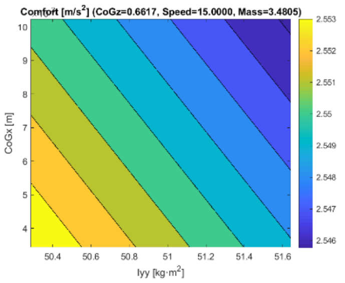

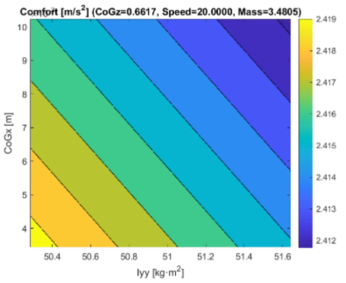

1.2.2 Raw model

1.2.2.1 Variation with speed and I_{yy}

1.2.2.1.1 Varying CoGz

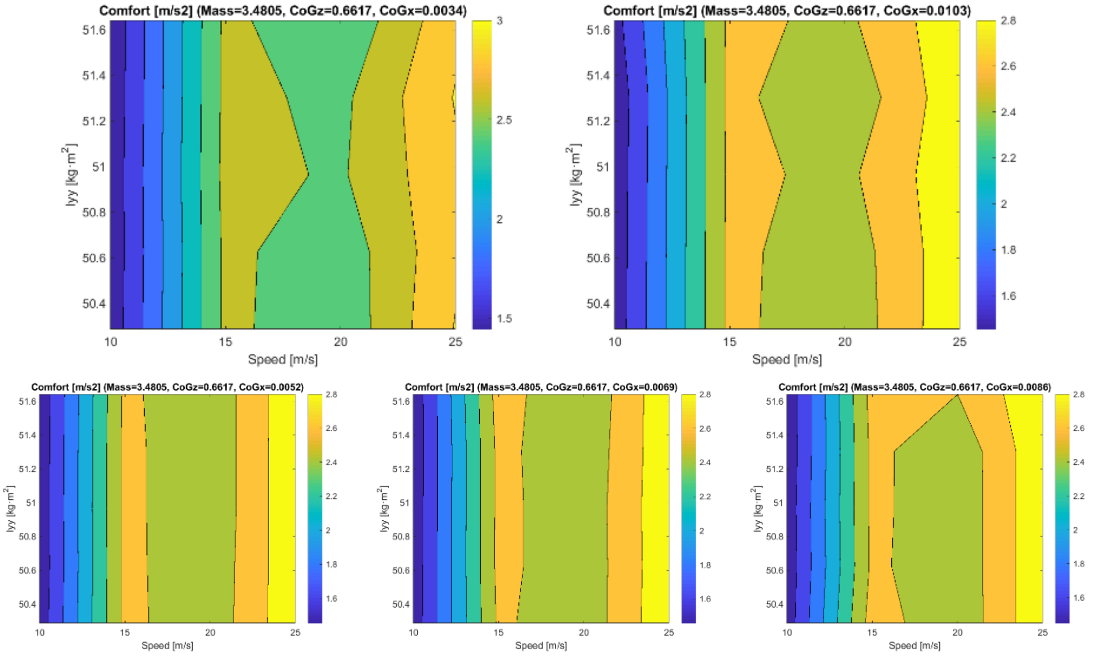

1.2.2.1.2 Varying CoGx

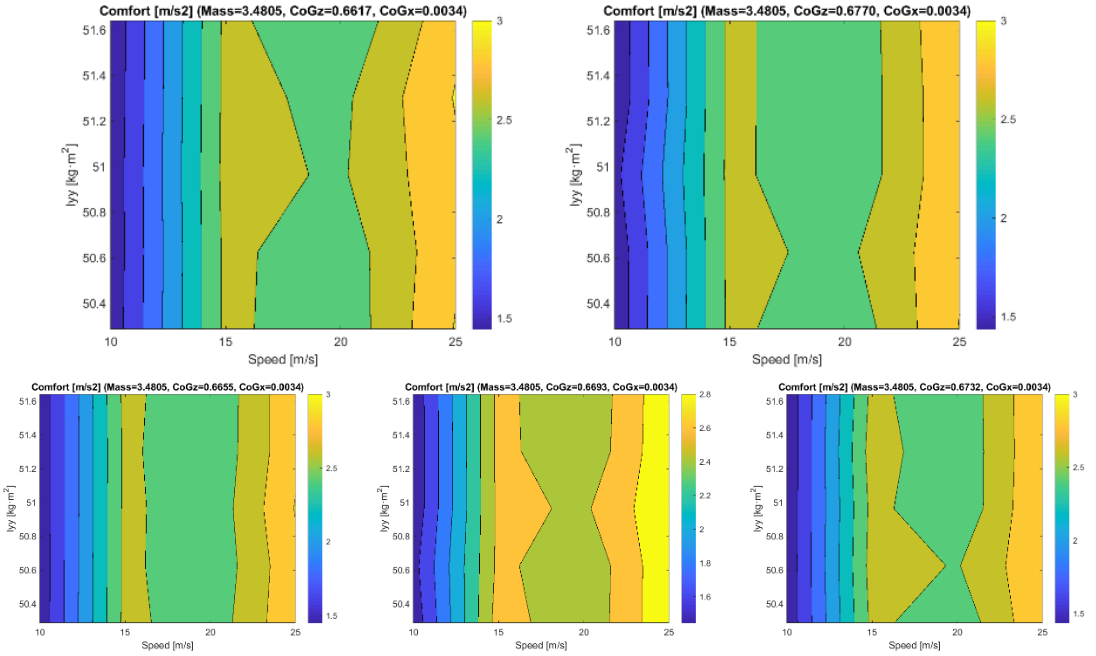

1.2.2.1.3 Varying mass

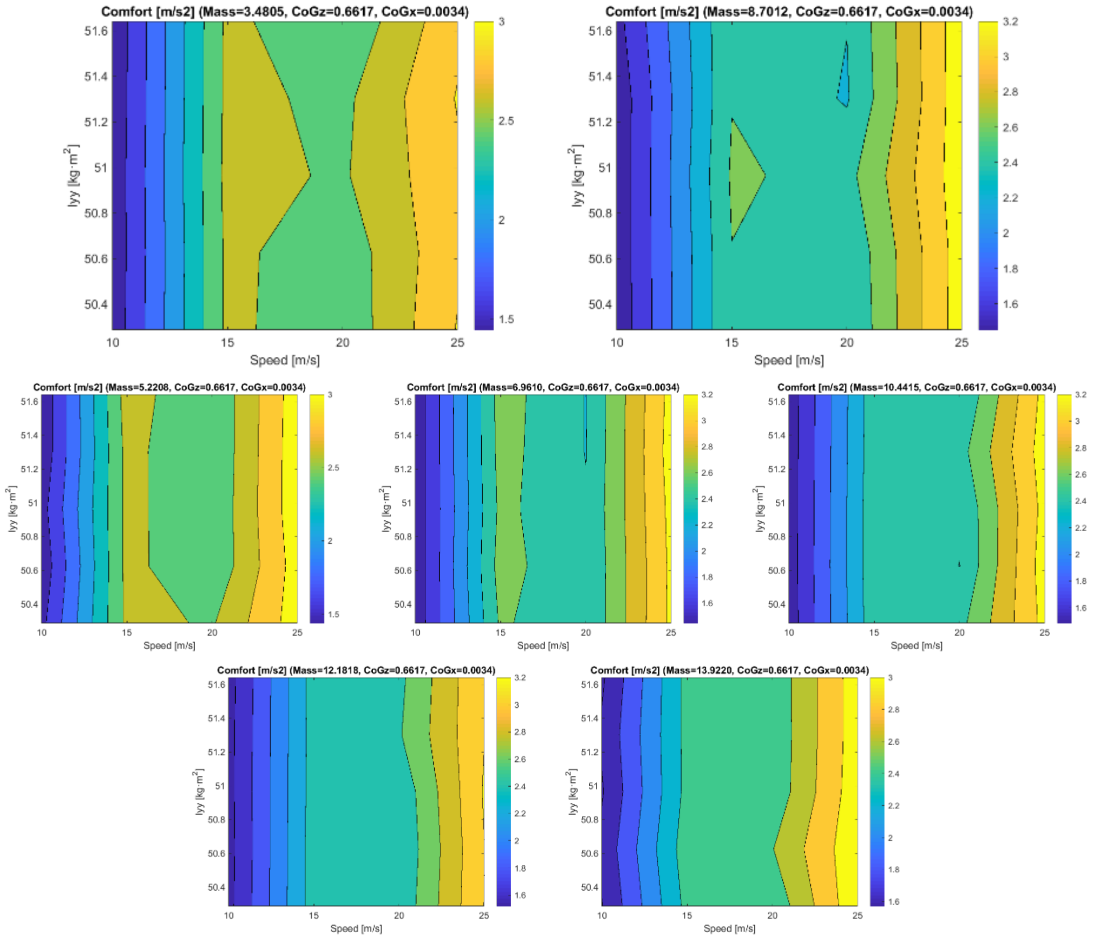

1.2.2.2 Variation with speed and CoGz

1.2.2.2.1 Varying I_{yy}

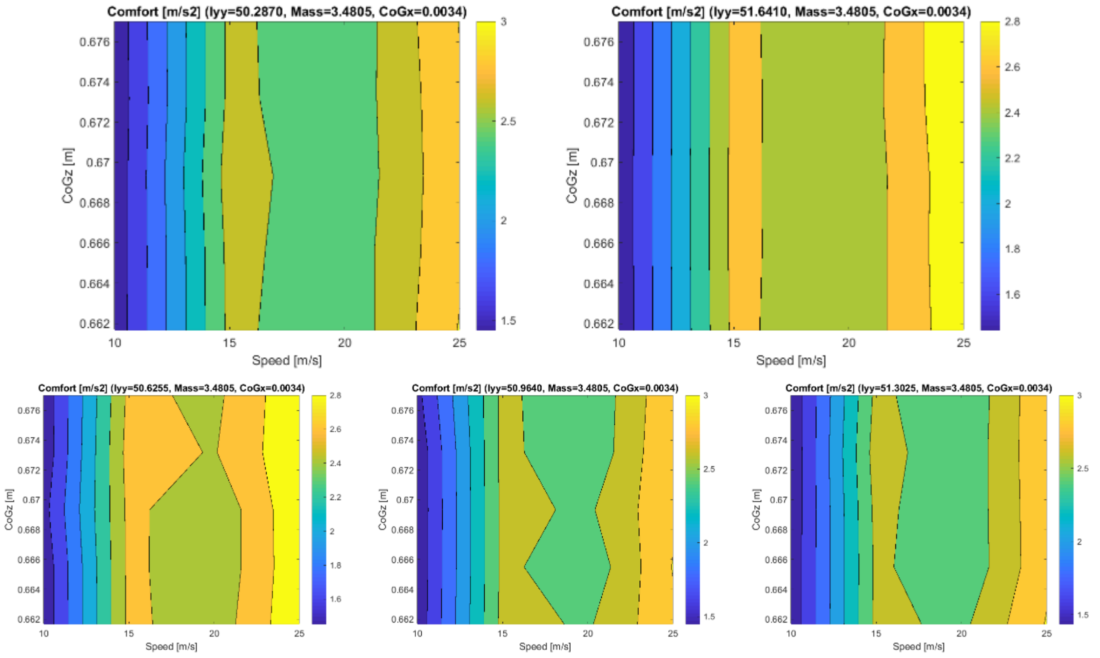

1.2.2.2.2 Varying CoGx

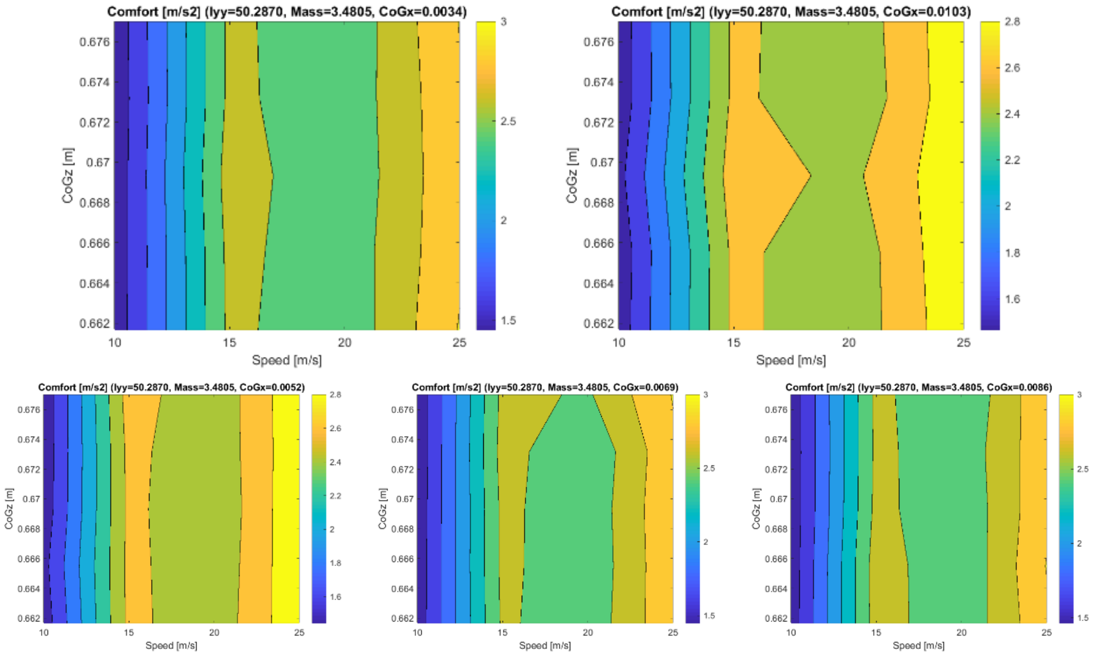

1.2.2.2.3 Varying mass

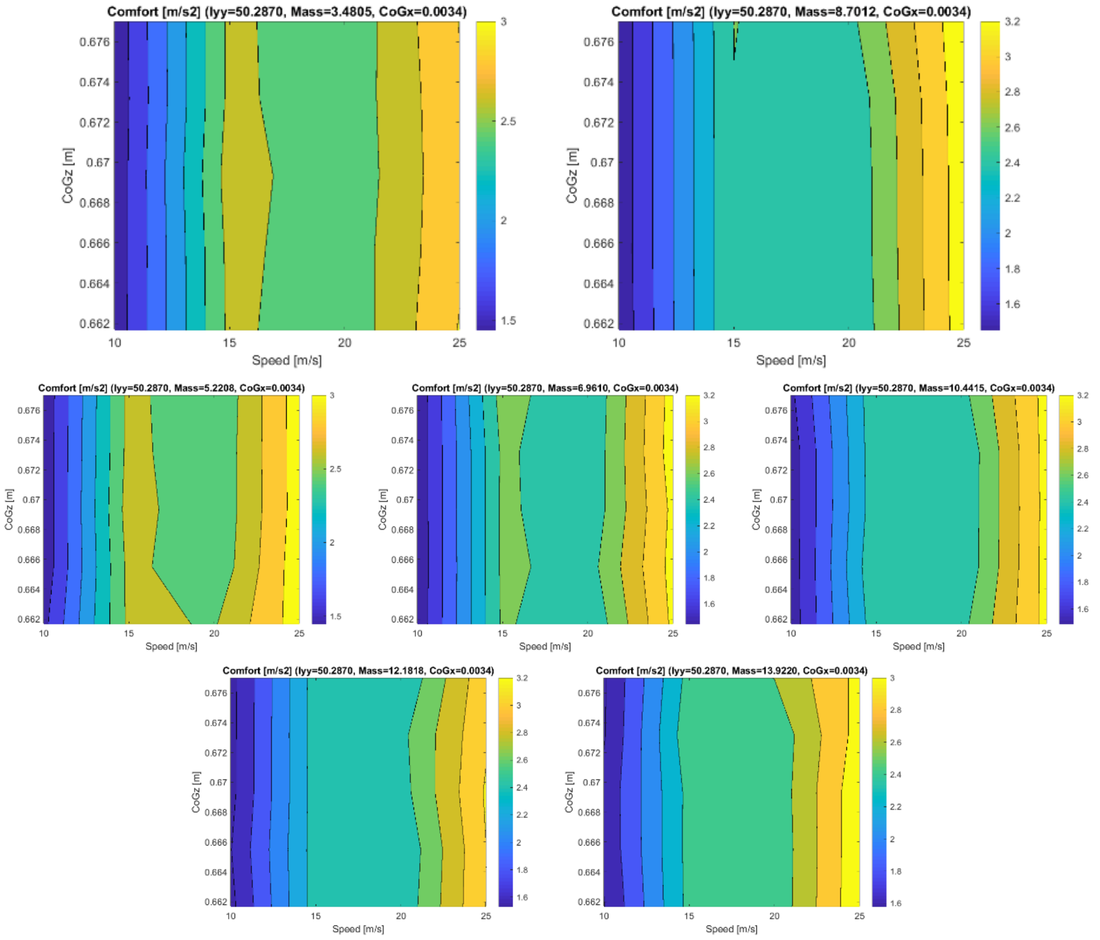

1.2.2.3 Variation with speed and CoGx

1.2.2.3.1 Varying I_{yy}

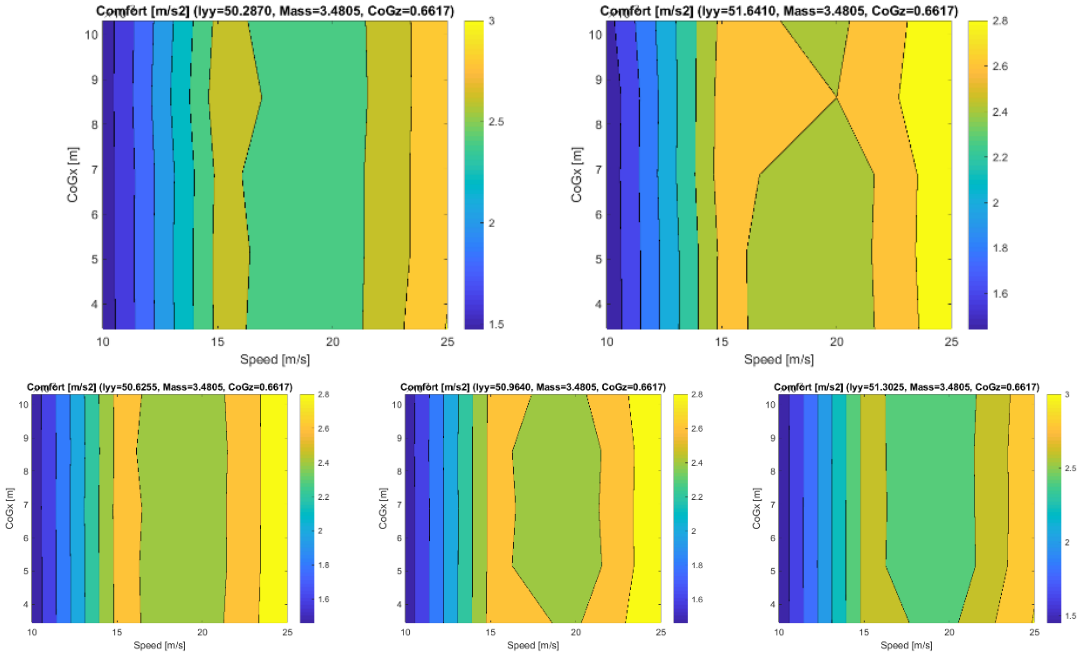

1.2.2.3.2 Varying CoGz

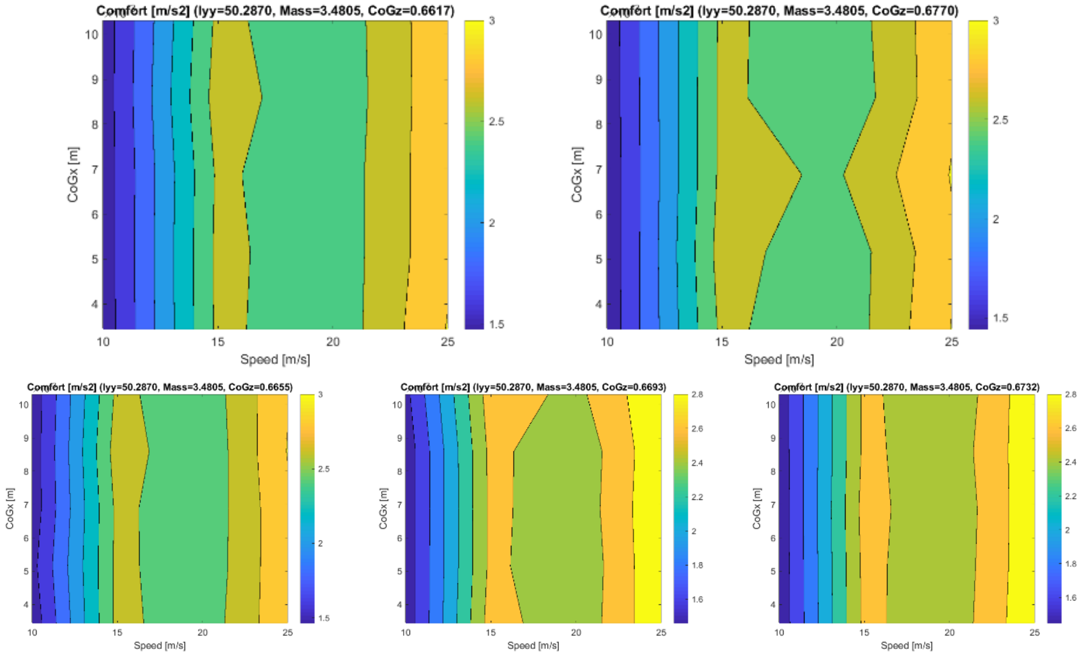

1.2.2.3.3 Varying mass

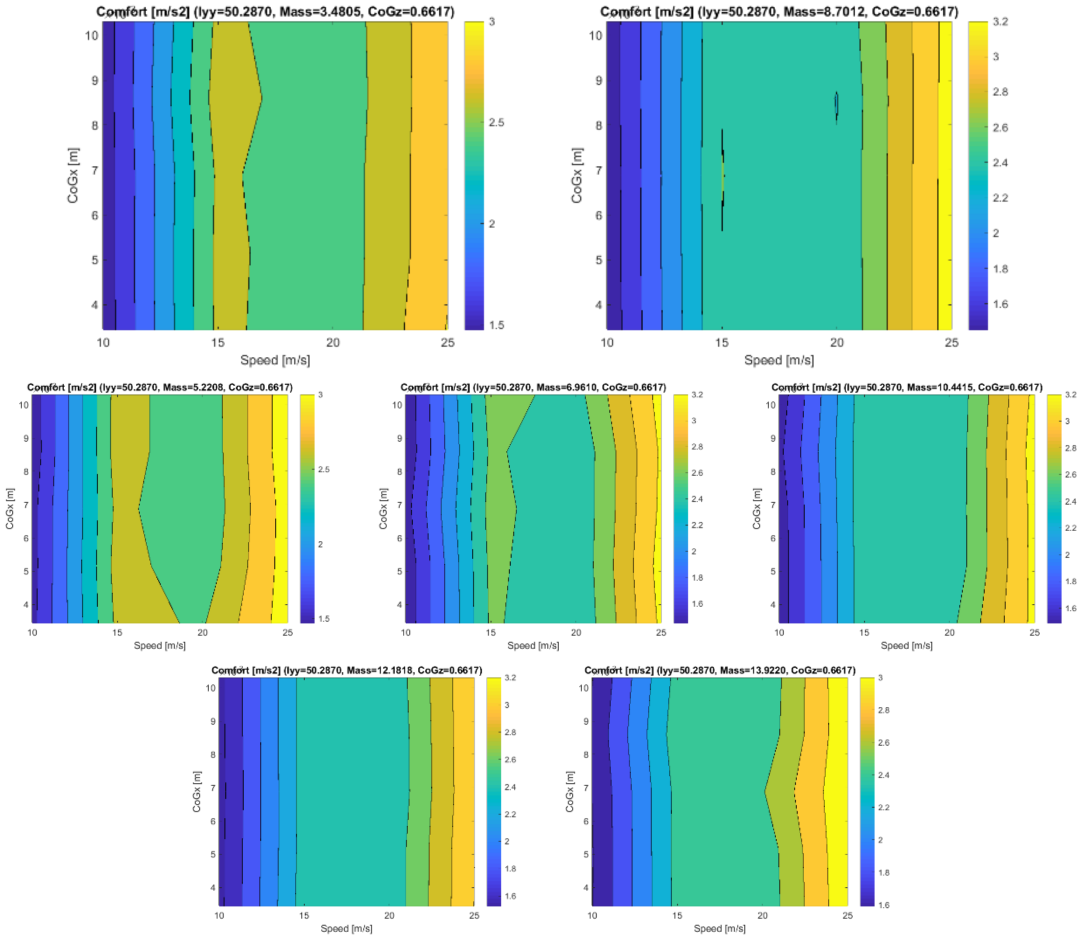

1.2.2.4 Variation with speed and mass

1.2.2.4.1 Varying I_{yy}

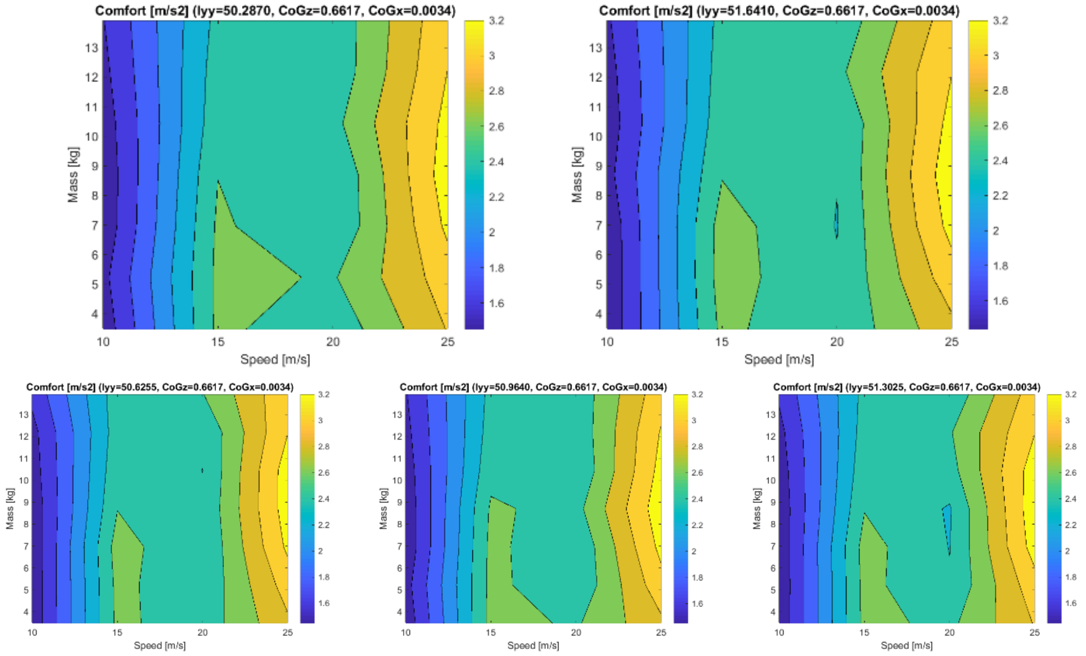

1.2.2.4.2 Varying CoGz

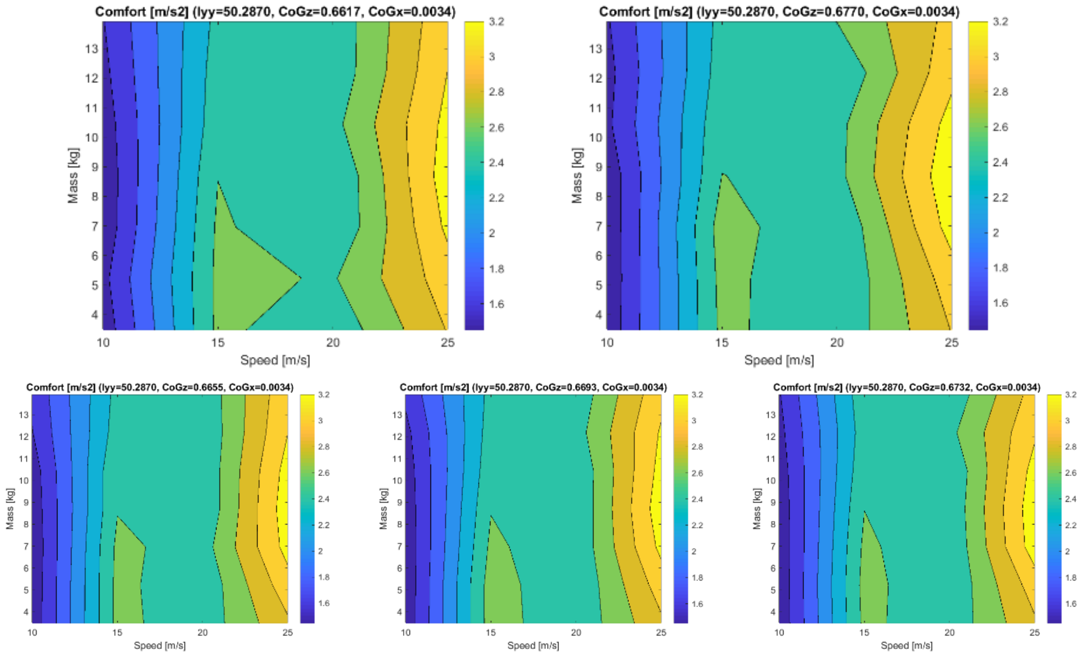

1.2.2.4.3 Varying CoGx

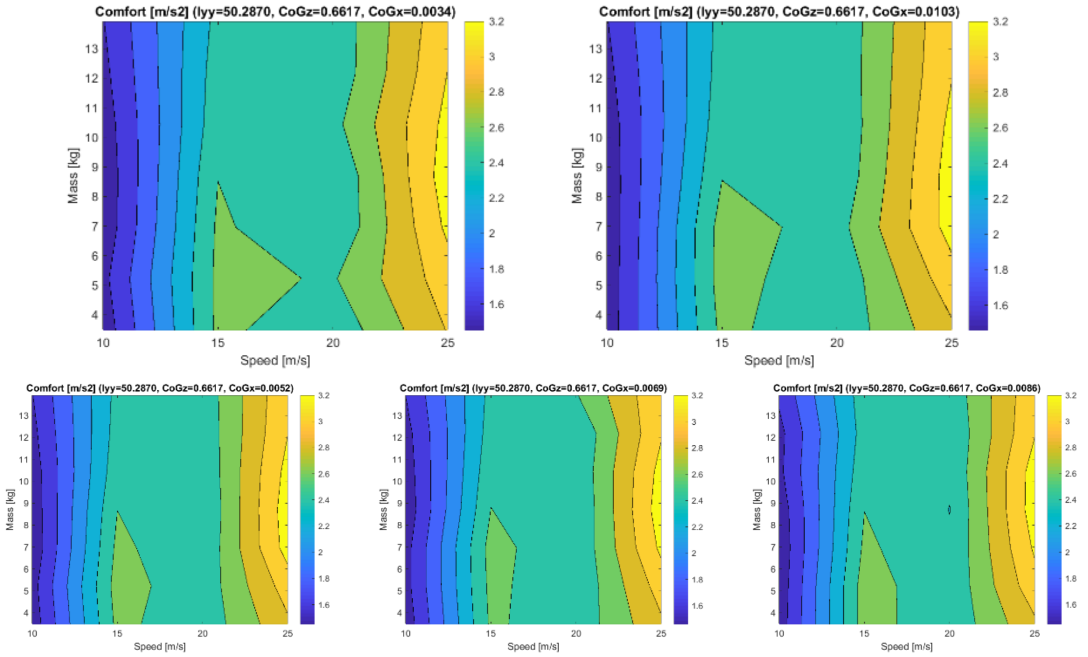

1.2.2.5 *Variation with mass and I_{yy}*

1.2.2.5.1 Varying CoGz

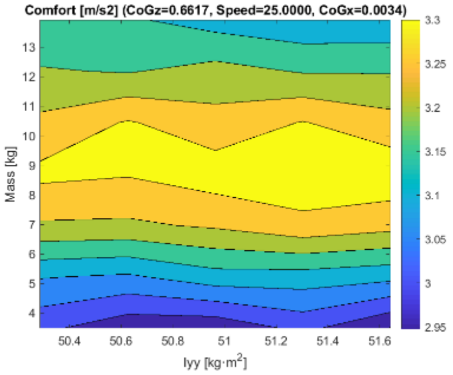

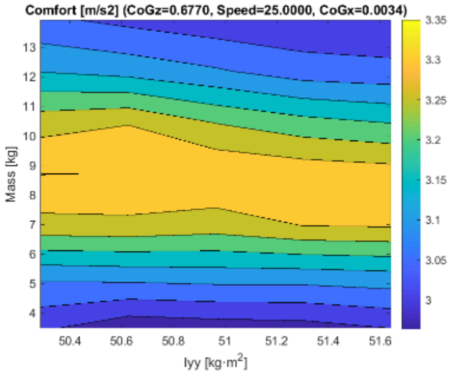

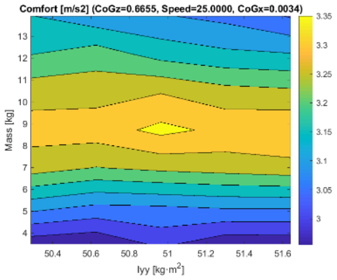

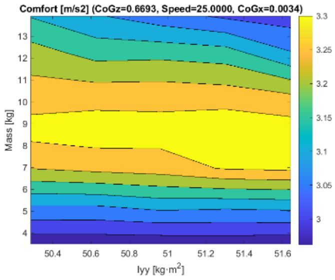

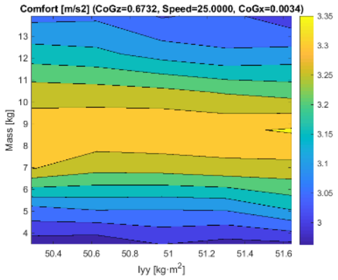

1.2.2.5.2 Varying CoGx

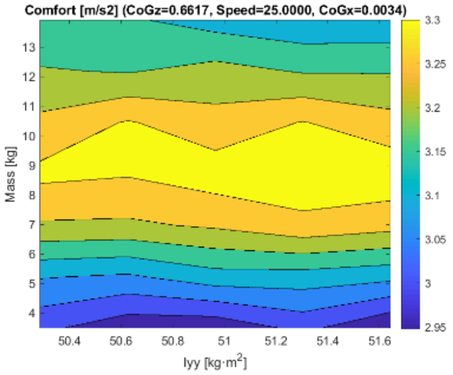

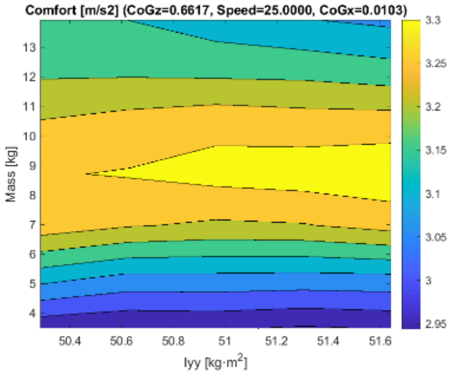

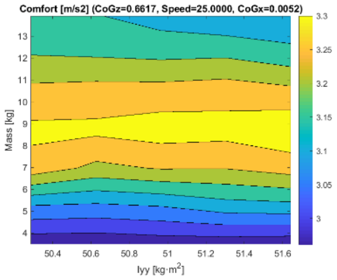

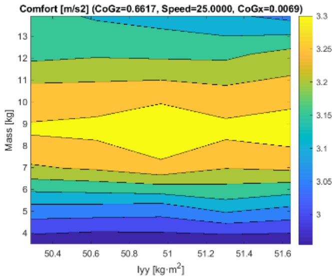

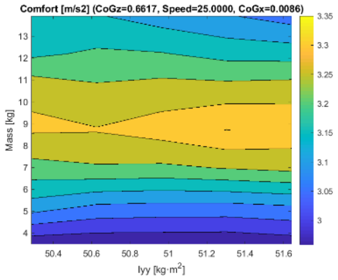

1.2.2.5.3 Varying speed

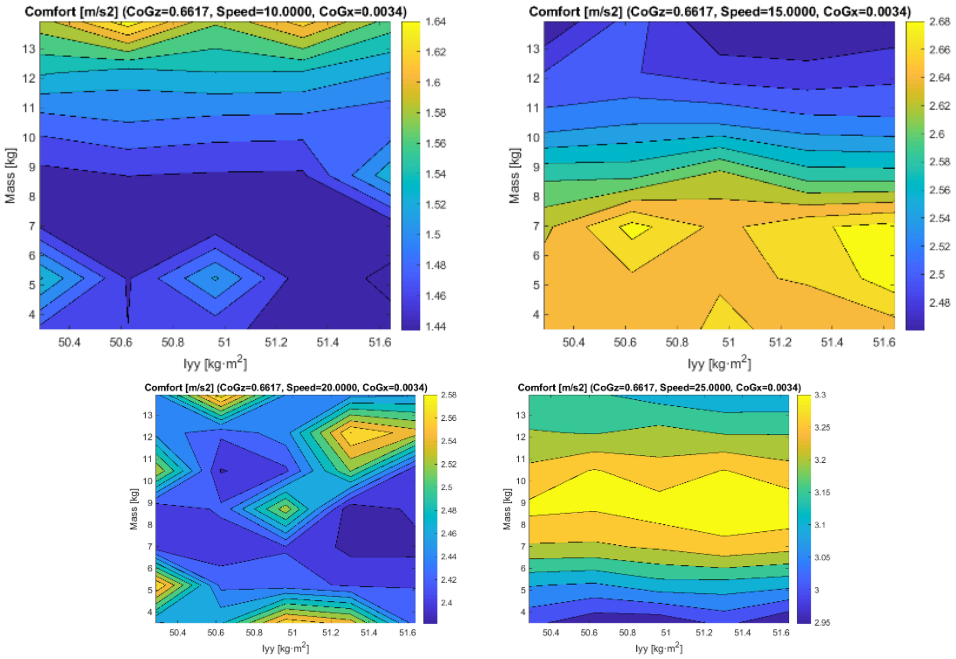

1.2.2.6 Variation with mass and CoGz

1.2.2.6.1 Varying I_{yy}

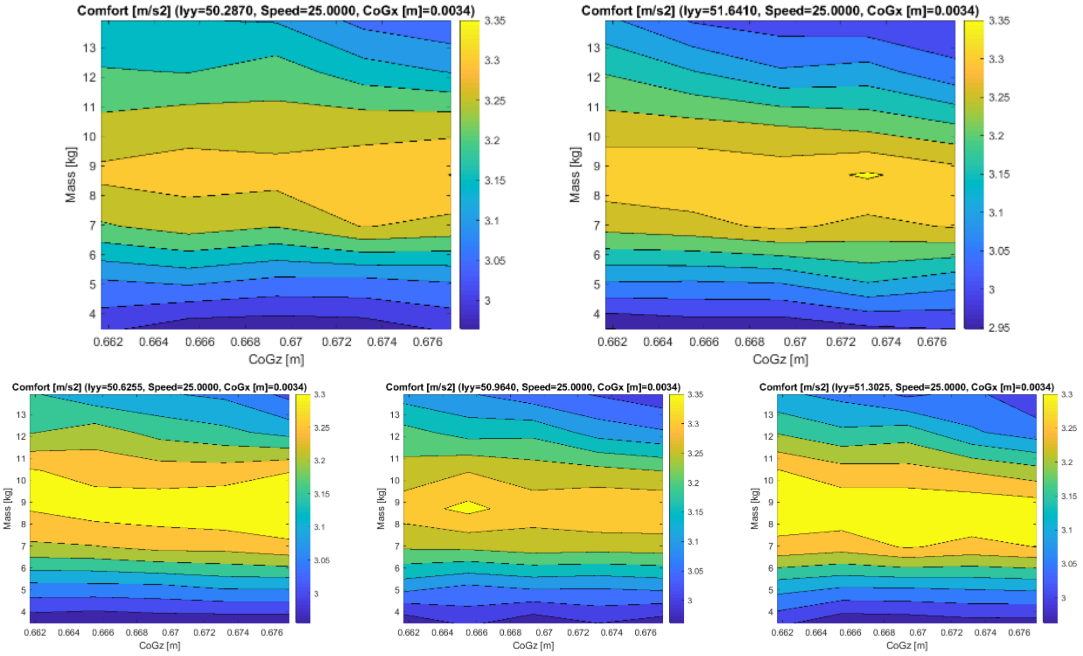

1.2.2.6.2 Varying CoGx

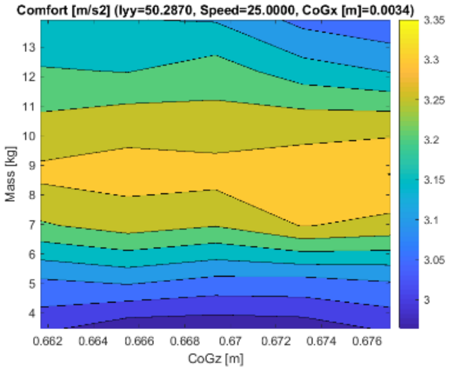

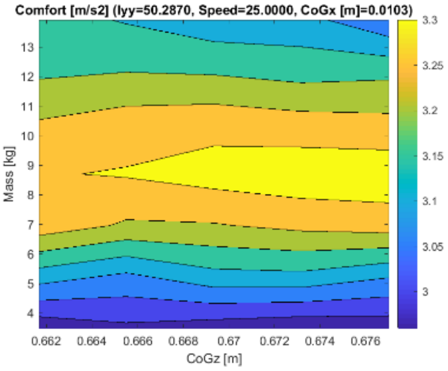

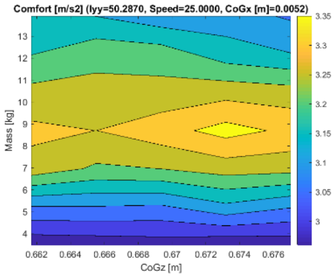

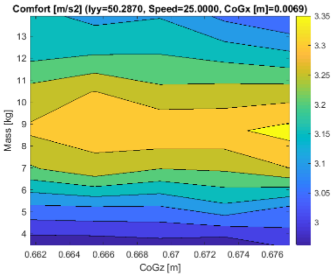

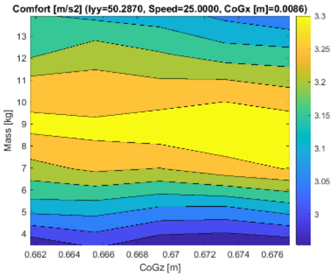

1.2.2.6.3 Varying speed

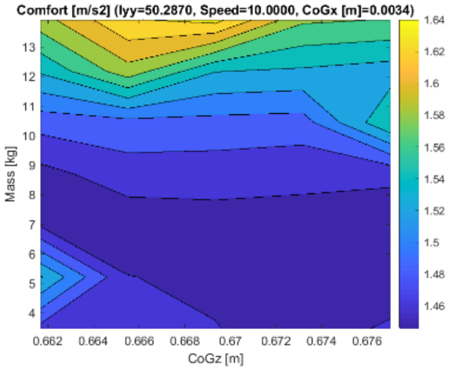

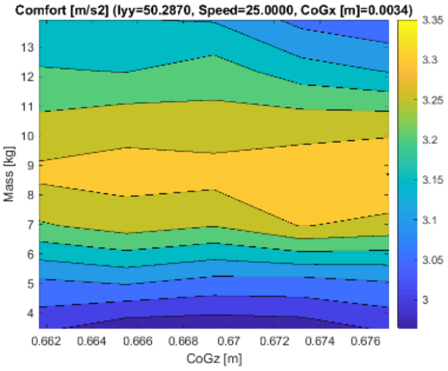

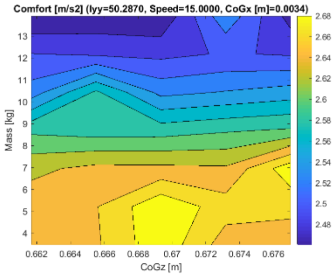

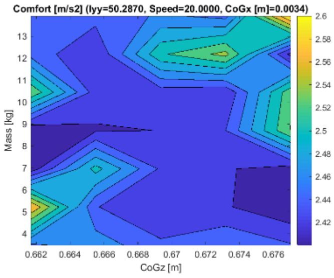

1.2.2.7 *Variation with mass and CoGx*

1.2.2.7.1 Varying I_{yy}

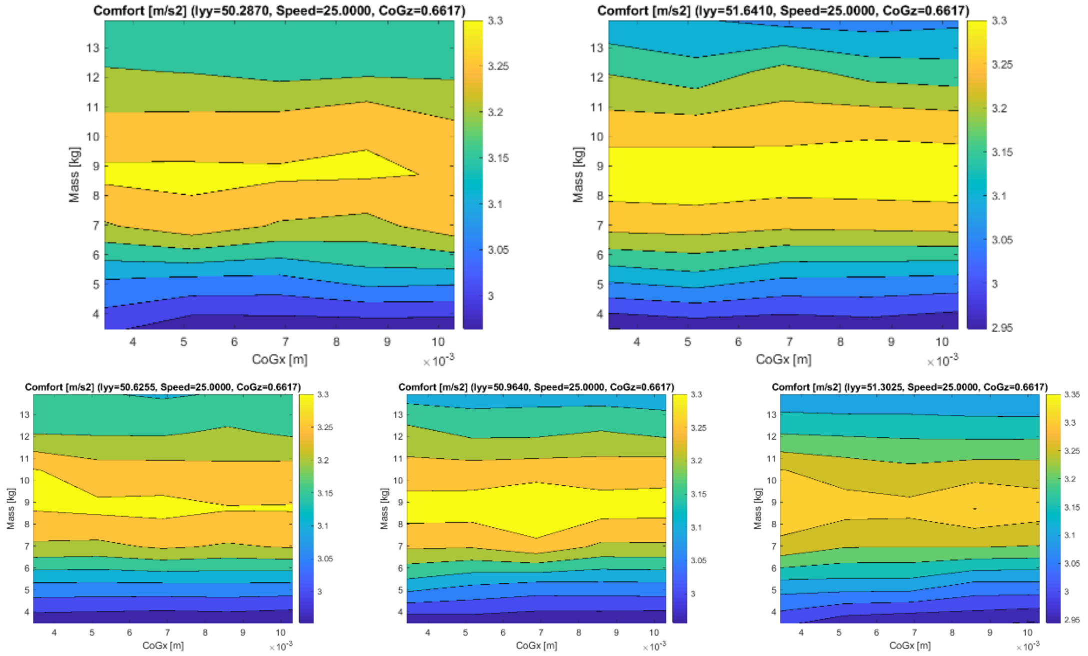

1.2.2.7.2 Varying CoGz

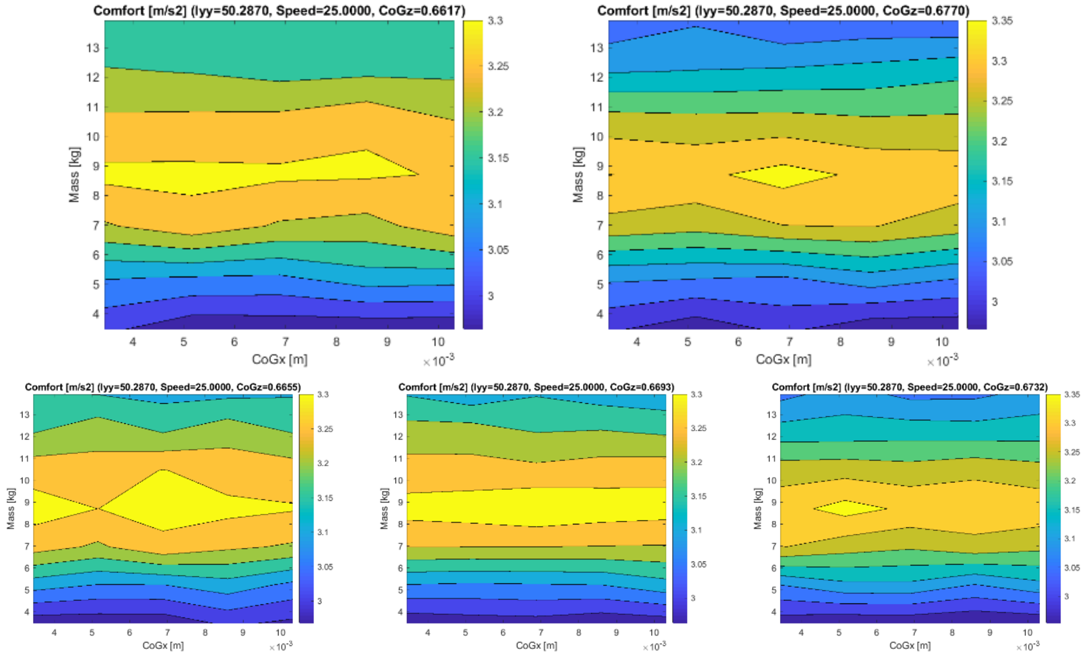

1.2.2.7.3 Varying speed

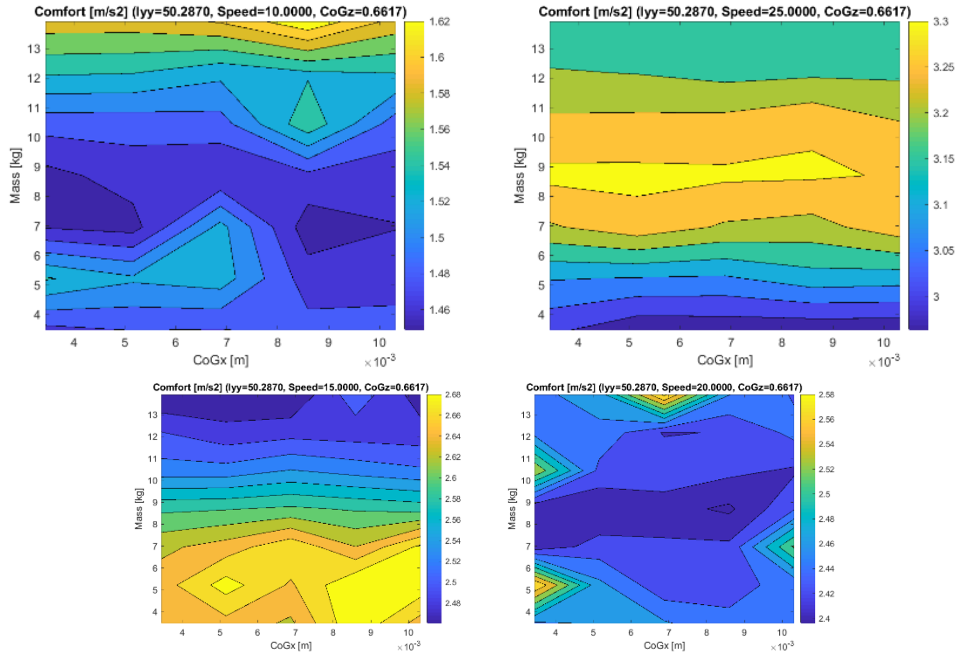

1.2.2.8 Variation with CoGz and CoGx

1.2.2.8.1 Varying I_{yy}

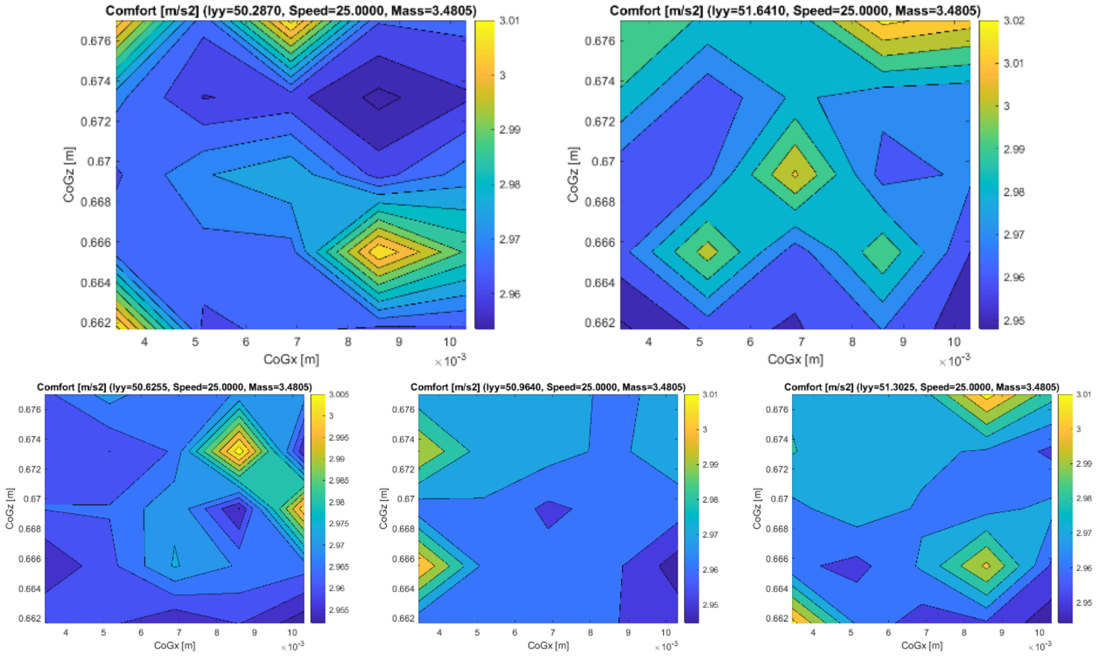

1.2.2.8.2 Varying mass

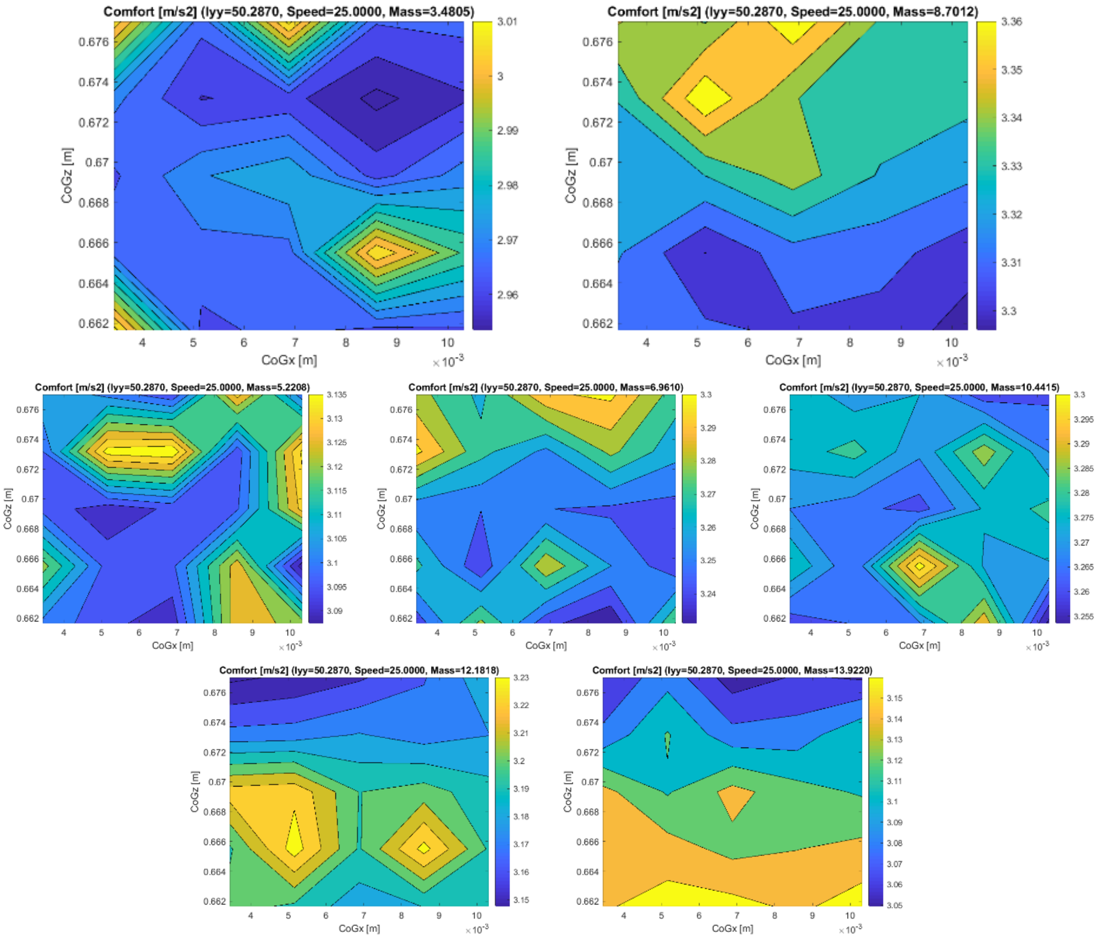

1.2.2.8.3 Varying speed

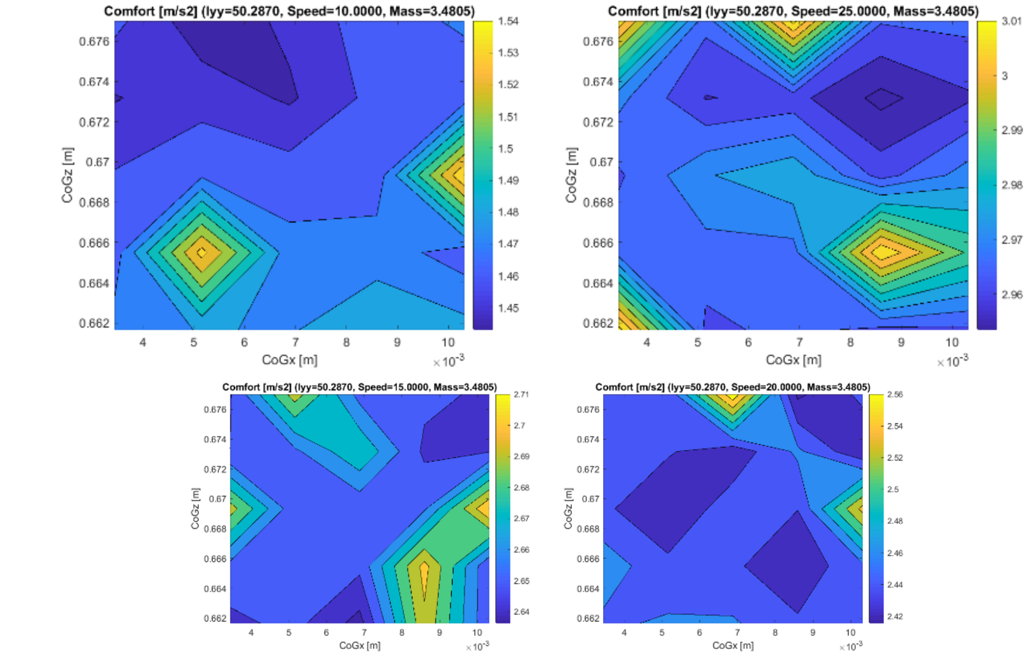

1.2.2.9 Variation with CoGz and I_{yy}

1.2.2.9.1 Varying CoGx

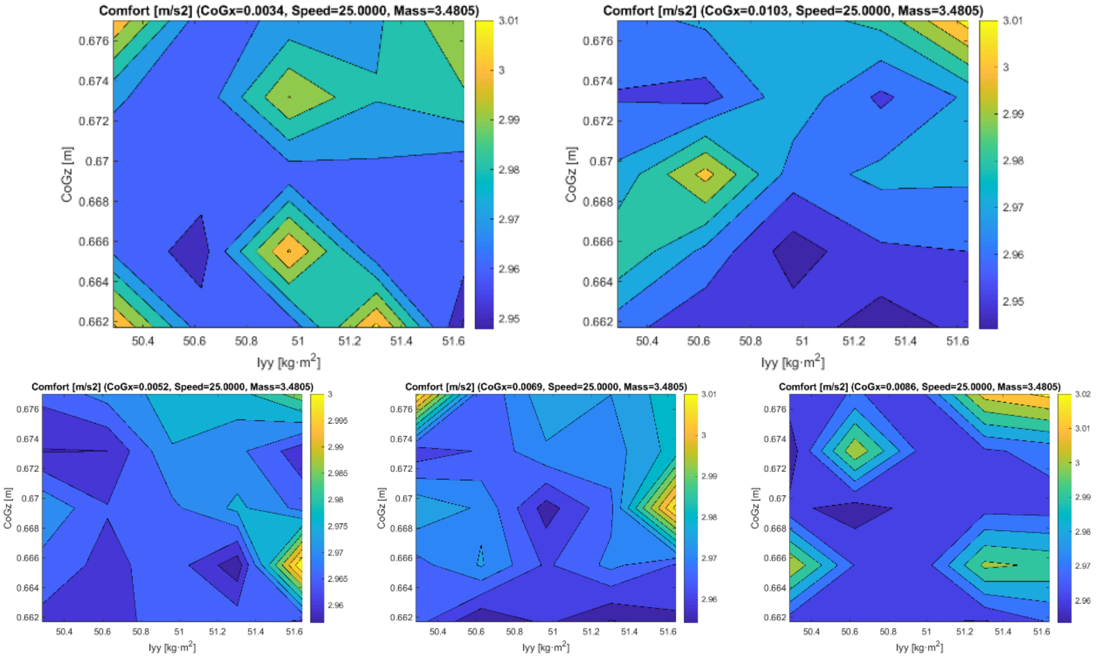

1.2.2.9.2 Varying mass

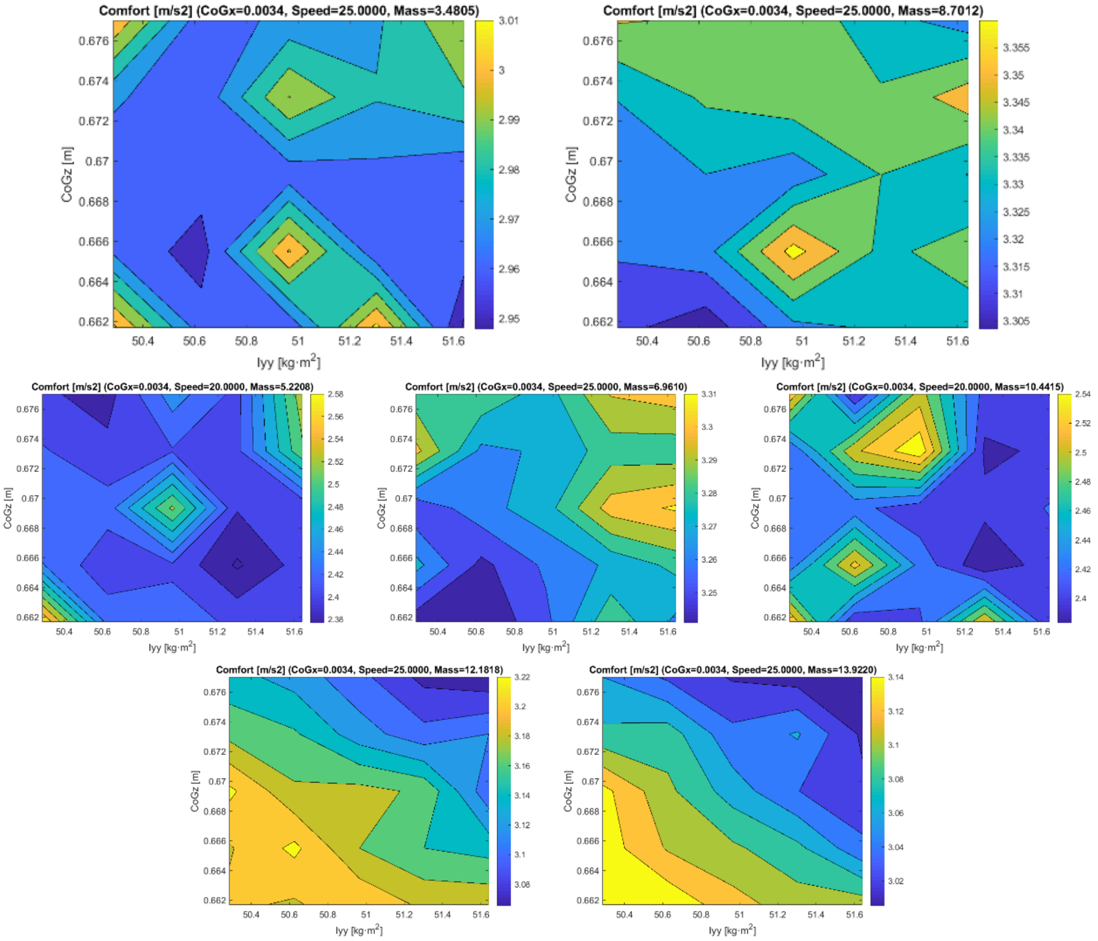

1.2.2.9.3 Varying speed

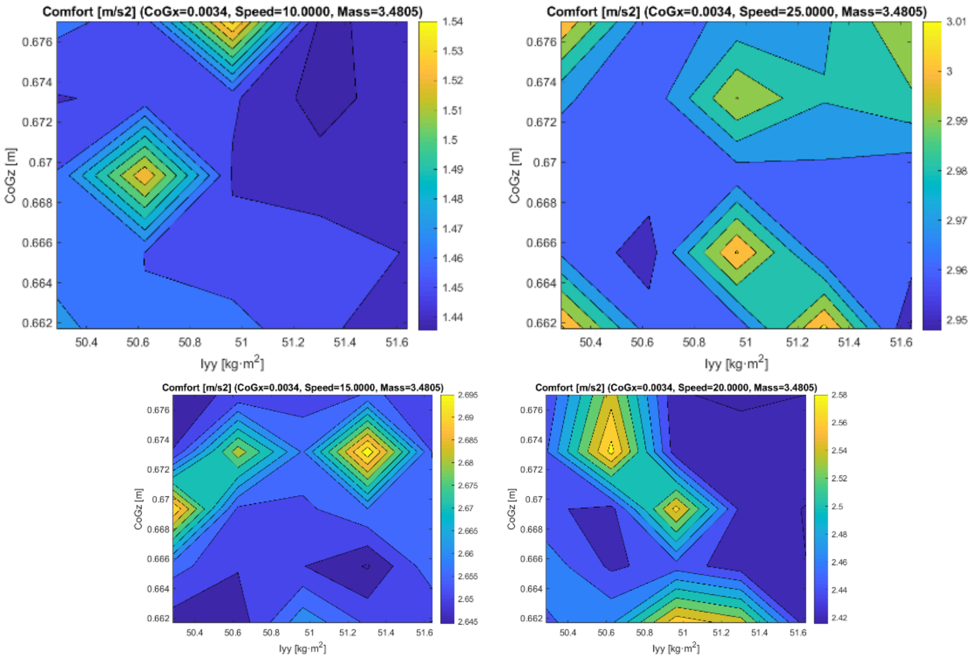

1.2.2.10 Variation with CoGx and I_{yy}

1.2.2.10.1 Varying CoGz

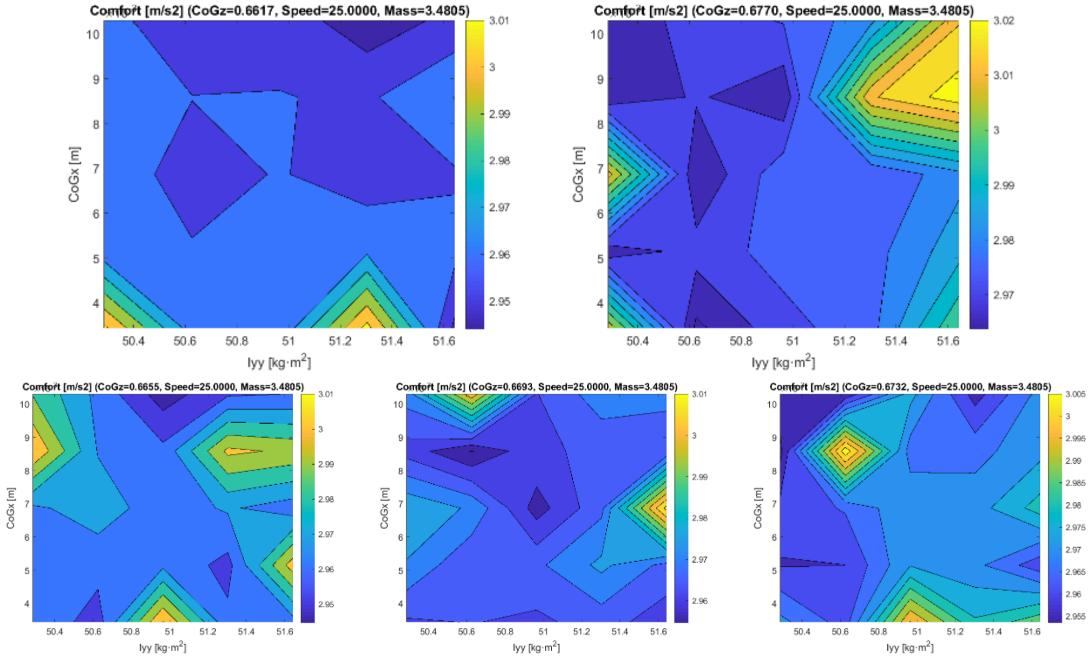

1.2.2.10.2 Varying mass

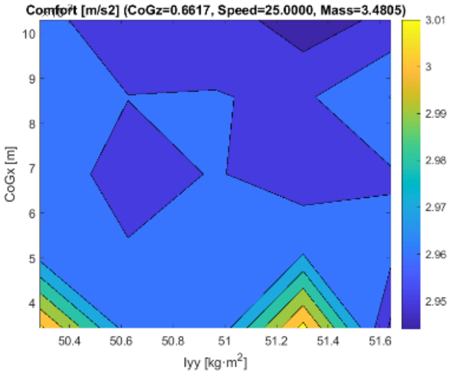

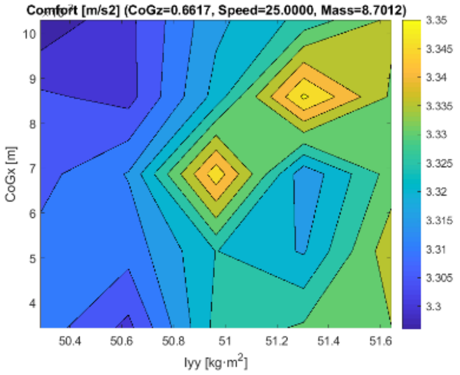

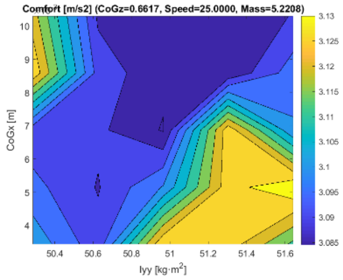

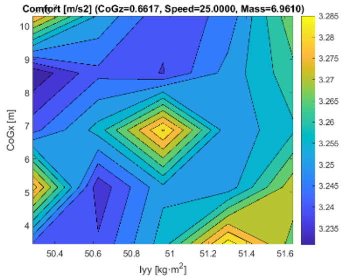

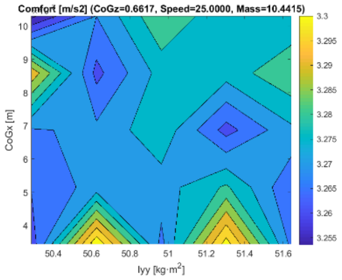

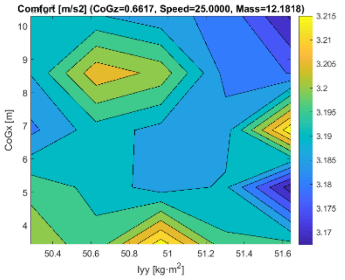

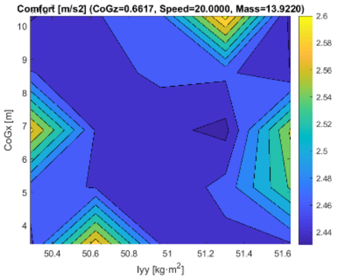

1.2.2.10.3 Varying speed

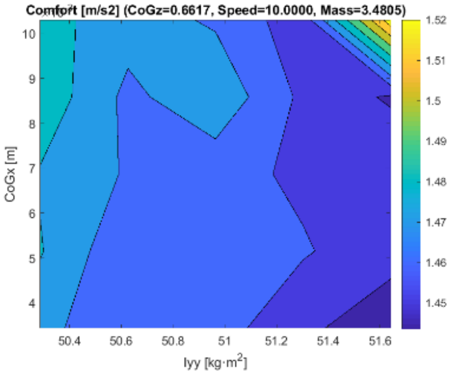

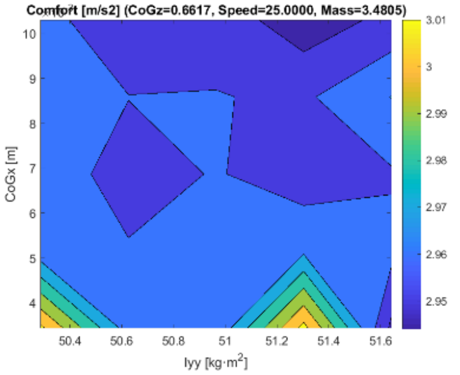

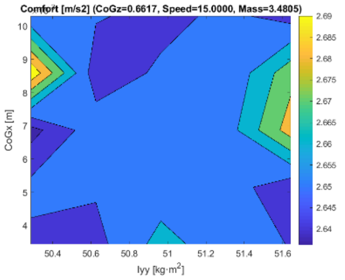

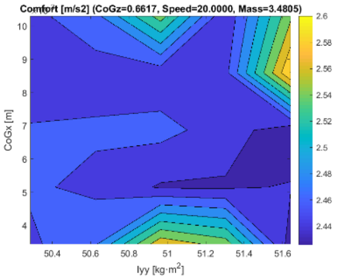

Supplement: Supplementary file 1 [file sensors-24-00399-s001.zip › sensors-2784581-supplementary.pdf]
